# Supplementary material for: Sono-activable and biocatalytic 3D-printed scaffolds for intelligently sequential therapies in osteosarcoma eradication and defect regeneration
Source: Nat Commun. 2025 Jul 4;16:6150. doi: 10.1038/s41467-025-61377-x (PMC12229518; doi:10.1038/s41467-025-61377-x)
Supplement: Supplementary file 1 — Supplementary Information [file 41467_2025_61377_MOESM1_ESM.pdf]

Supplementary Information for

Sono-activable and biocatalytic 3D-printed scaffolds for intelligently sequential therapies in osteosarcoma eradication and defect regeneration

This PDF file includes:

Supplementary Figures 1 to 79

Supplementary Tables 1 to 4

Supplementary Methods

Supplementary References

Supplementary Figures

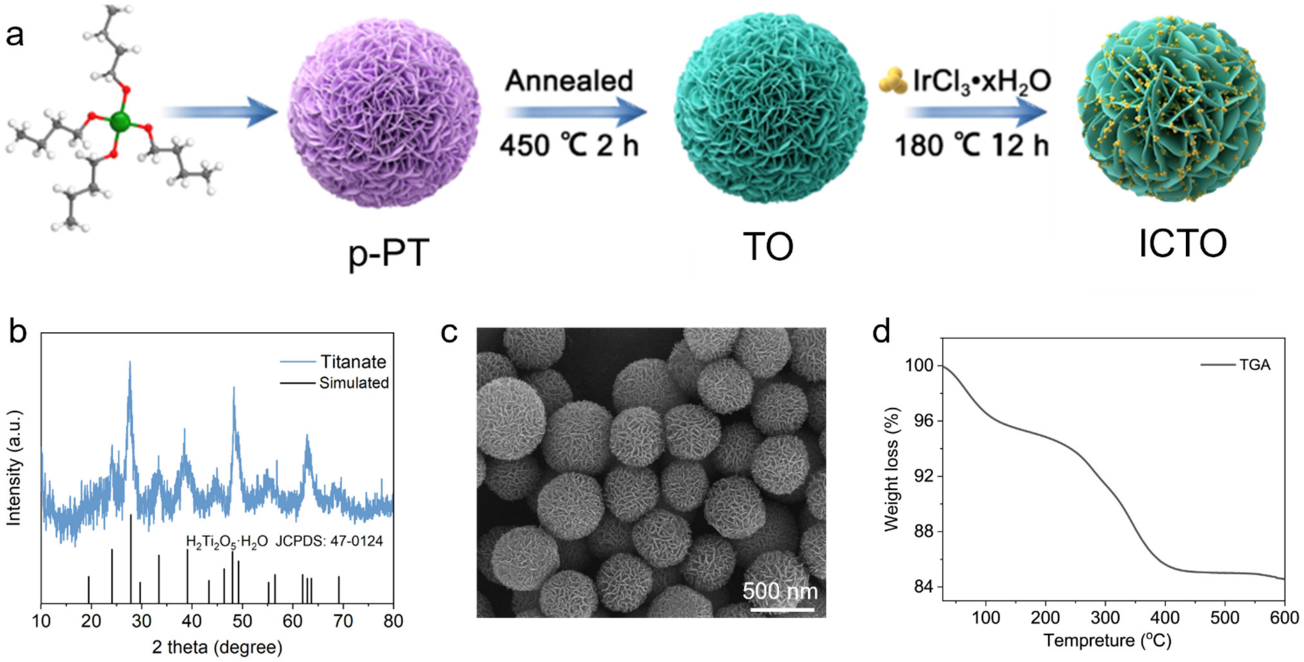

Supplementary Fig. 1. a Schematic illustration of the preparation of Ir cluster decorated TiO_2 (ICTO) with petaloid morphology and wrinkled porous structure. **b** X-ray diffraction (XRD) patterns of petaloid protonated titanate (p-PT). **c** Scanning electron microscopy (SEM) image of p-PT. **d** Thermogravimetric analysis (TGA) and differential scanning calorimetry curves of p-PT in the temperature range of 30-600 °C. Source data are provided as a Source Data file.

The procedures for synthesizing petaloid hierarchical spherical ICTO have been comprehensively detailed in Supplementary Fig. 1a. Initially, well-crystallized petaloid p-PT nanoparticles were successfully fabricated by a solvothermal treatment (Supplementary Fig. 1b). Subsequently, scanning electron microscopy (SEM) images expose an open-up architecture with a nanosheet surface structure in the obtained samples (Supplementary Fig. 1c). The conversion of p-PT particles into TO was achieved through pyrolysis in a controlled air atmosphere. Weight tracking using thermogravimetric analysis reveals a substantial weight loss between 150 °C and 350 °C, demonstrating the extinction of the organic component and the transition of p-PT (Supplementary Fig. 1d). Ultimately, the TO nanospheres were amalgamated with $IrCl_3$ in an ethanol solution, which underwent a solvothermal reaction after electrostatic interaction to create the ICTO biocatalysts.

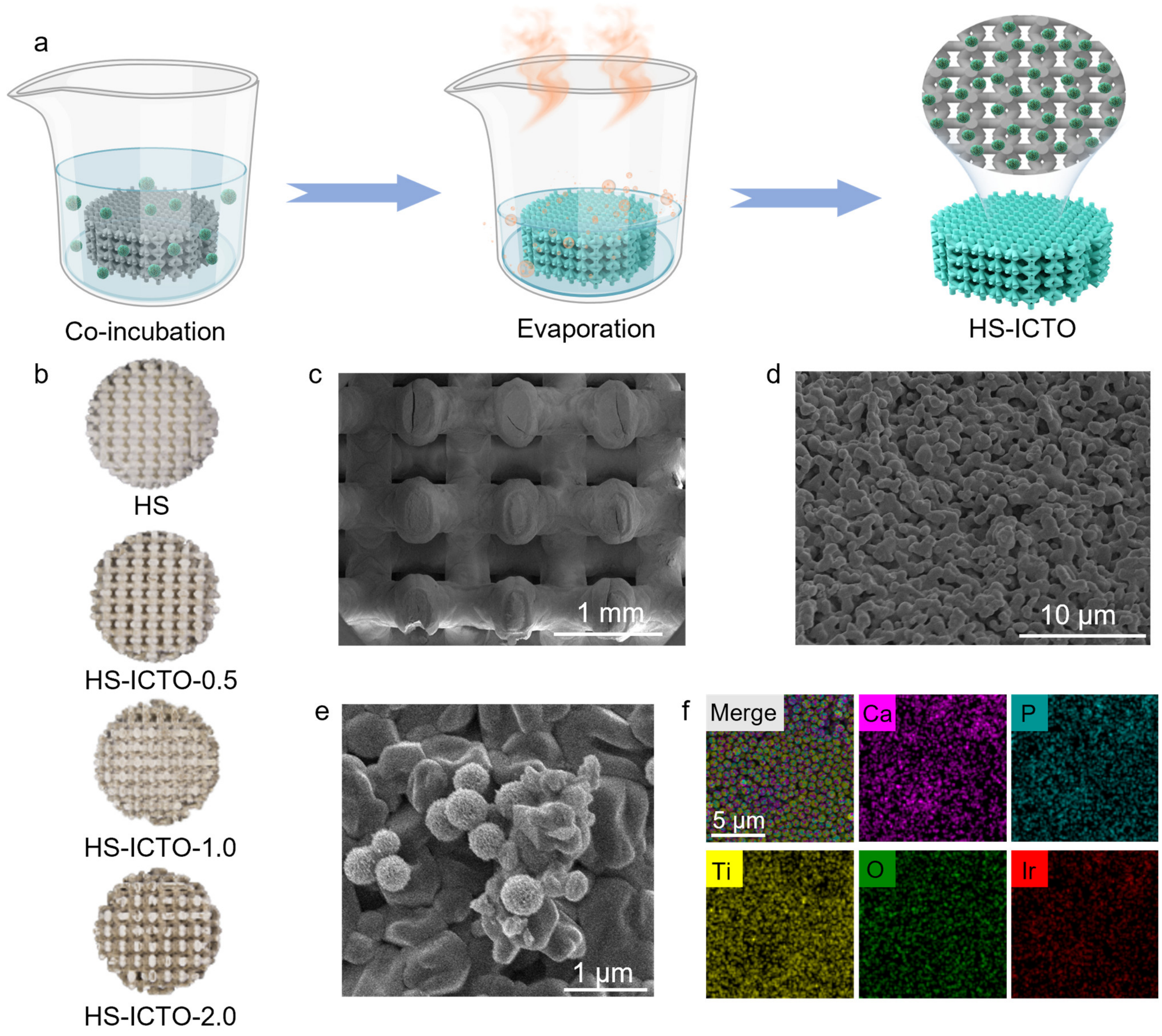

Supplementary Fig. 2. a Schematic illustration of the fabrication procedure of the ICTO-loaded 3D-printed hydroxyapatite scaffold (HS-ICTO). **b** Digital photograph image of HS-ICTO-x (x=0, 0.5, 1.0, 2.0 mg/mL of ICTO aqueous dispersion). **c** Field-emission SEM (FESEM) image of blank HS. **d** Enlarged FESEM image of blank HS. **e** Enlarged FESEM image of HS-ICTO. **f** Energy-dispersive X-ray spectroscopy (EDS) elemental mapping for HS-ICTO.

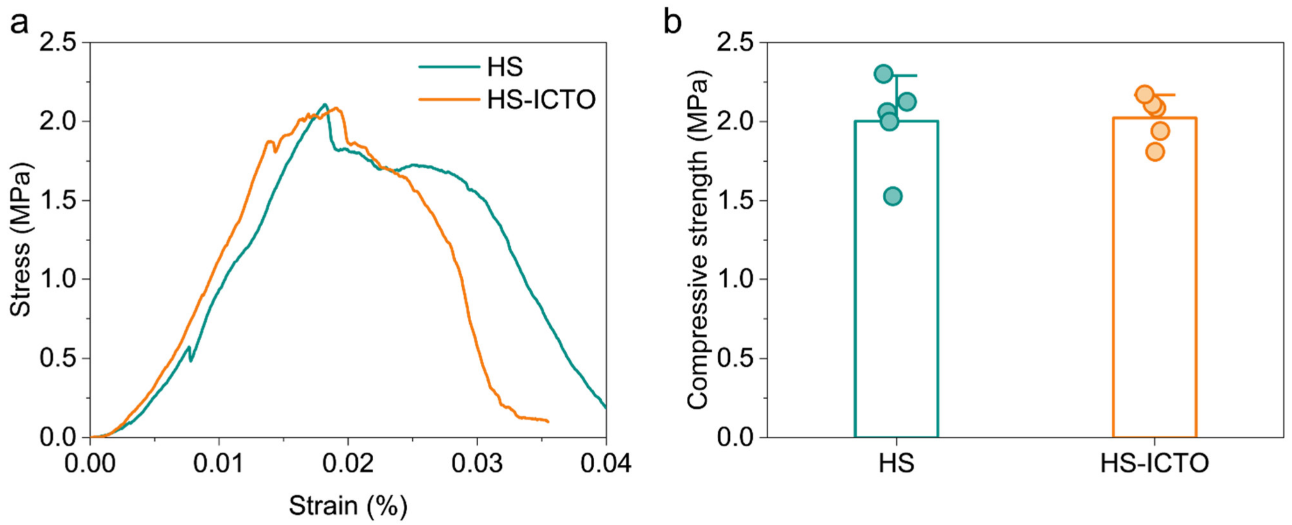

Supplementary Fig. 3. a Stress-strain curves and **b** Compressive strength test of HS and HS-ICTO (n = 5 independent experiments, data are presented as mean ± SD). Source data are provided as a Source Data file.

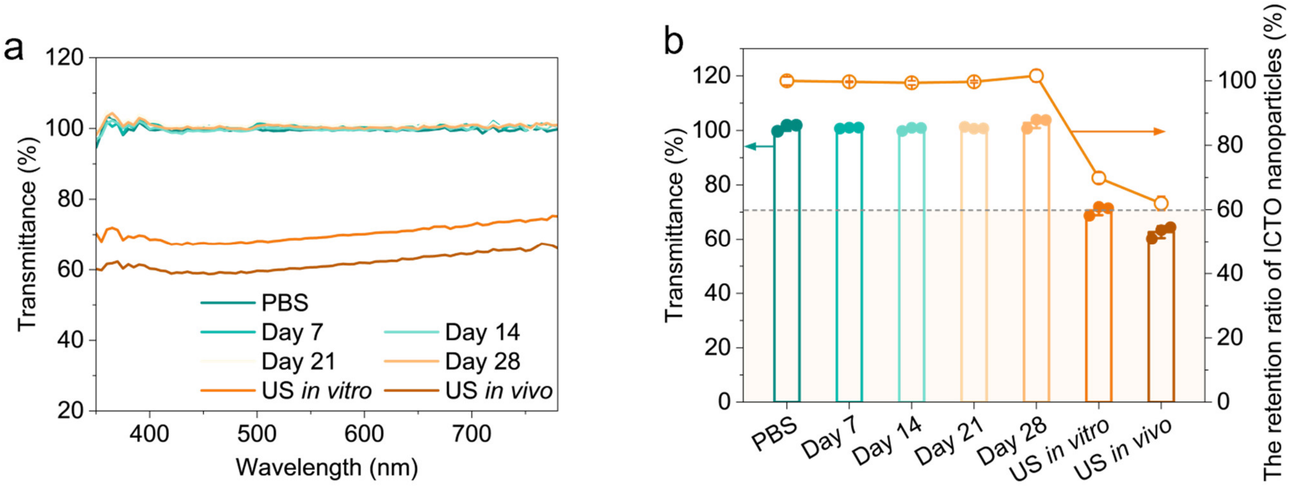

Supplementary Fig. 4. a Transmittance spectra of solutions with different immersion times in the visible wavelength range. **b** Quantitative analysis of transmittance in the immersion solution across the 350-600 nm wavelength range and retention rate of ICTO nanoparticles on the scaffold (n = 3 independent experiments, data are presented as mean ± SD; US *in vitro*: 1.0 W/cm^2, 1 MHz, 30% duty cycle, 5 min; US *in vivo*: 2.5 W/cm^2, 1 MHz, 30% duty cycle, 5 min). Source data are provided as a Source Data file.

To elucidate the robust interfacial interaction between ICTO nanoparticles and HS within the fabricated HS-ICTO scaffold, we subjected the scaffold to prolonged immersion in phosphate-buffered saline (PBS) to simulate the physiological microenvironment. The experimental results reveal that

ICTO nanoparticles maintain stable adhesion to the surface of the HS scaffold throughout the extended immersion period, with negligible reduction in the transmittance of the immersion solution. Upon sonication-induced mechanical stress, while some particle detachment occurred, over 60% of ICTO nanoparticles remain anchored to the scaffold, validating the durability of the fabricated HS-ICTO scaffolds.

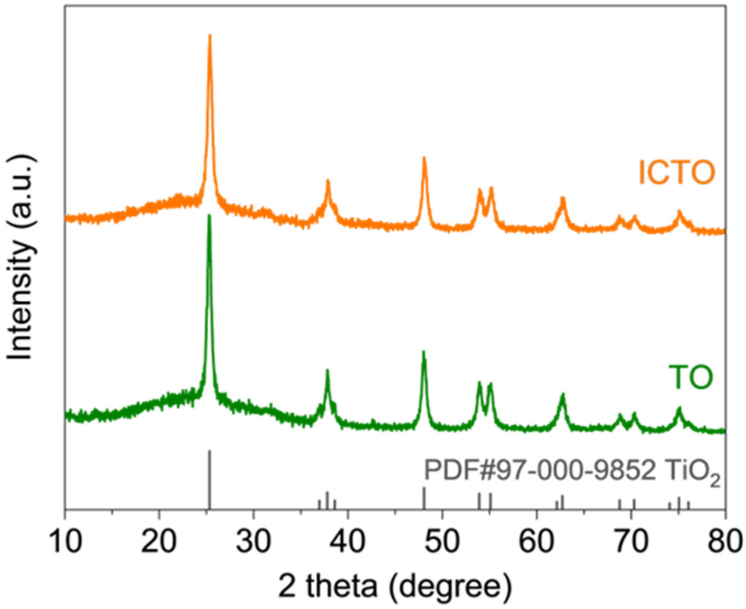

Supplementary Fig. 5. XRD patterns of TiO_2 (TO) and ICTO. Source data are provided as a Source Data file.

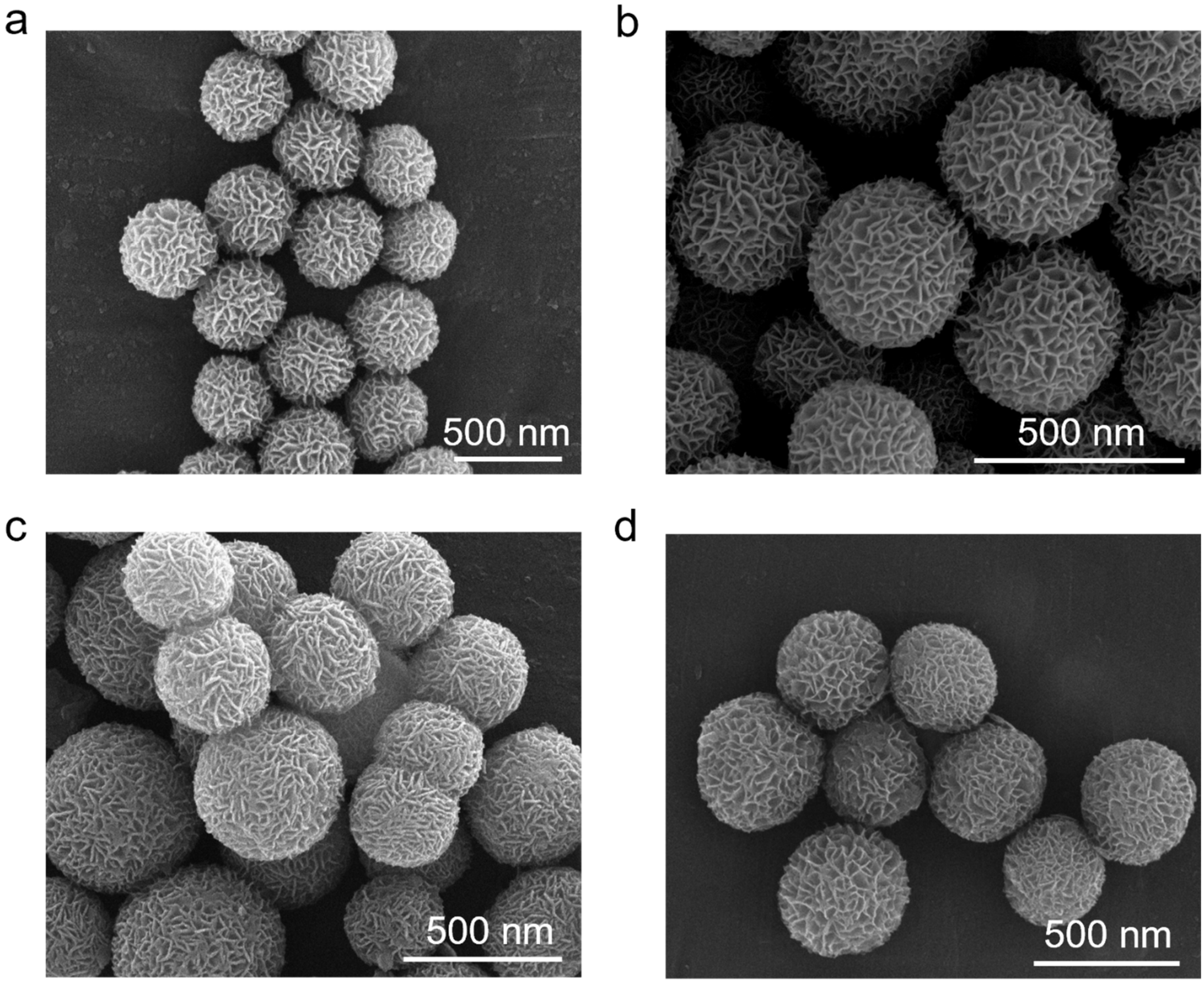

Supplementary Fig. 6. a, b SEM images of TO and **c, d** ICTO.

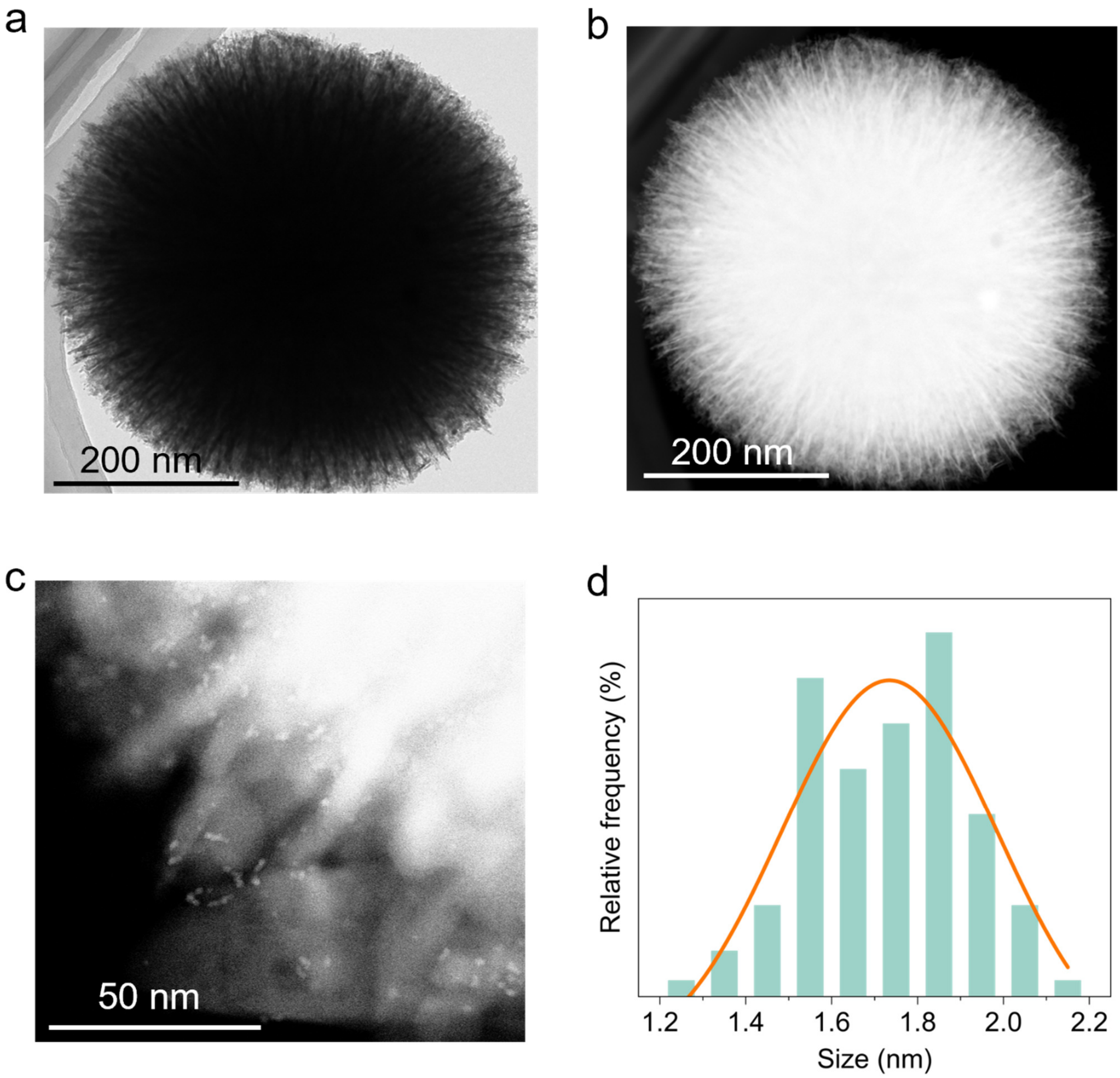

Supplementary Fig. 7. **a** Bright-field transmission electron microscopy (TEM) image of ICTO. **b** Dark-field TEM image of ICTO. **c** Enlarged TEM image of ICTO. **d** The corresponding average particle sizes of Ir clusters in ICTO. Source data are provided as a Source Data file.

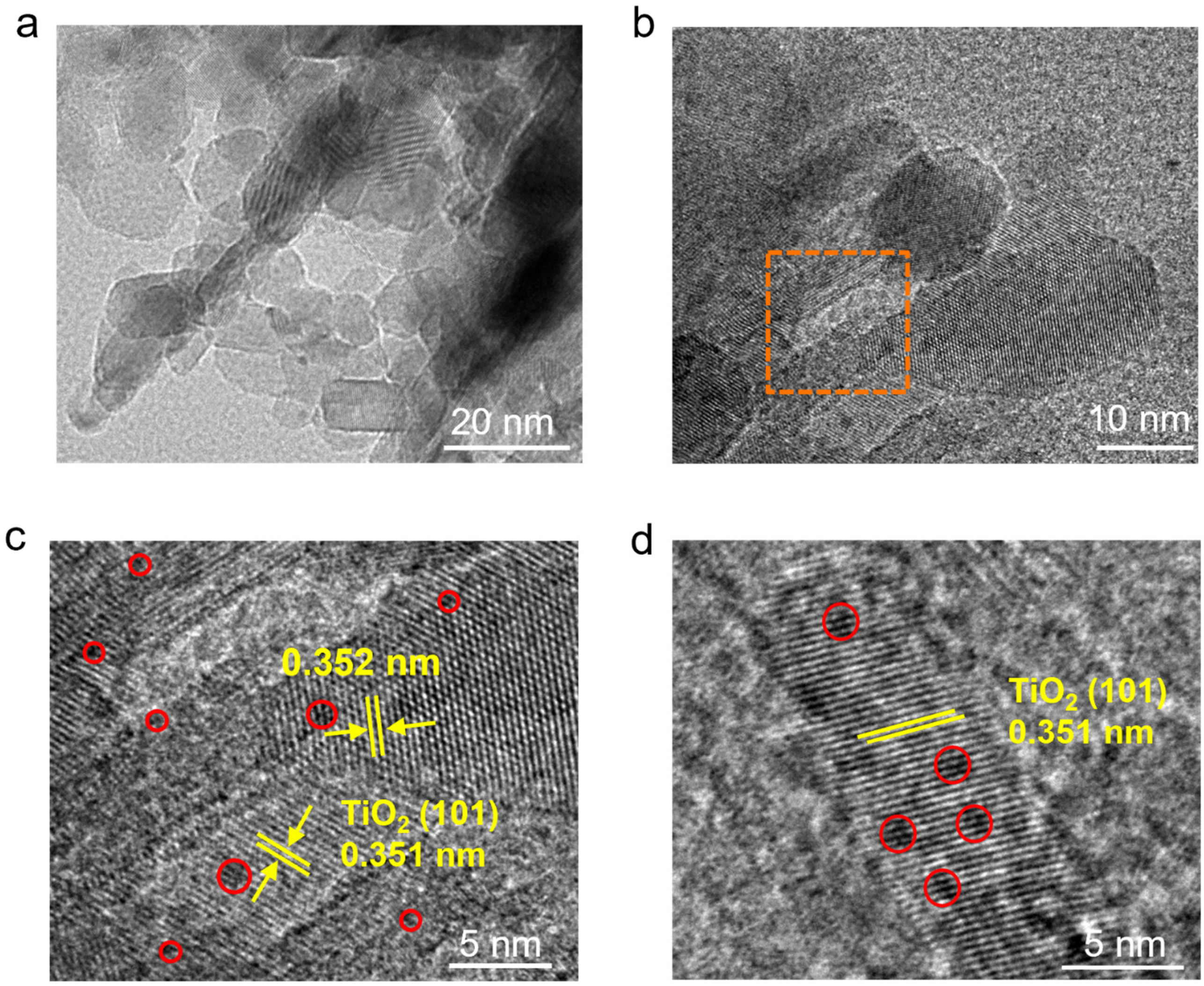

Supplementary Fig. 8. High-resolution TEM images of ICTO.

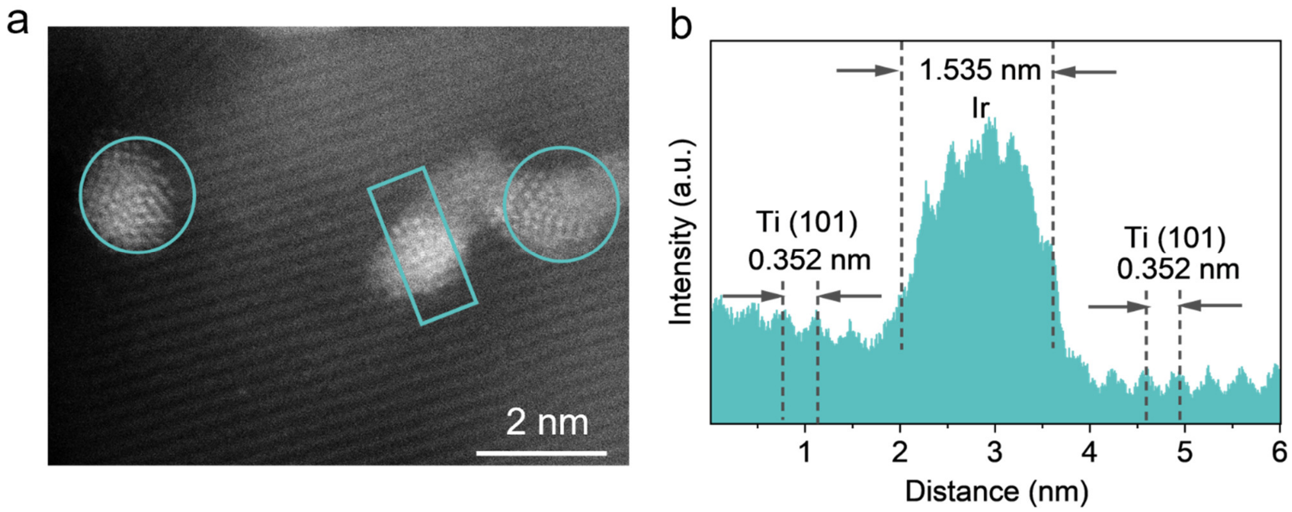

Supplementary Fig. 9. High-angle annular dark-field scanning TEM (HAADF-STEM) image of ICTO. Source data are provided as a Source Data file.

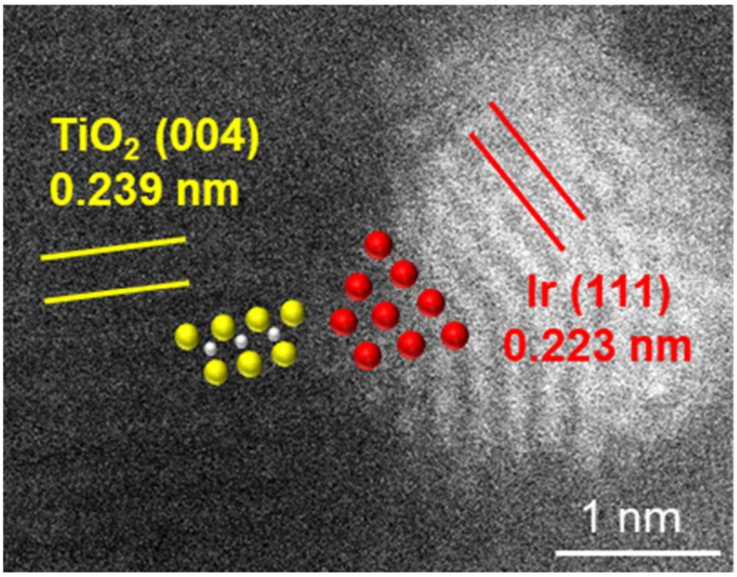

Supplementary Fig. 10. A schematic atomic structure of ICTO is superposed on the HAADF–STEM image.

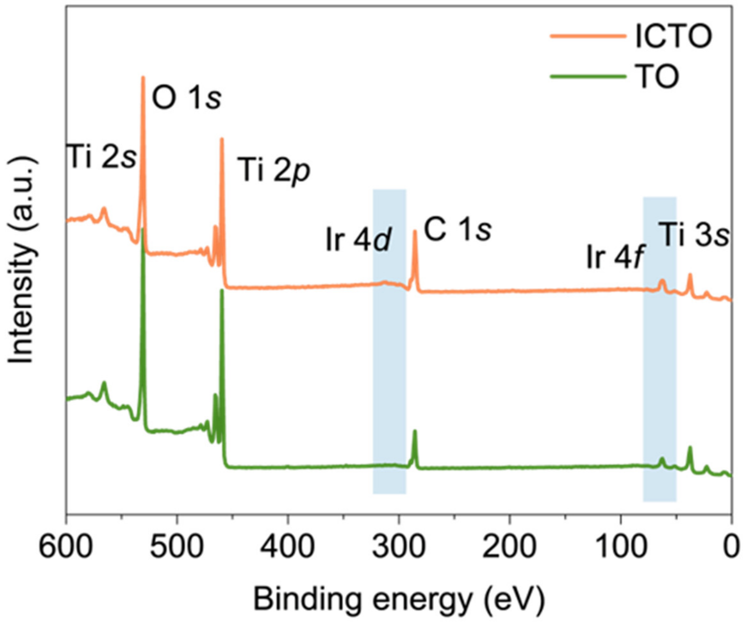

Supplementary Fig. 11. X-ray photoelectron spectroscopy (XPS) survey of ICTO and TO. Source data are provided as a Source Data file.

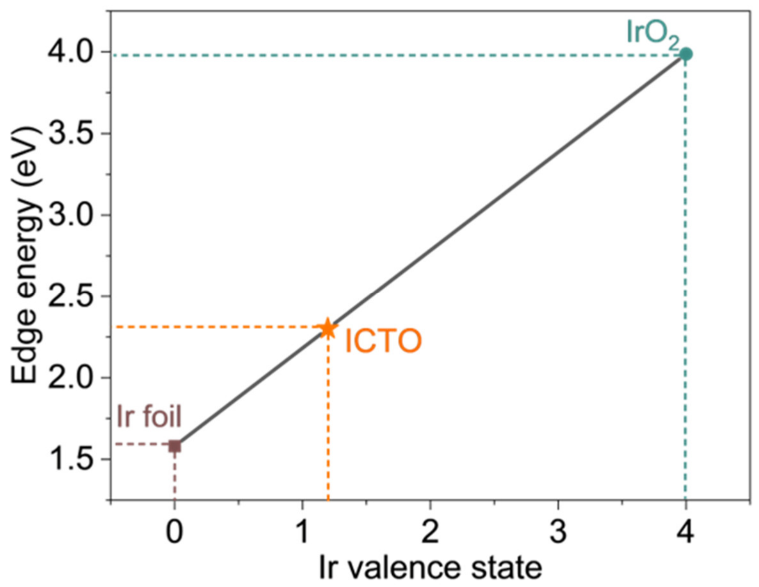

Supplementary Fig. 12. Valence analysis of Ir species in ICTO. Source data are provided as a Source Data file.

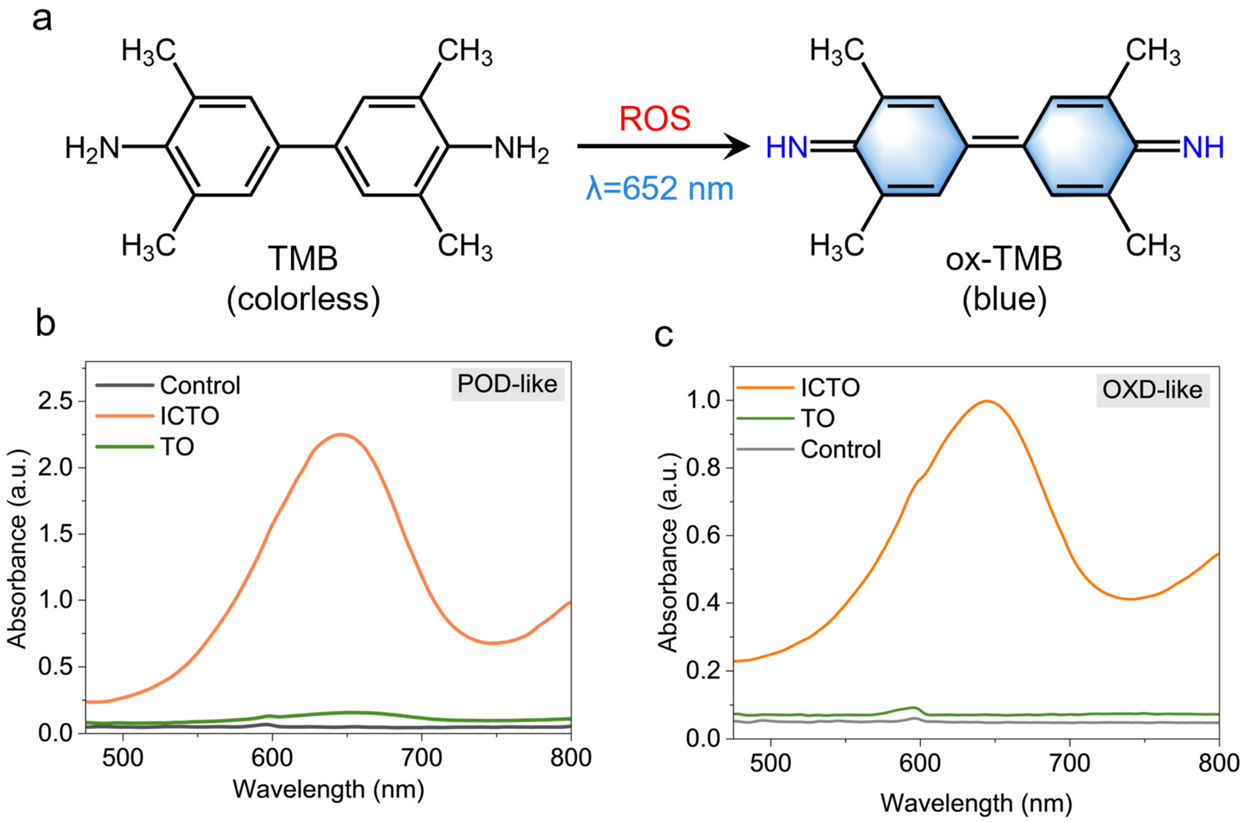

Supplementary Fig. 13. a ROS catalytic oxidation reaction of 3,3′,5,5′-tetramethylbenzidine (TMB). **b, c** UV–vis absorption spectra of oxidized TMB (ox-TMB) incubated with ICTO and TO. Source data are provided as a Source Data file.

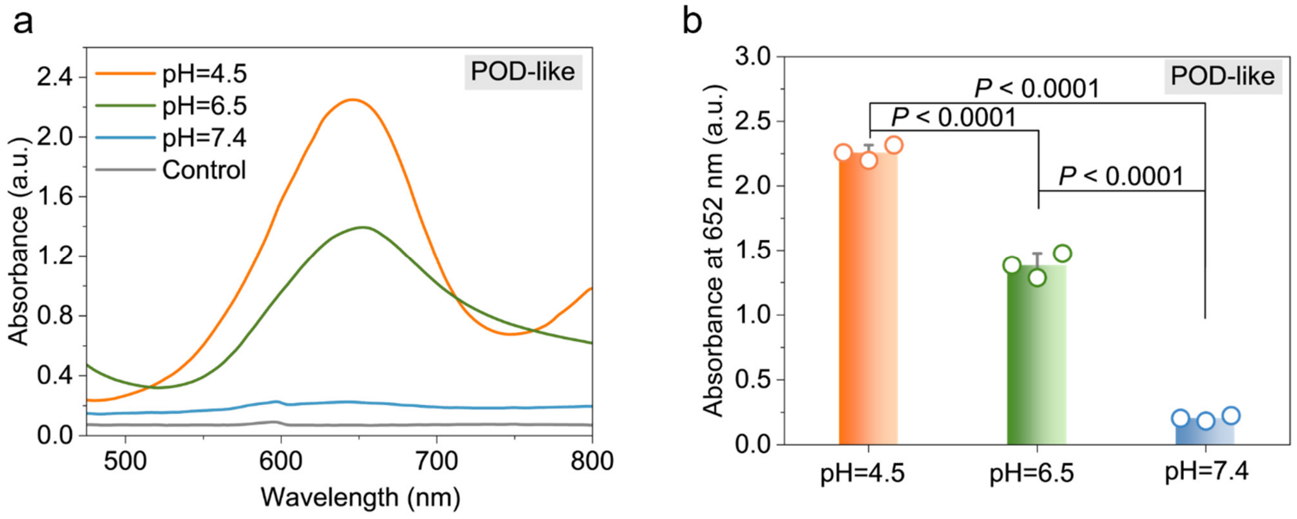

Supplementary Fig. 14. a UV–vis absorption spectra of ox-TMB incubated with ICTO and TO in the presence of H_2O_2. **b** Quantitative analysis of absorbance at λ = 652 nm in the presence of H_2O_2 substrate at different pH conditions (n = 3 independent experiments, data are presented as mean ± SD). Statistical significance was calculated using one-way analysis of variance (ANOVA) followed by Tukey's post-hoc test for multiple comparisons; all tests were two-sided. Source data are provided as a Source Data file.

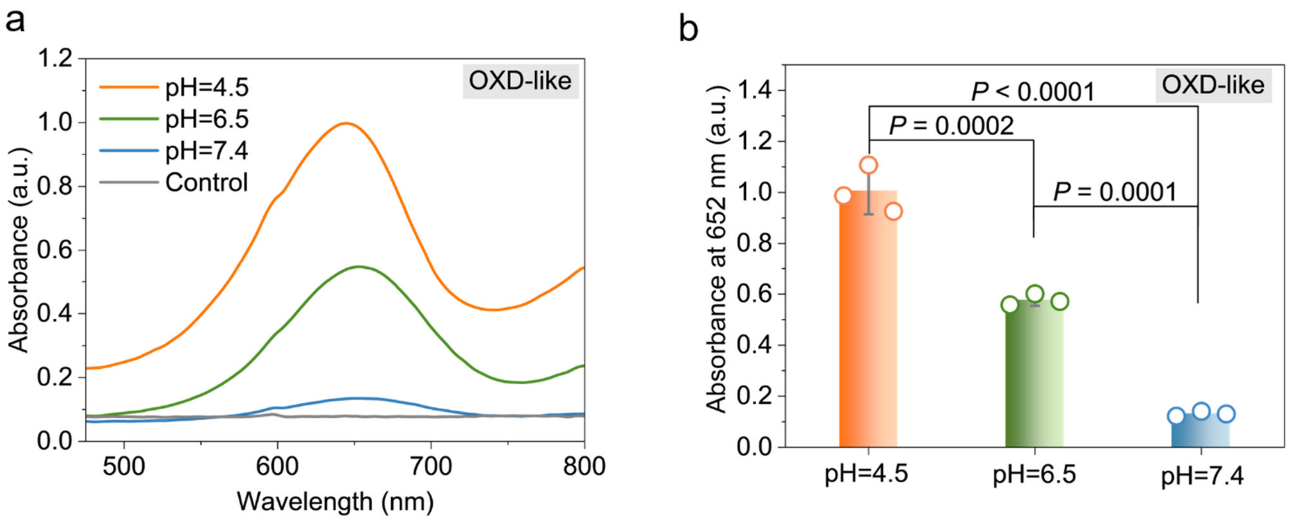

Supplementary Fig. 15. a UV–vis absorption spectra of ox-TMB incubated with ICTO and TO in the absence of H_2O_2. **b** Quantitative analysis of absorbance at λ = 652 nm at different pH conditions (n = 3 independent experiments, data are presented as mean ± SD). Statistical significance was calculated using one-way ANOVA followed by Tukey's post-hoc test for multiple comparisons; all tests were two-sided. Source data are provided as a Source Data file.

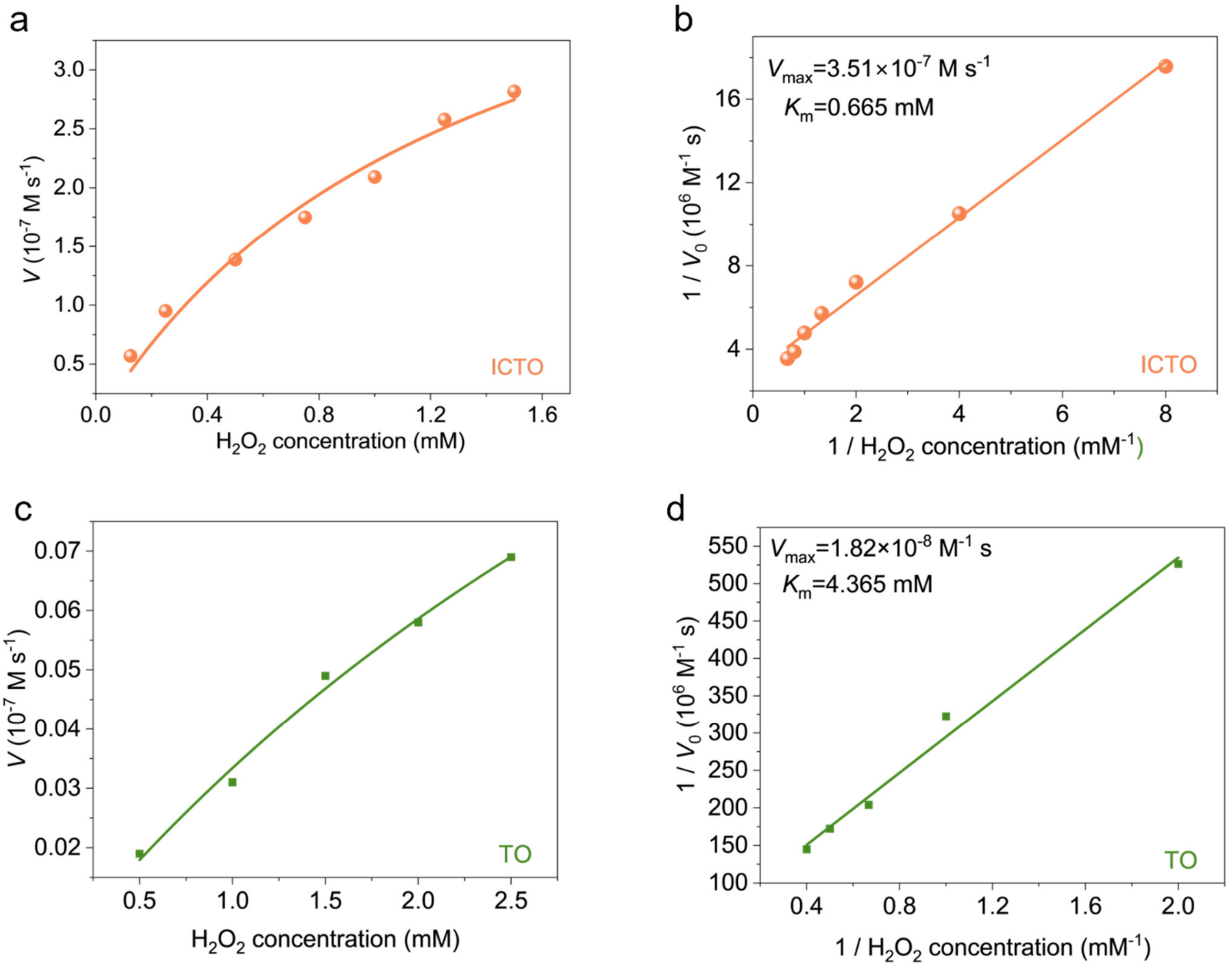

Supplementary Fig. 16. a Typical Michaelis-Menten curves of ICTO with H_2O_2. **b** Double-reciprocal plots for determining the kinetic constants of different concentrations of ICTO with H_2O_2.**c** Typical Michaelis-Menten curves of TO with H_2O_2. **d** Double-reciprocal plots for determining the kinetic constants of different concentrations of TO with H_2O_2. Source data are provided as a Source Data file.

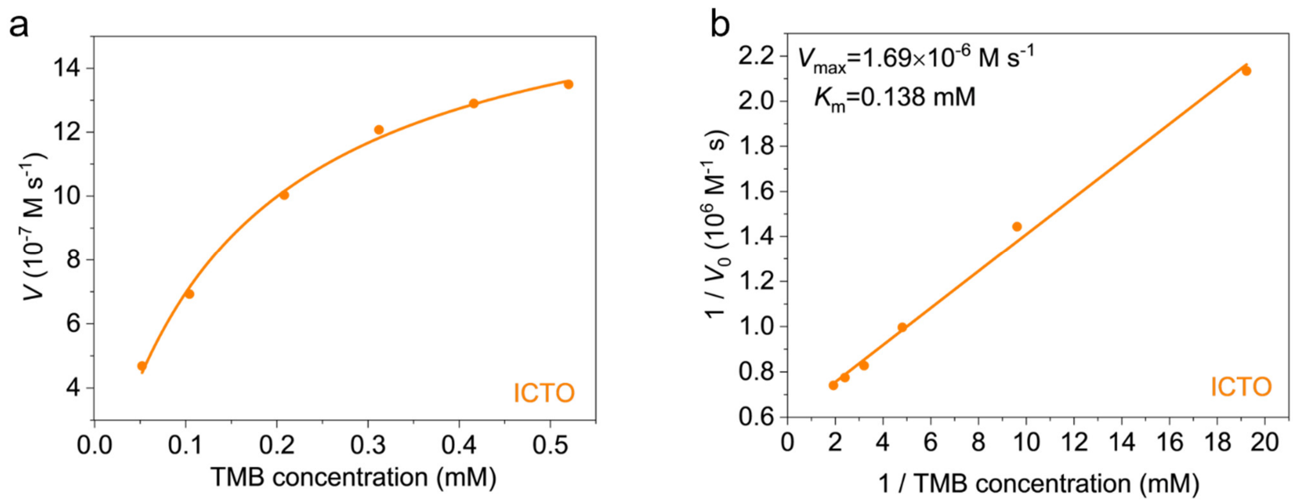

Supplementary Fig. 17. a Typical Michaelis-Menten curves of ICTO with TMB. **b** Double-reciprocal plots for determining the kinetic constants of different concentrations of TMB. Source data are provided as a Source Data file.

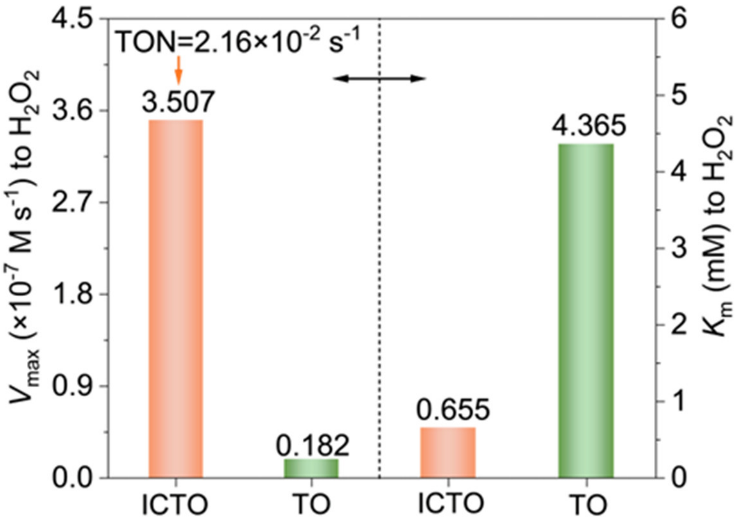

Supplementary Fig. 18. V_{max} and K_m values of ICTO and TO with H_2O_2. Source data are provided as a Source Data file.

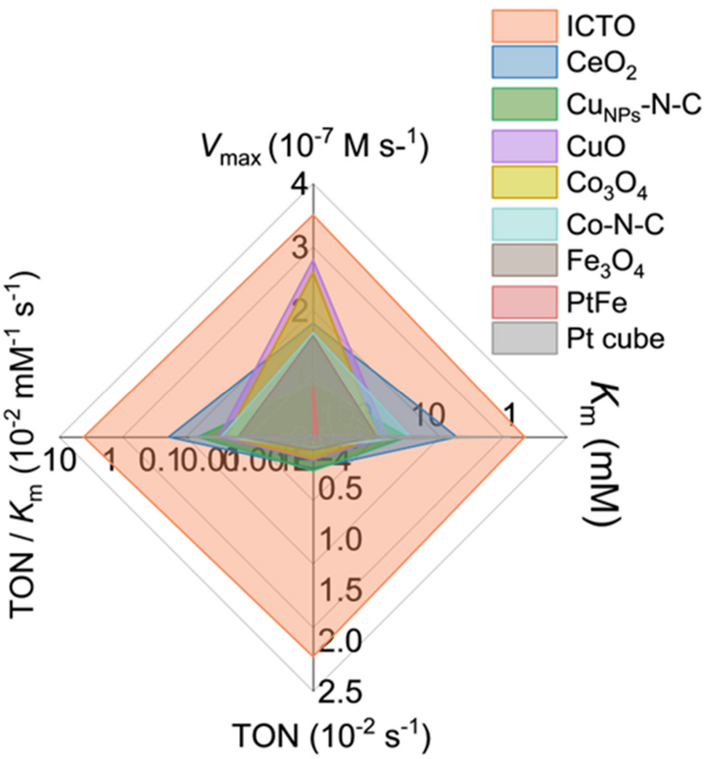

Supplementary Fig. 19. ROS-related catalytic performance index comparison of this study and earlier reported POD-mimics. Source data are provided as a Source Data file.

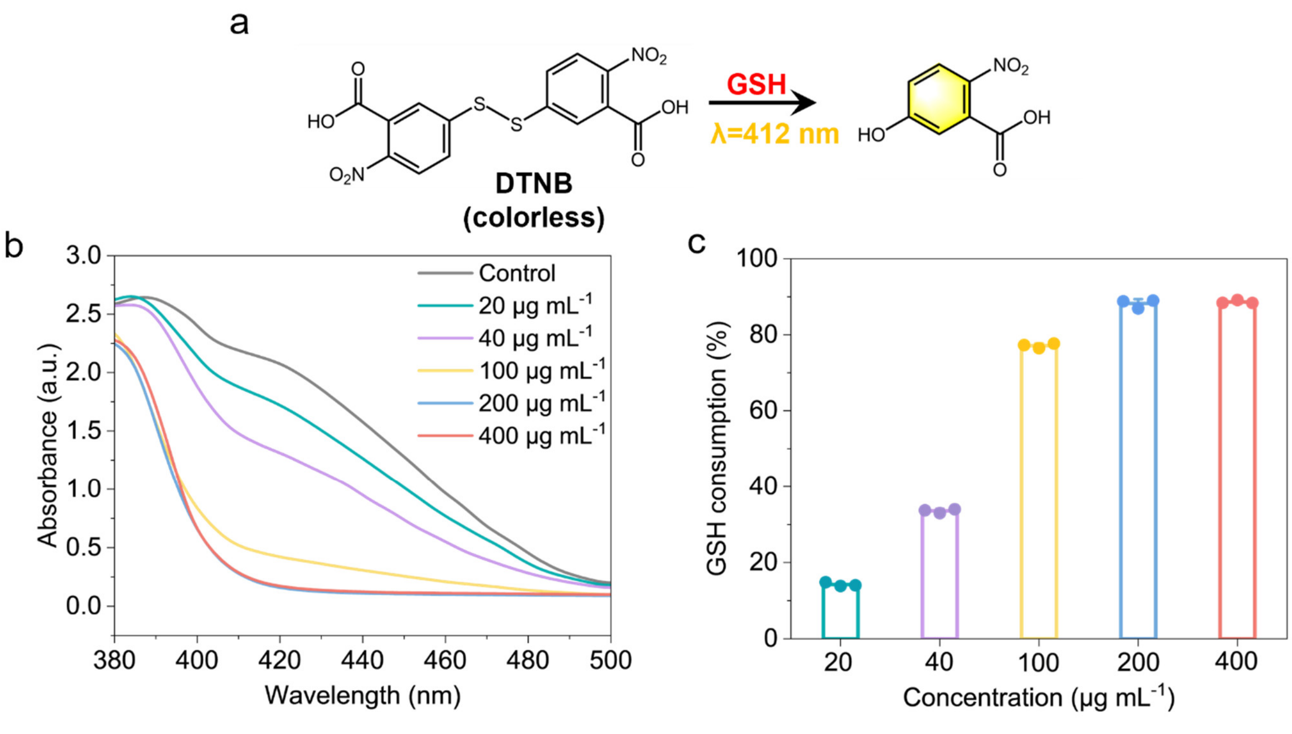

Supplementary Fig. 20. a Mechanism of the chromogenic reaction using DTNB as a trapping agent for -SH of glutathione. **b**, **c** GSH consumption capacity after incubation with different material

concentrations (n = 3 independent experiments, data are presented as mean ± SD). Source data are provided as a Source Data file.

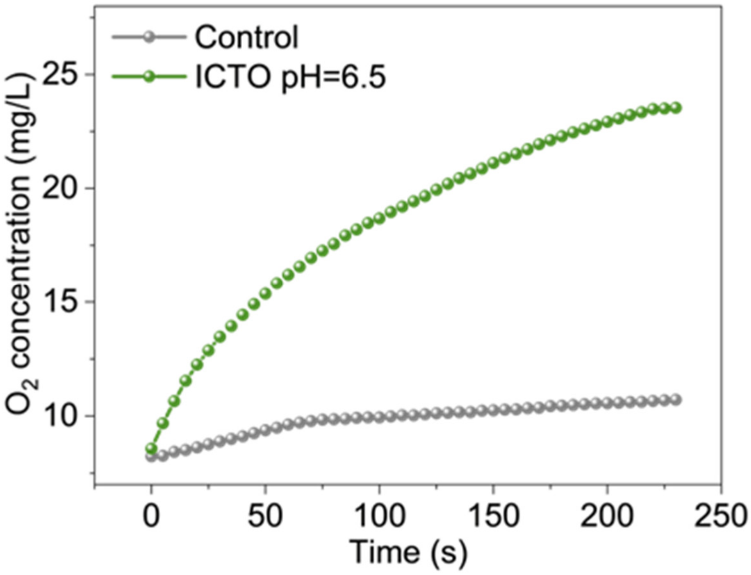

Supplementary Fig. 21. Real-time O_2 generation curves in the presence of H_2O_2 at pH 6.5. Source data are provided as a Source Data file.

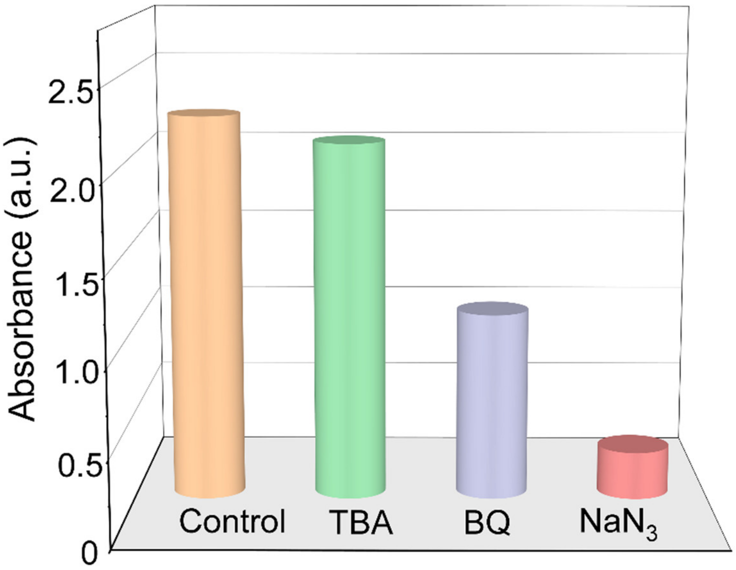

Supplementary Fig. 22. The free radical quenching experiments, tert-butyl alcohol (TBA), p-benzoquinone (BQ), and sodium azide (NaN_3) were utilized as quenching agents for •OH, •O_2^-, and $^1O_2$, respectively. Source data are provided as a Source Data file.

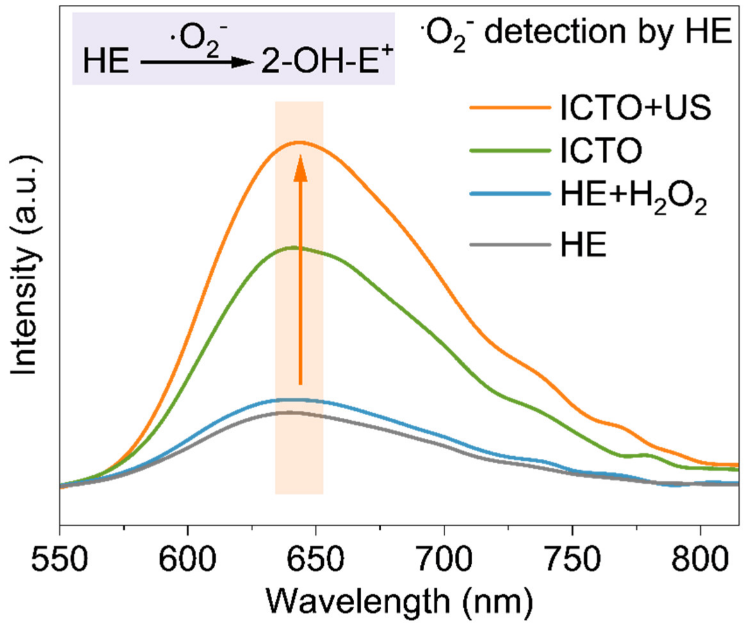

Supplementary Fig. 23. The generation of $\bullet O_2^-$ and sonication enhancement was confirmed by the HE probe (US: 2.0 W/cm^2, 1 MHz, 30% duty cycle). Source data are provided as a Source Data file.

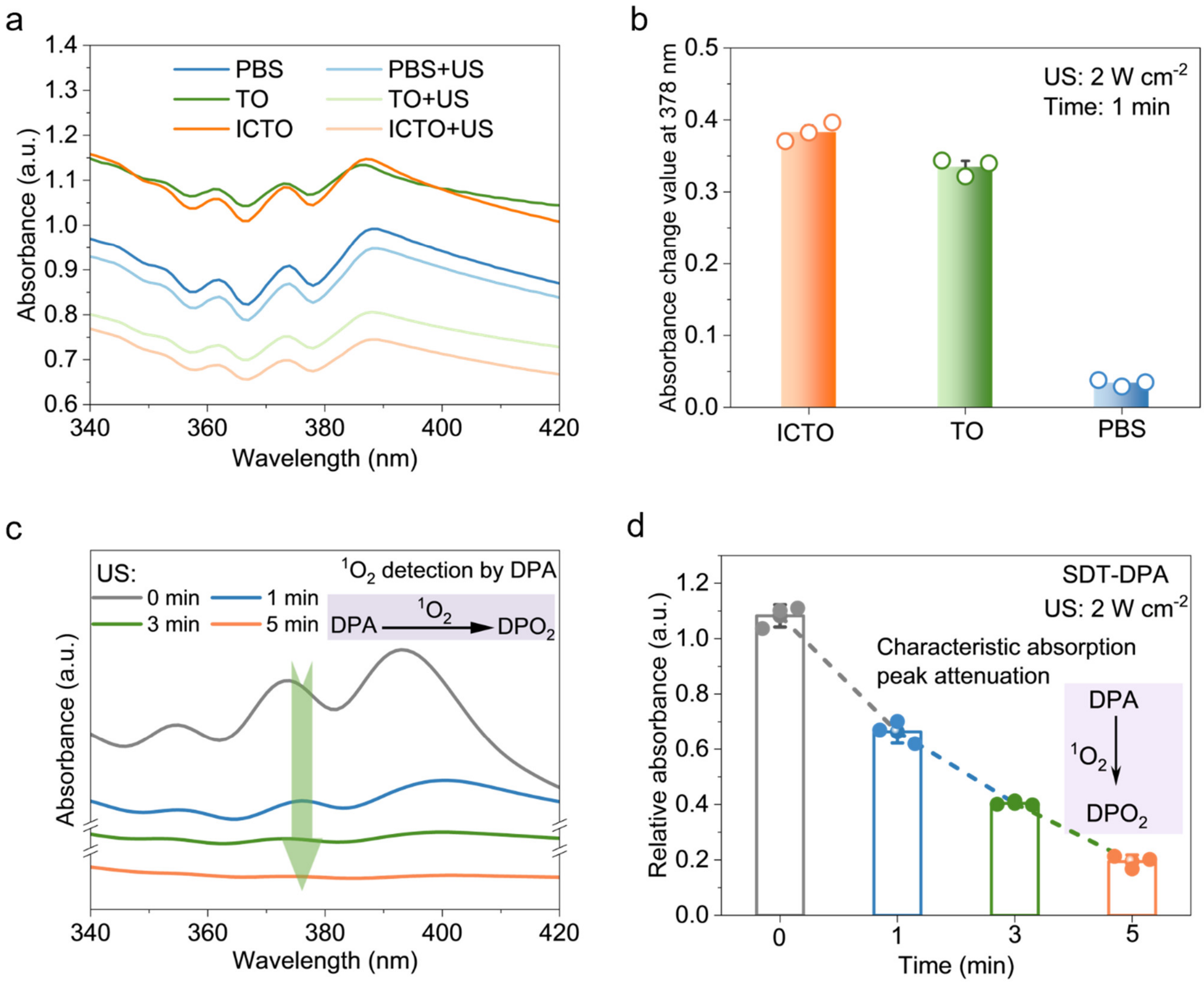

Supplementary Fig. 24. a, **b** Sono-stimulated catalytic oxidation of DPA in different samples (2.0 W/cm^2, 1 MHz, 30% duty cycle) (n = 3 independent experiments, data are presented as mean ± SD). **c**, **d** Catalytic oxidation of DPA by ICTO at different sonication times (n = 3 independent experiments, data are presented as mean ± SD). Source data are provided as a Source Data file.

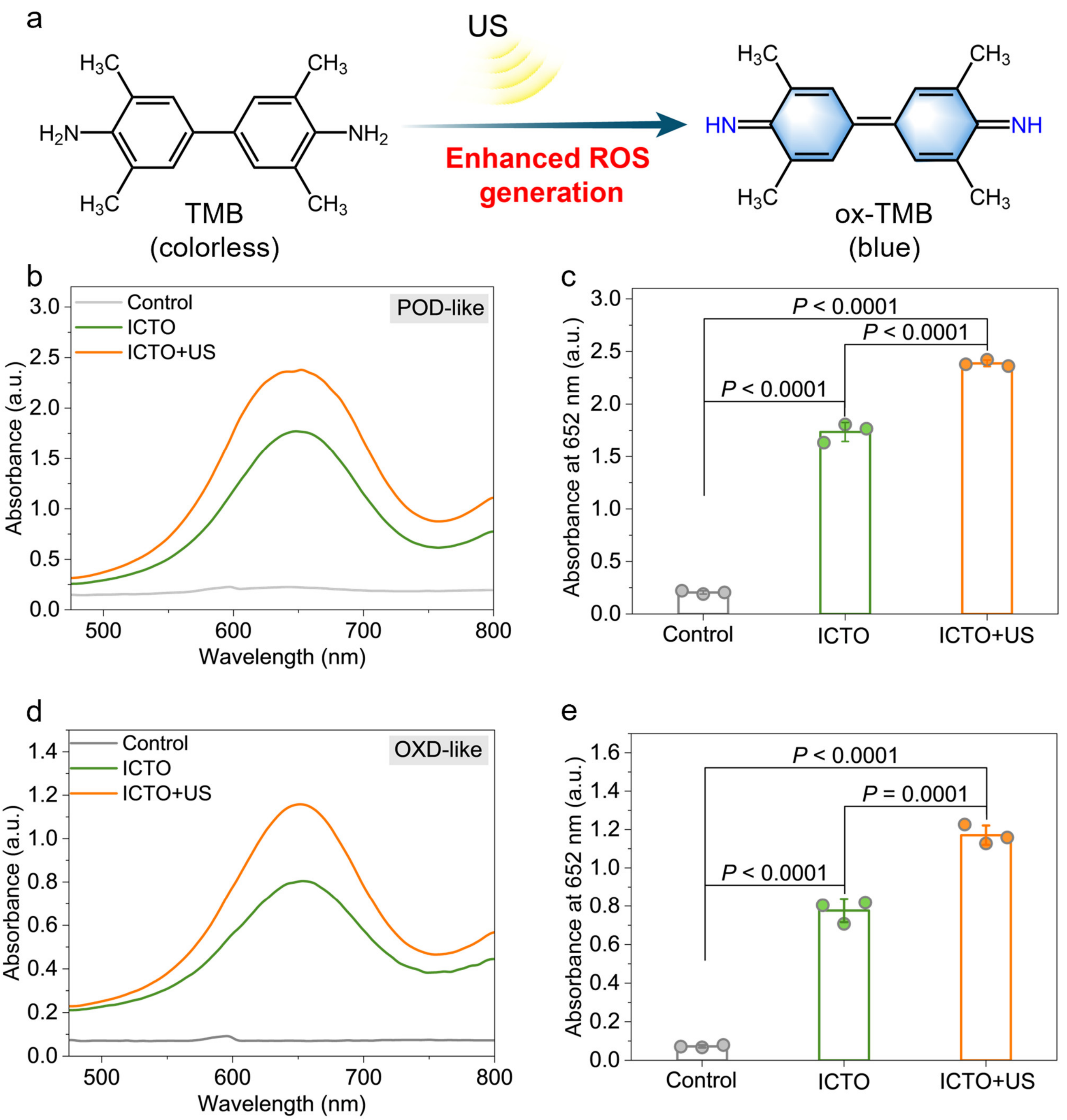

Supplementary Fig. 25. Enhanced POD-like and OXD-like activities of ICTO induced by additional US (2.0 W/cm^2, 1 MHz, 30% duty cycle; n = 3 independent experiments, data are presented as mean ± SD). Statistical significance was calculated using one-way ANOVA followed by Tukey's post-hoc test for multiple comparisons; all tests were two-sided. Source data are provided as a Source Data file.

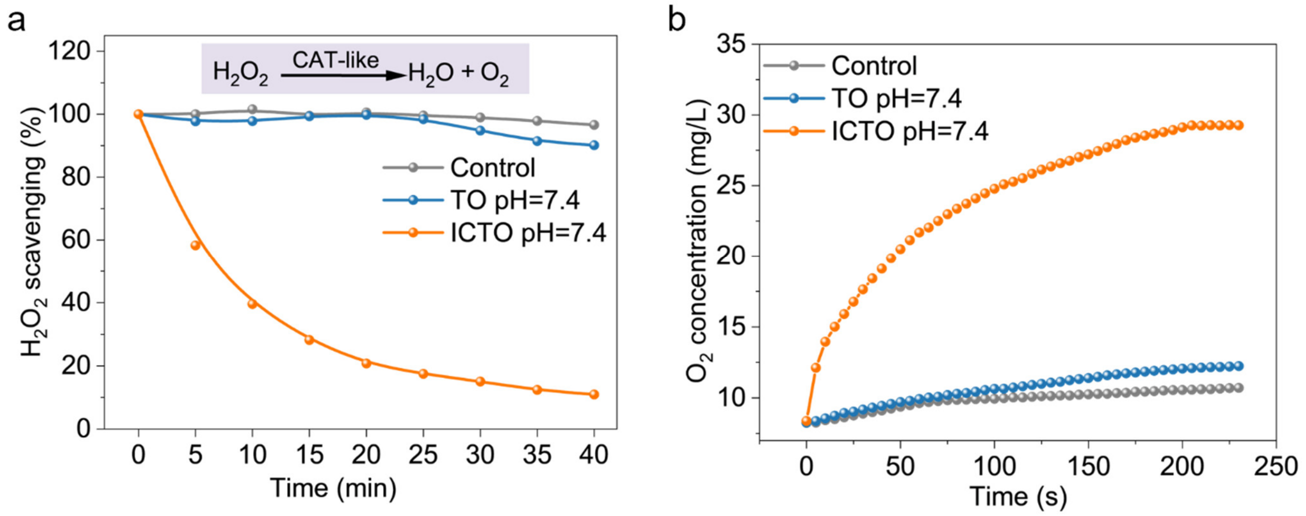

Supplementary Fig. 26. a The dynamic H_2O_2 scavenging capacity of ICTO and TO at pH 7.4. **b** Real-time O_2 generation curves in the presence of H_2O_2 at pH 7.4. Source data are provided as a Source Data file.

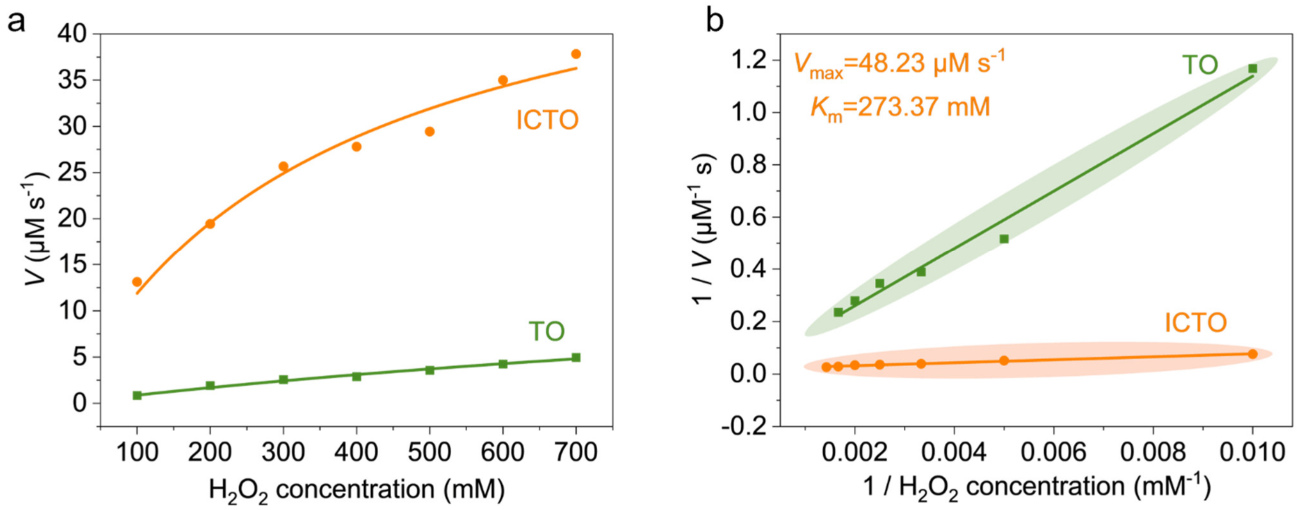

Supplementary Fig. 27. a Typical Michaelis-Menten curves of TO and ICTO. **b** Lineweaver–Burk plotting for TO and ICTO with H_2O_2 as substrate. Source data are provided as a Source Data file.

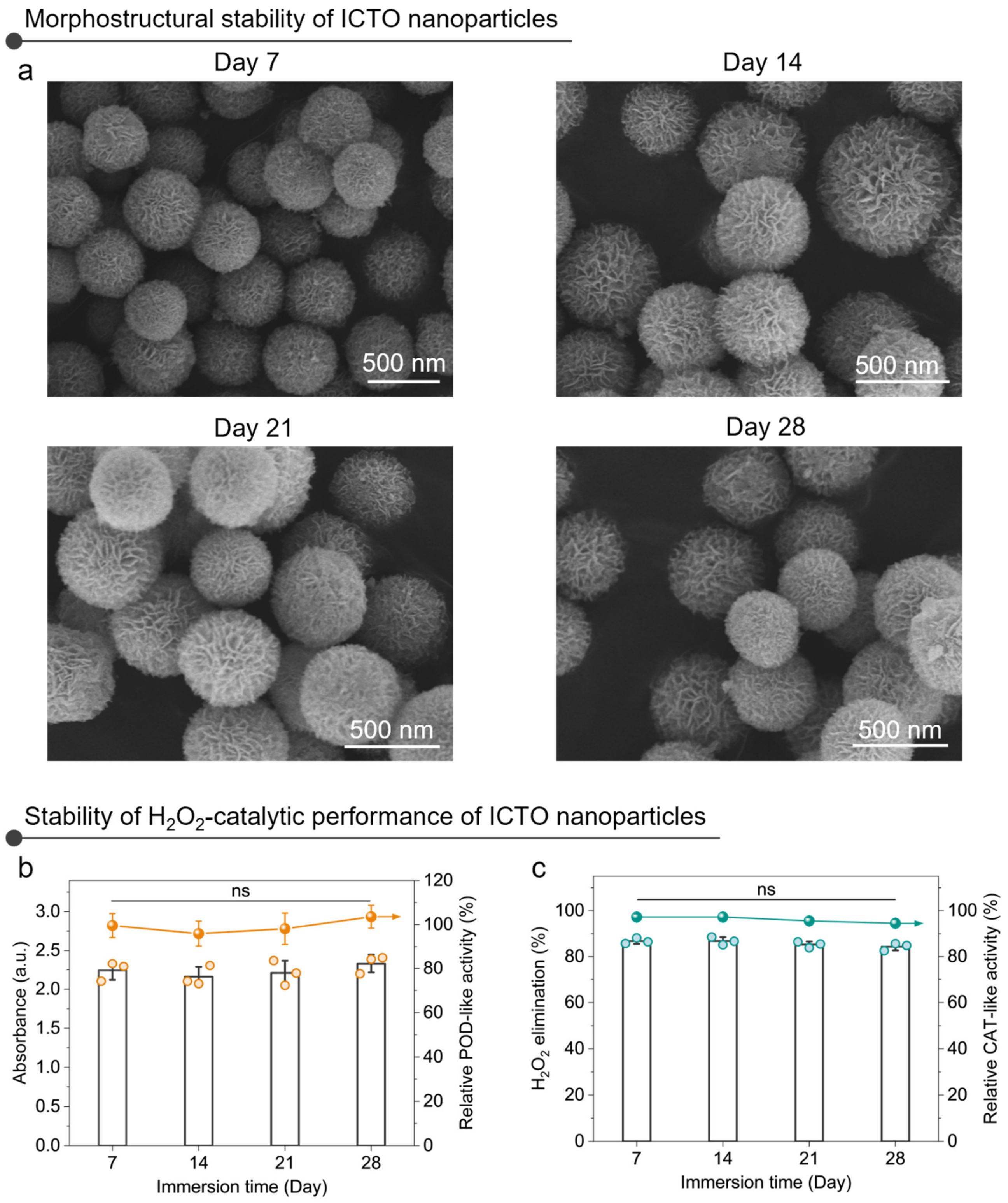

Supplementary Fig. 28. The morphological structure and H_2O_2-catalytic performance stability of ICTO nanoparticles were determined at different immersion time points. **a** SEM images of ICTO following the 7, 14, 21, and 28-day immersion period. **b** POD-like activity (n = 3 independent experiments). **c** CAT-like activity (n = 3 independent experiments). Data are presented as mean ± SD,

and ns represents no significant difference; statistical significance was calculated using one-way ANOVA followed by Tukey's post-hoc test for multiple comparisons; all tests were two-sided. Source data are provided as a Source Data file.

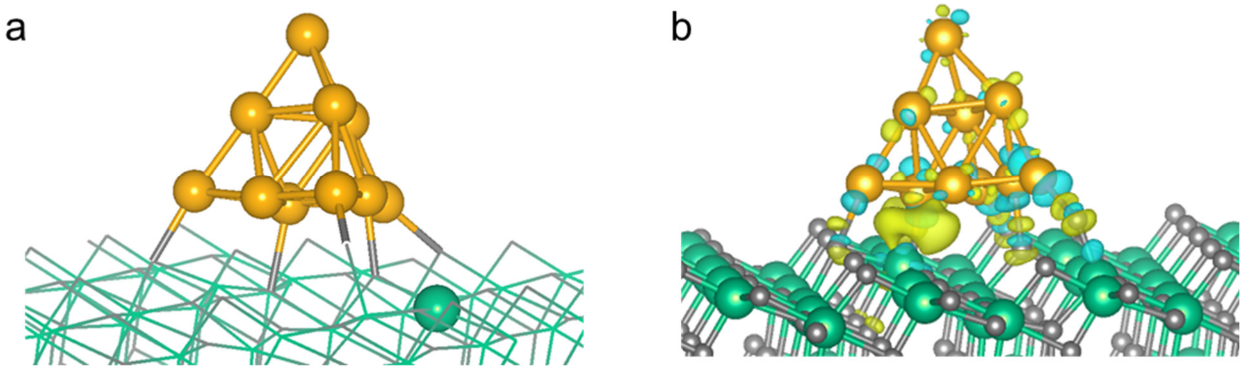

Supplementary Fig. 29. a The model structure of ICTO. **b** The difference charge density analysis of ICTO (color codes: gray, O; green, Ti; orange, Ir; cyan and yellow are employed to denote charge depletion and accumulation, respectively; the cut-off of the density-difference isosurface is 0.01 e·Bohr^{-3}).

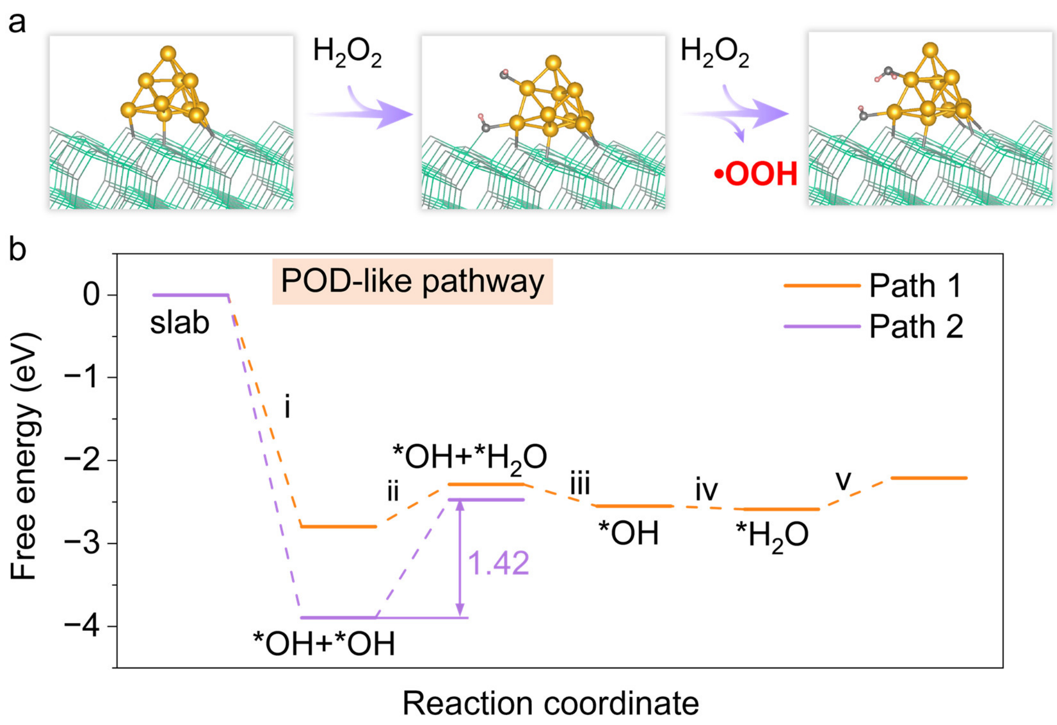

Supplementary Fig. 30. a Another proposed reaction pathways and **b** corresponding Gibbs free energy diagram of POD-like pathways on ICTO (color codes: gray, O; green, Ti; orange, Ir; red, H). Source data are provided as a Source Data file.

In another POD-like alternative pathway, two *OH forms from the self-decomposition of adsorbed H_2O_2, serving as reaction sites on two neighboring Ir atoms. Experimental results demonstrated that during the process of adsorbing the second H_2O_2 and then reacting to release •OOH, the energy barrier is as high as 1.42 eV.

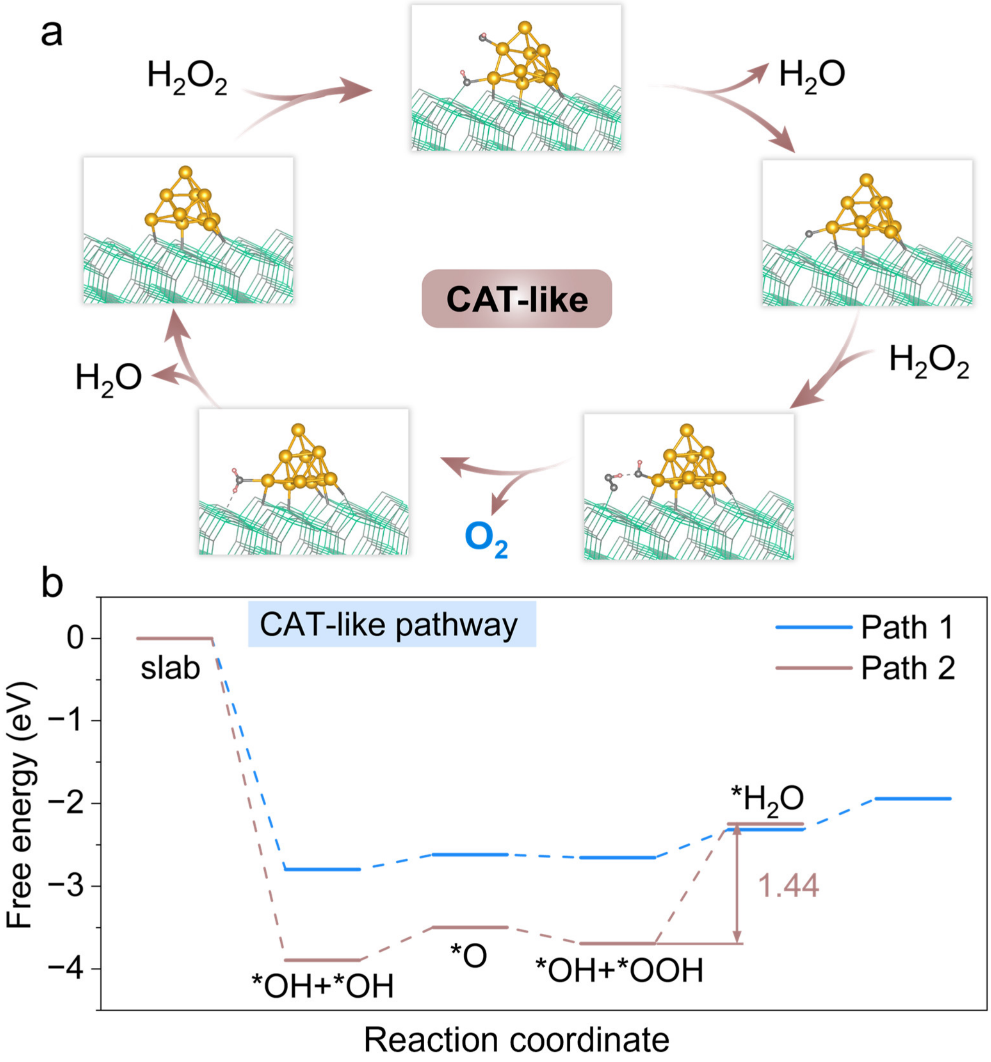

Supplementary Fig. 31. a Another proposed reaction pathways and **b** corresponding Gibbs free energy diagram of CAT-like pathways on ICTO (color codes: gray, O; green, Ti; orange, Ir; red, H). Source data are provided as a Source Data file.

In another CAT-like alternative pathway, two *OH forms from the self-decomposition of adsorbed H_2O_2, serving as reaction sites on two neighboring Ir atoms. Experimental results demonstrated that the desorption of *O_2 is the rate-determining step for this path, wherein the energy barrier is 1.44 eV.

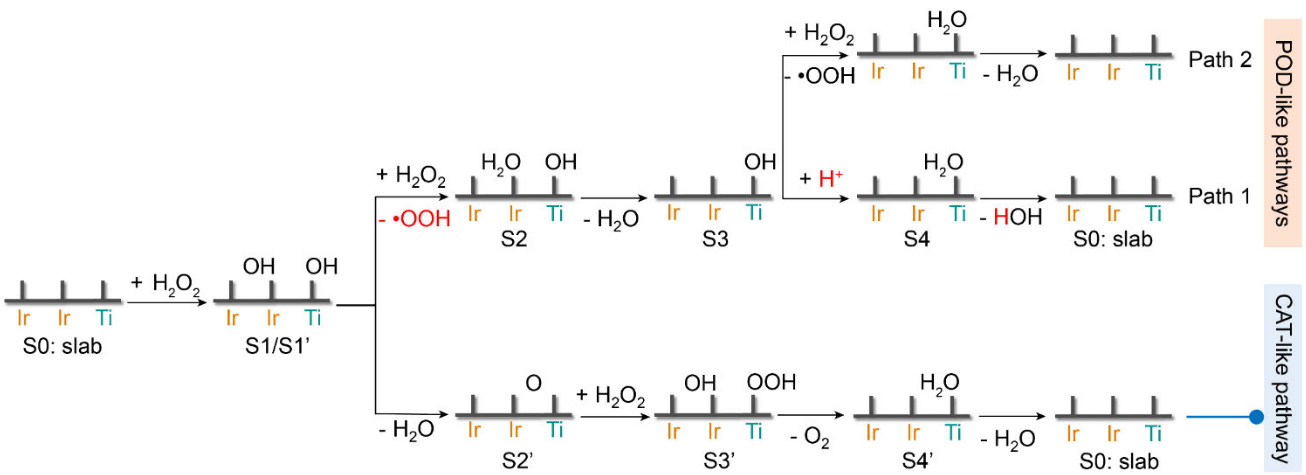

Supplementary Fig. 32. Proposed reaction POD-like and CAT-like pathways on ICTO. Path 2 in POD-like mechanisms: H^+ protons involvement; Path 2 in POD-like mechanisms: reaction with another H_2O_2 to form *H_2O and *OOH intermediates.

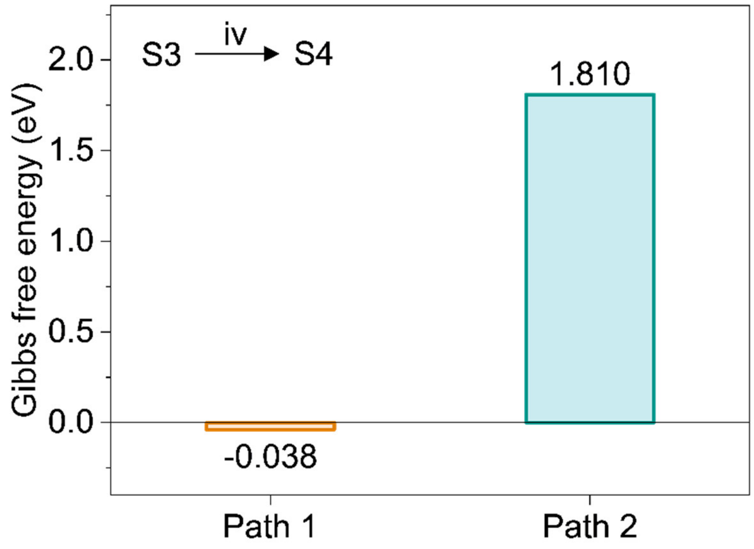

Supplementary Fig. 33. Calculated the free energy for the liberation of •OOH in step iv by ICTO in a POD-like pathway. Source data are provided as a Source Data file.

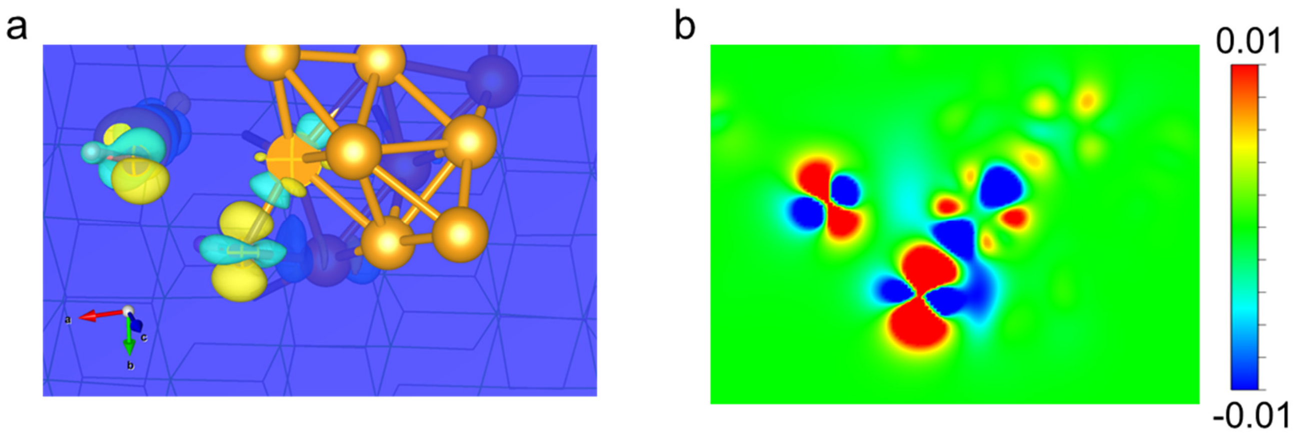

Supplementary Fig. 34. 2D isosurface map of the oxygen defect in ICTO with a unit of e·Bohr^{-3} (cyan and yellow are employed to denote charge depletion and accumulation, respectively).

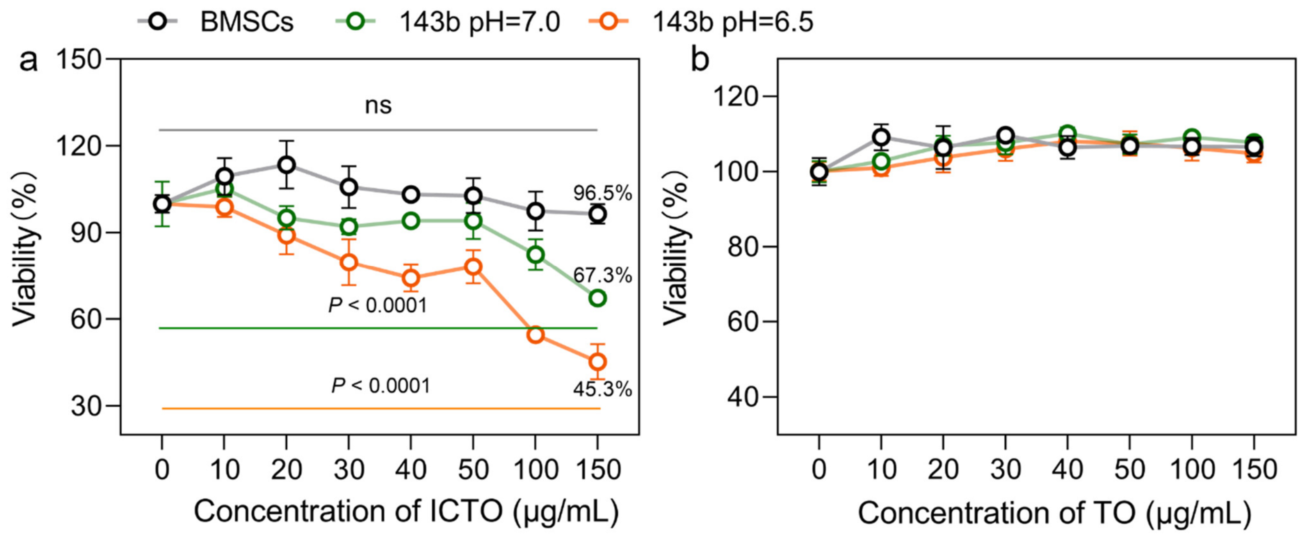

Supplementary Fig. 35. CCK-8 assay after series of concentrations of ICTO (**a**) or TO (**b**) interventions to 143b cells or BMSCs (n = 3 biologically independent replicates). Data are presented as mean ± SD, and ns represents no significant difference; statistical significance was calculated using one-way ANOVA followed by Tukey's post-hoc test for multiple comparisons; all tests were two-sided. Source data are provided as a Source Data file.

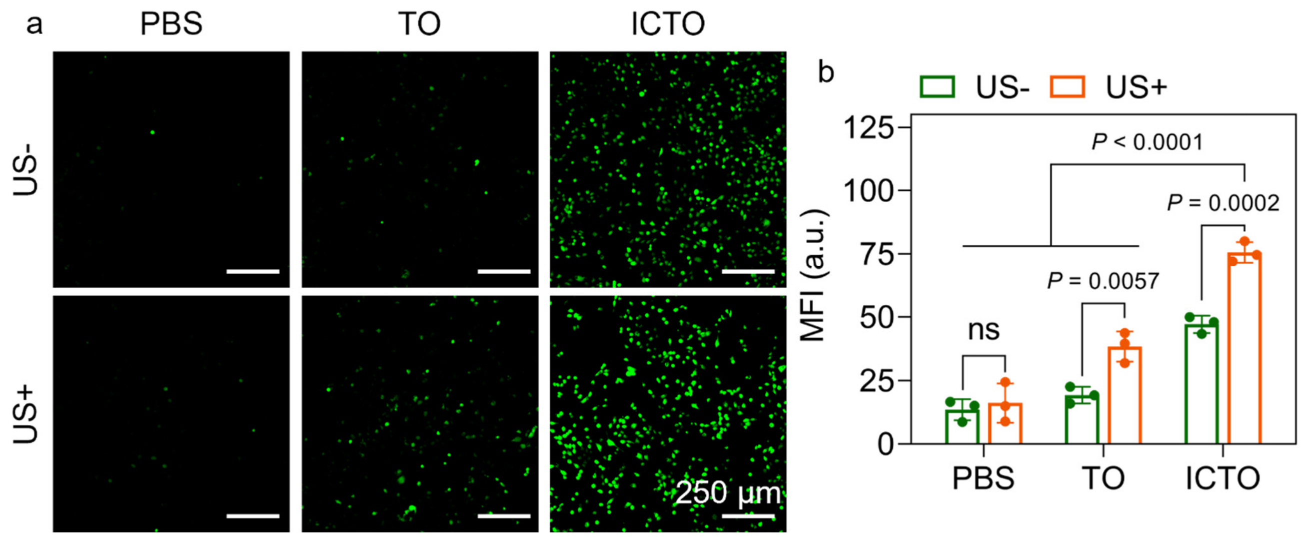

Supplementary Fig. 36. a DCFH-DA staining of the 143b cells incubated with HS or TO particles with or without US irradiation. The images were representative of three independently repeated experiments from each group. **b** Quantitative analysis of the integrated density of the green fluorescence (n = 3 biologically independent replicates). Data are presented as mean ± SD, and ns represents no significant difference; statistical significance was calculated using one-way ANOVA followed by Tukey's post-hoc test for multiple comparisons; all tests were two-sided. Source data are provided as a Source Data file.

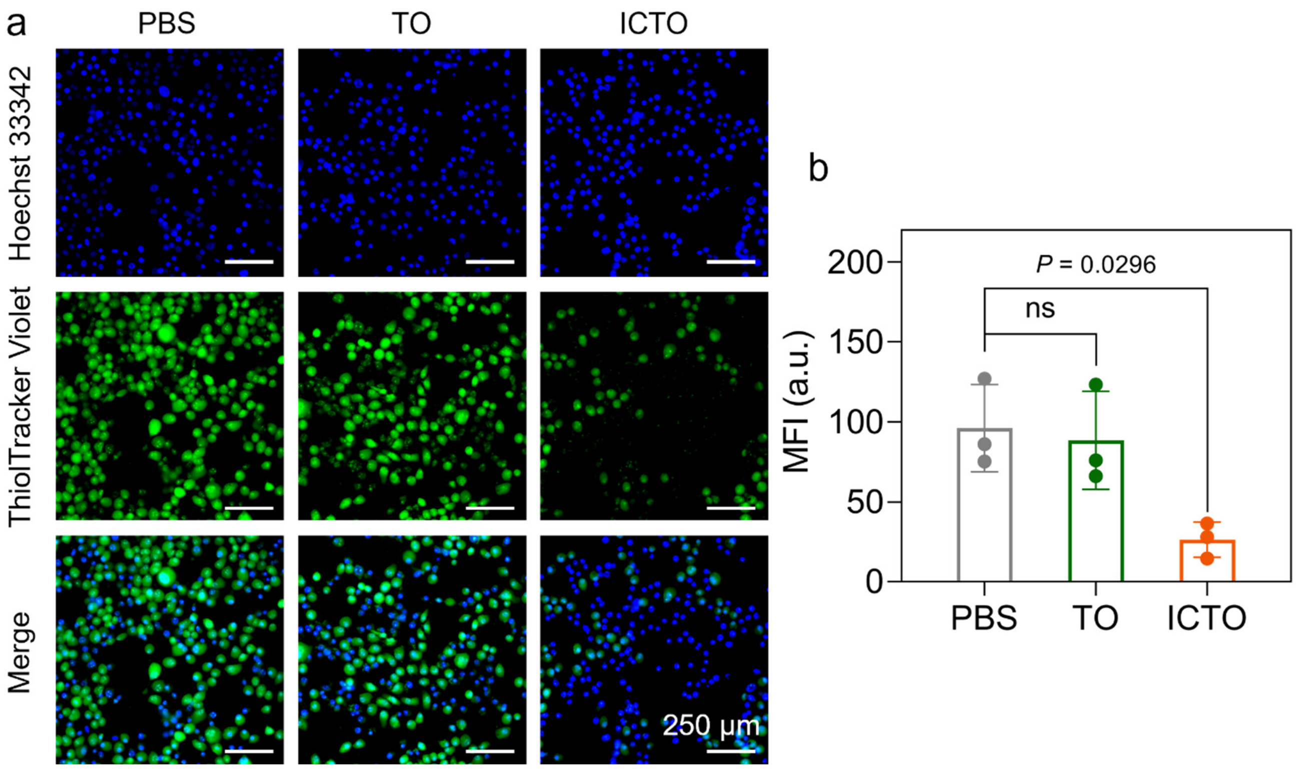

Supplementary Fig. 37. a ThiolTracker Violet staining of the intracellular GSH level of 143b cells incubated with ICTO or TO particles. Nuclei were labeled by Hoechst 33342. The images were representative of three independently repeated experiments from each group. **b** Quantitative analysis of the MFI of the green fluorescence (n = 3 biologically independent replicates). Data are presented as mean ± SD, and ns represents no significant difference; statistical significance was calculated using one-way ANOVA followed by Tukey's post-hoc test for multiple comparisons; all tests were two-sided. Source data are provided as a Source Data file.

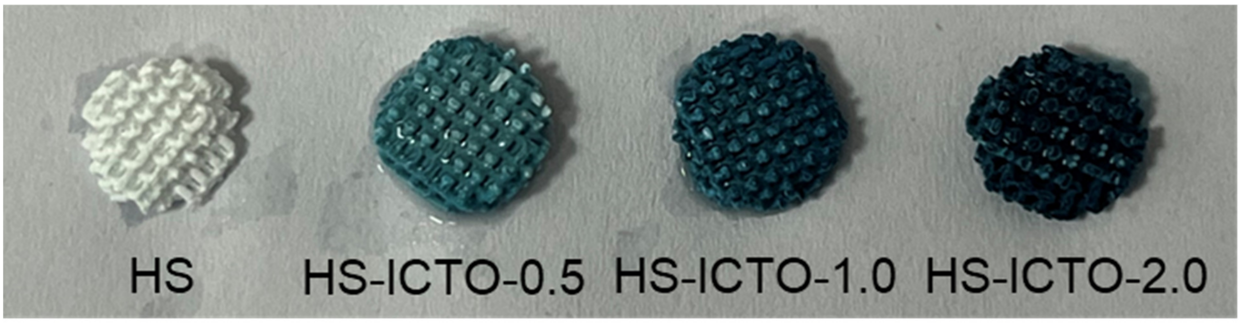

Supplementary Fig. 38. Macroscopic images of HS-ICTO-x after reaction with TMB.

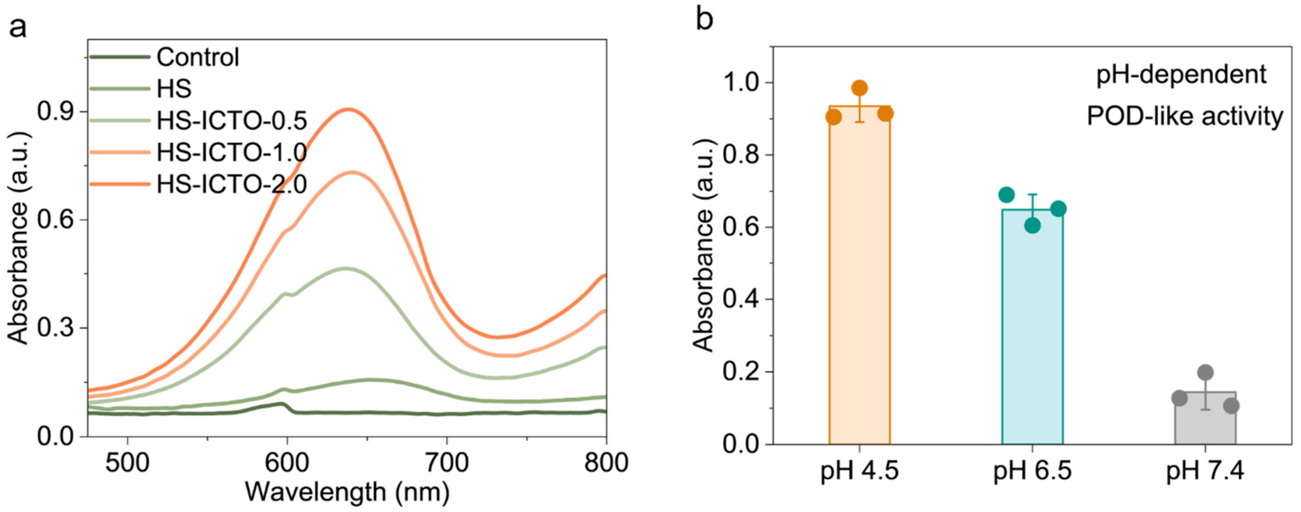

Supplementary Fig. 39. **a** UV–vis absorption spectra of POD-like activity of HS-ICTO-x. **b** Quantitative analysis of POD-like activity of HS-ICTO-2.0 at different pH conditions (n = 3 independent experiments, data are presented as mean ± SD). Source data are provided as a Source Data file.

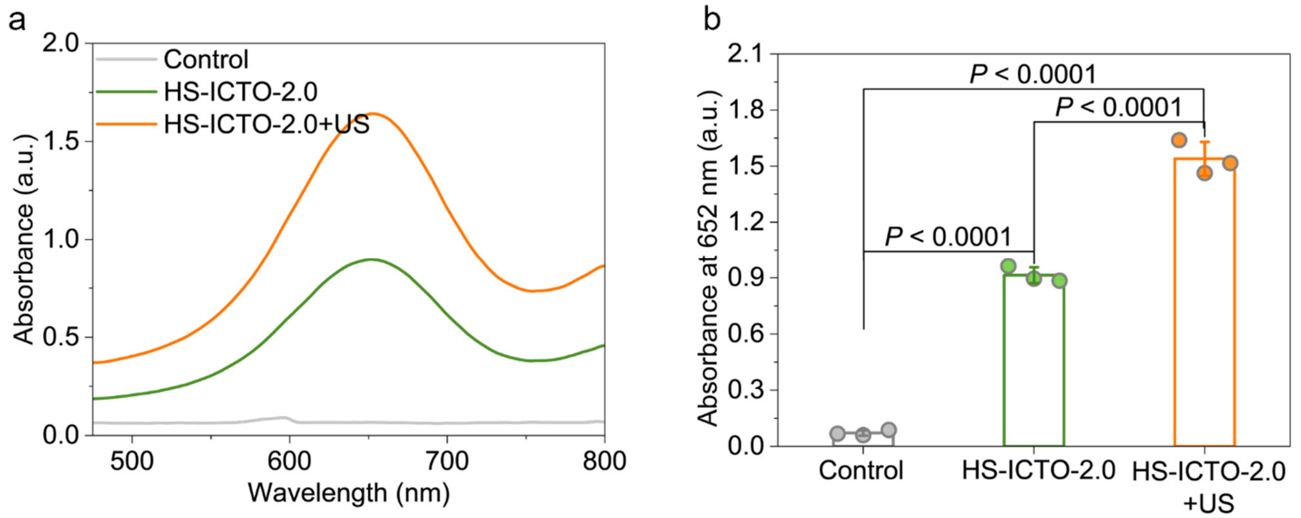

Supplementary Fig. 40. Enhanced ROS-generating activity of HS-ICTO induced by additional US (n = 3 independent experiments, data are presented as mean ± SD). Statistical significance was calculated using one-way ANOVA followed by Tukey's post-hoc test for multiple comparisons; all tests were two-sided. Source data are provided as a Source Data file.

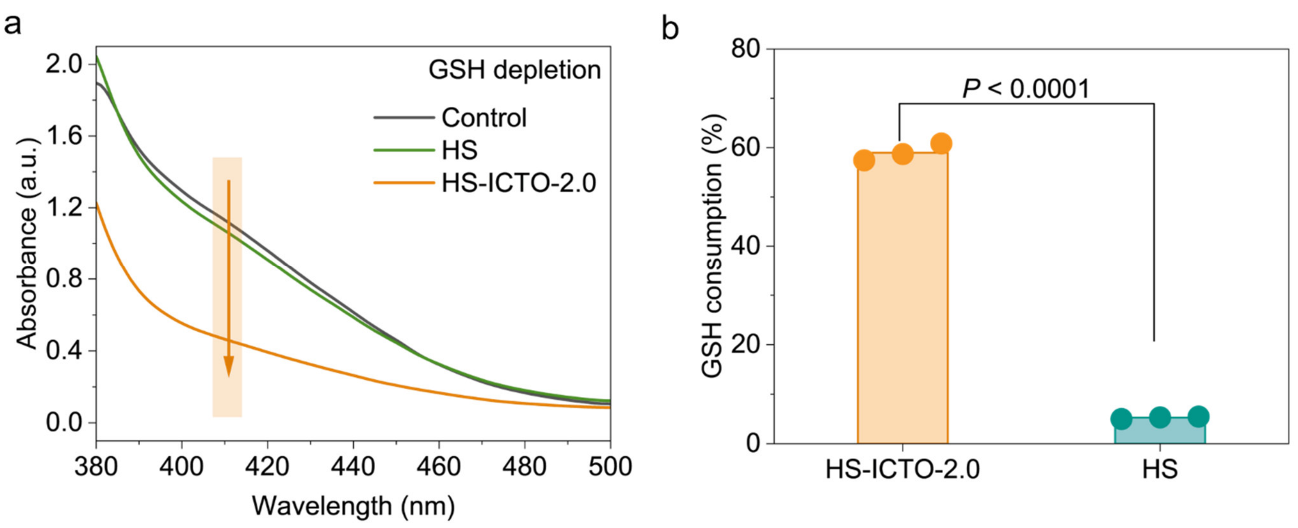

Supplementary Fig. 41. a GSH depletion ability of HS-ICTO and HS with DTNB as the trapping agent of -SH in GSH. **b** Quantitative analysis of GSH consumption ratios of HS-ICTO and HS (n = 3 independent experiments, data are presented as mean ± SD). The assessment of *P*-values is performed by a two-tailed Student's *t*-test. Source data are provided as a Source Data file.

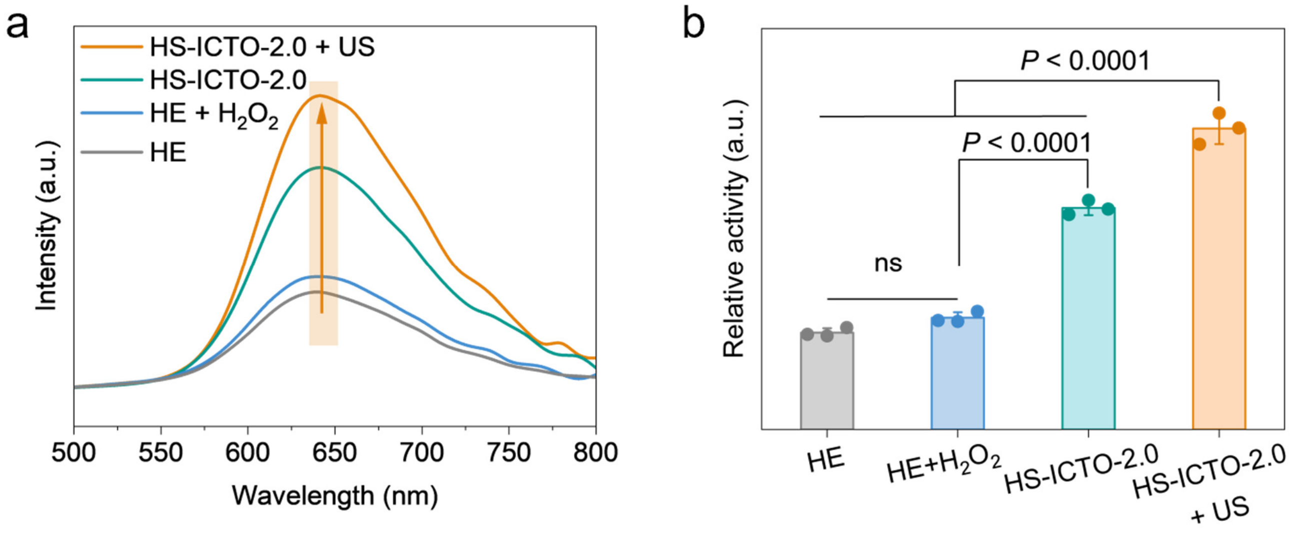

Supplementary Fig. 42. a The spectra of $•O_2^-$ generation and sonication enhancement after co-culture with HS-ICTO detected by HE probes. **b** Relative amount of $•O_2^-$ generation after co-culture with various treatment groups (n = 3 independent experiments, data are presented as mean ± SD; US: 2.0 W/cm^2, 1 MHz, 30% duty cycle). Statistical significance was calculated using one-way ANOVA followed by Tukey's post-hoc test for multiple comparisons, and ns represents no significant difference; all tests were two-sided. Source data are provided as a Source Data file.

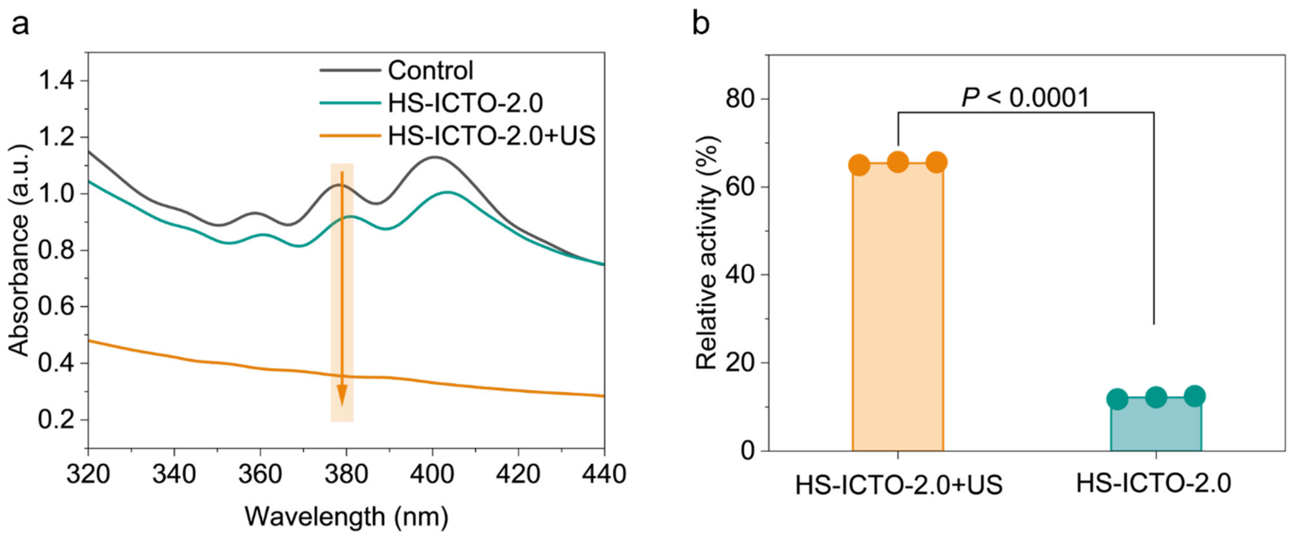

Supplementary Fig. 43. a Sono-stimulated catalytic oxidation of DPA after co-culture with HS-ICTO. **b** Relative activity of $^1O_2$ generation after sono-activation (n = 3 independent experiments, data are presented as mean ± SD; US: 2.0 W/cm^2, 1 MHz, 30% duty cycle). The assessment of *P*-values is performed by a two-tailed Student's *t*-test. Source data are provided as a Source Data file.

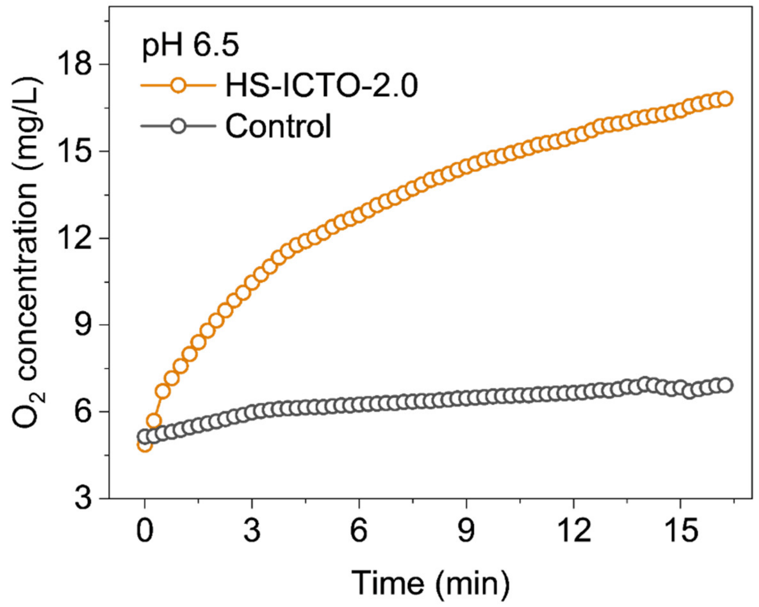

Supplementary Fig. 44. Real-time O_2 generation curve of HS-ICTO in the presence of H_2O_2 at pH 6.5. Source data are provided as a Source Data file.

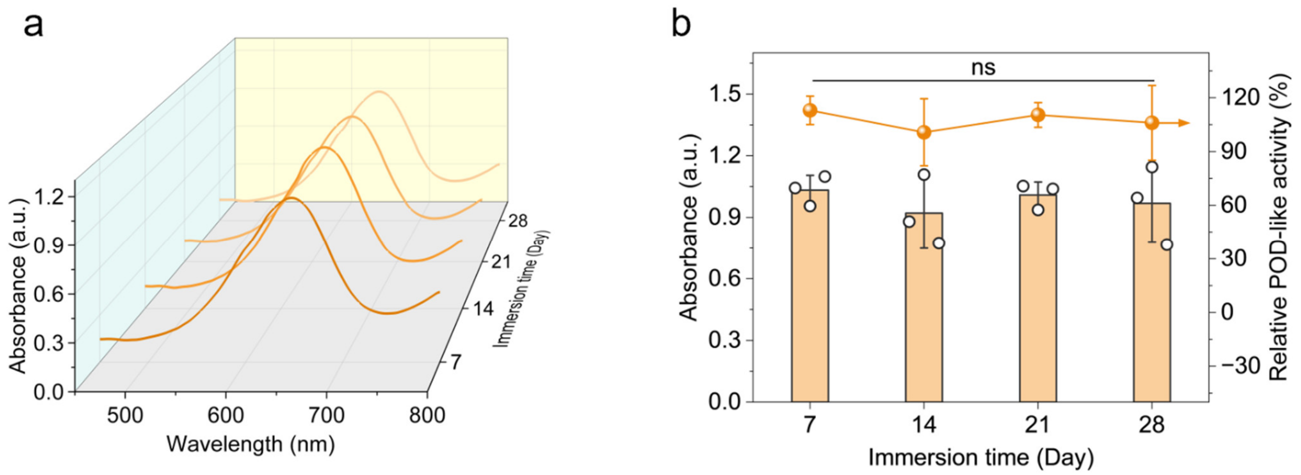

Supplementary Fig. 45. POD-like activity measurement of HS-ICTO after immersion for different times. Data are presented as mean ± SD, and ns represents no significant difference; statistical significance was calculated using one-way ANOVA followed by Tukey's post-hoc test for multiple comparisons; all tests were two-sided. Source data are provided as a Source Data file.

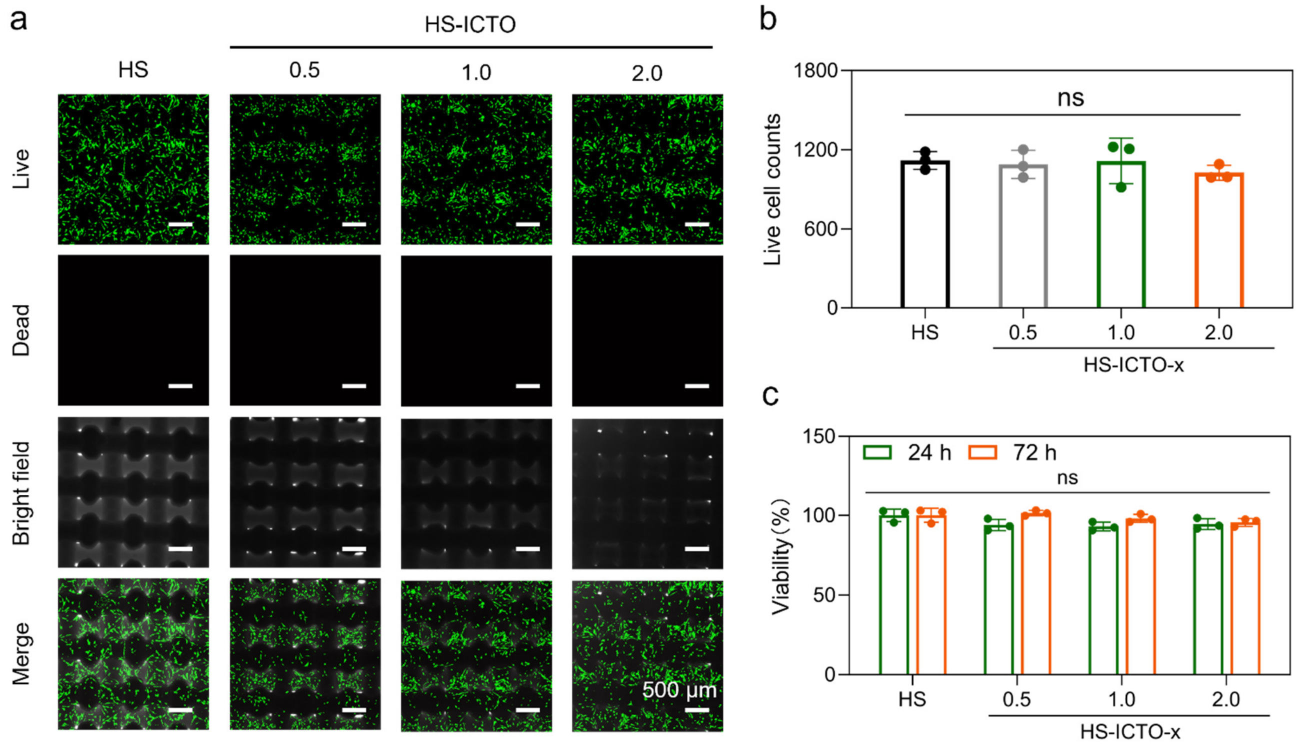

Supplementary Fig. 46. a Representative confocal laser scanning microscopy (CLSM) images of the BMSCs stained with calcein-AM/PI on different scaffolds on day 3. The images were representative of three independently repeated experiments from each group. **b** Quantitative analysis of the live cell numbers (n = 3 biologically independent replicates). **c** CCK-8 viability assessment of BMSCs seeding

on different scaffolds after 24 h and 72 h (n = 3 biologically independent replicates). Data are presented as mean ± SD, and ns represents no significant difference; statistical significance was calculated using one-way ANOVA followed by Tukey's post-hoc test for multiple comparisons; all tests were two-sided. Source data are provided as a Source Data file.

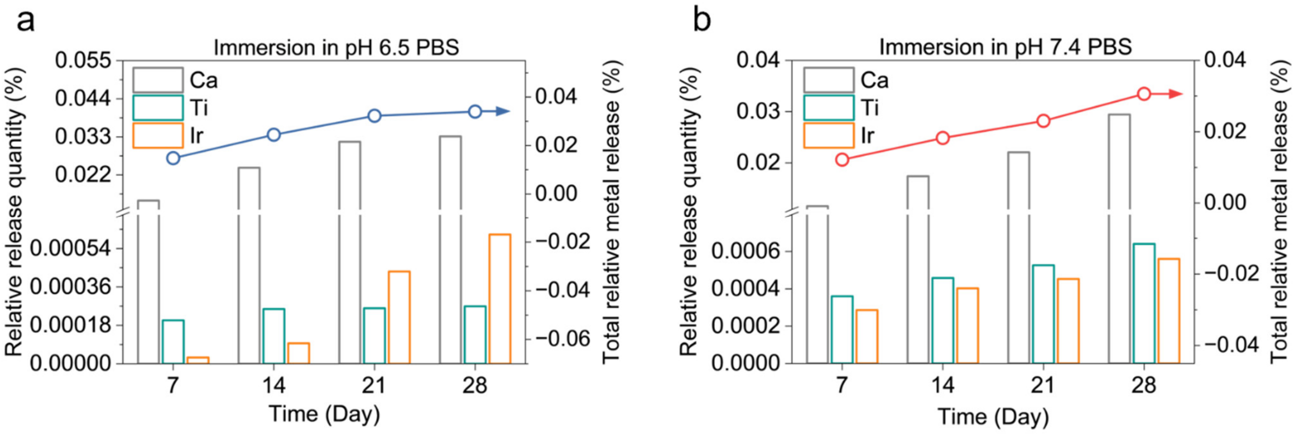

Supplementary Fig. 47. Percentage of metal release at different immersion times based on HS-ICTO-2.0 scaffolds. Source data are provided as a Source Data file.

Notably, to evaluate the potential cumulative metal ion toxicity of both HS-ICTO and internalized ICTO nanoparticles, we immersed HS-ICTO in PBS. Supernatants were collected at predetermined time points, and the release concentrations of Ir^{3+}, Ti^{4+}, and Ca^{2+} were quantitatively analyzed using inductively coupled plasma atomic emission spectroscopy (ICP-AES). The experimental results reveal exceptional ionic stability, with cumulative leaching rates not exceeding 0.035% over a four-week period under both mildly acidic (pH 6.5) and physiological (pH 7.4) conditions, indicating negligible ionic toxicity potential and substantiating the superior biocompatibility and safety profile of scaffolds.

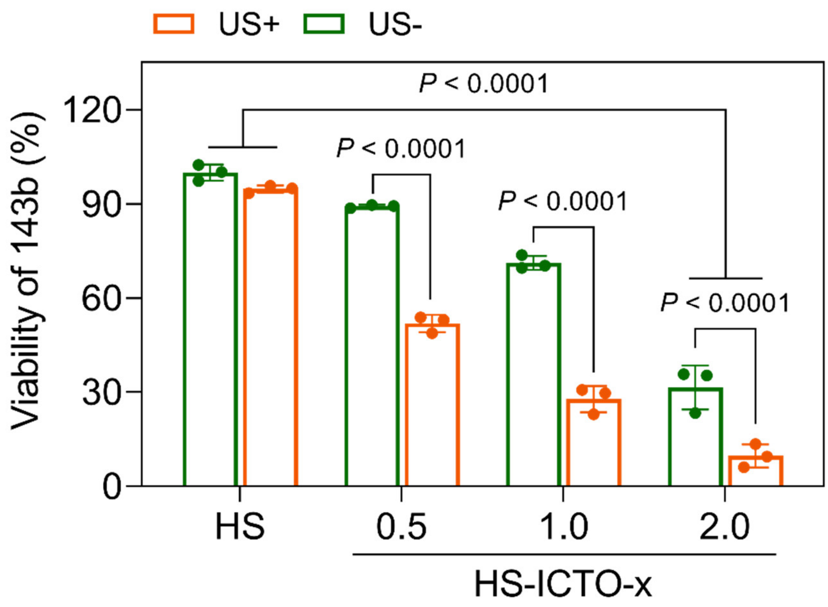

Supplementary Fig. 48. a CCK-8 viability assessment of 143b cells on different scaffolds with or without US treatments (n = 3 biologically independent replicates). Data are presented as mean ± SD; statistical significance was calculated using one-way ANOVA followed by Tukey's post-hoc test for multiple comparisons; all tests were two-sided. Source data are provided as a Source Data file.

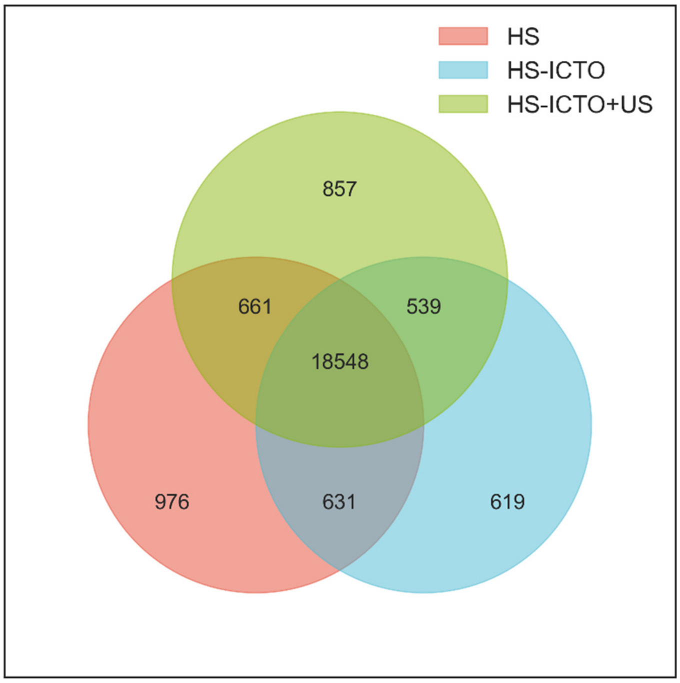

Supplementary Fig. 49. Venn diagram of the differently expressed gene profiles. The data were representative of three biologically independent samples from each group.

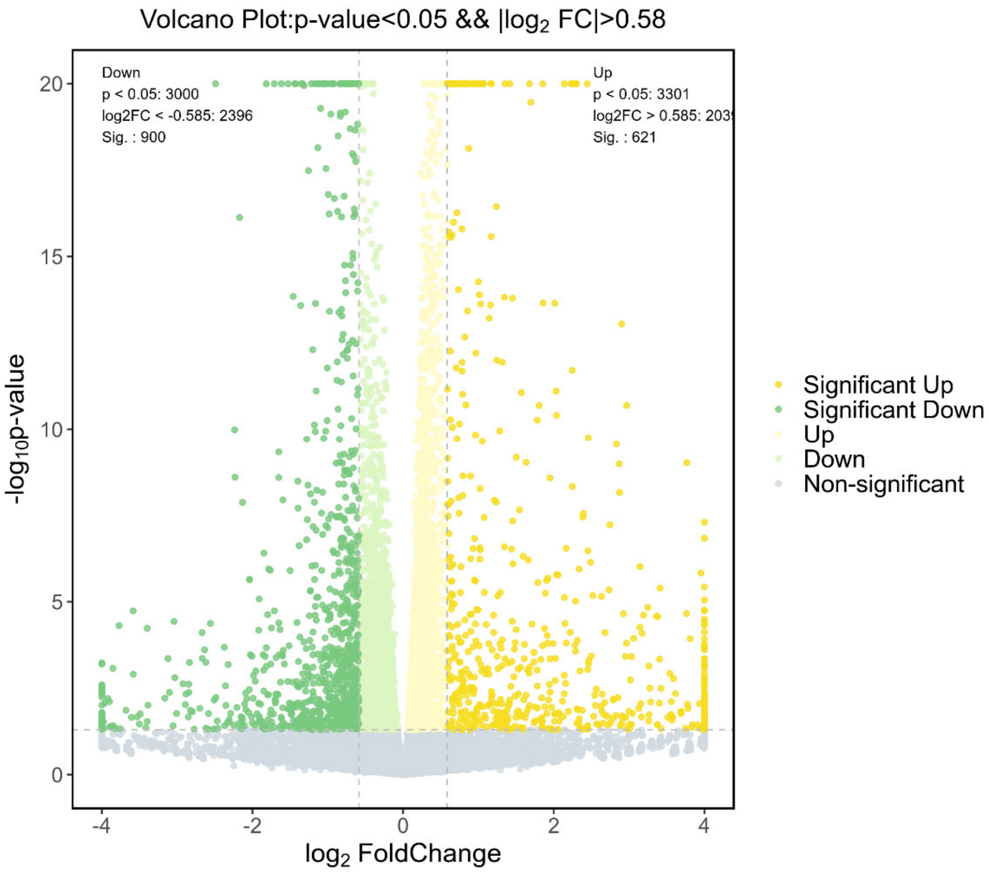

Supplementary Fig. 50. Volcano plot that demonstrates differential gene expression in HS-ICTO + US *vs* HS-ICTO comparison. The data were representative of three biologically independent samples from each group. *P*-values was obtained from two-sided DESeq2 test without multiple comparison.

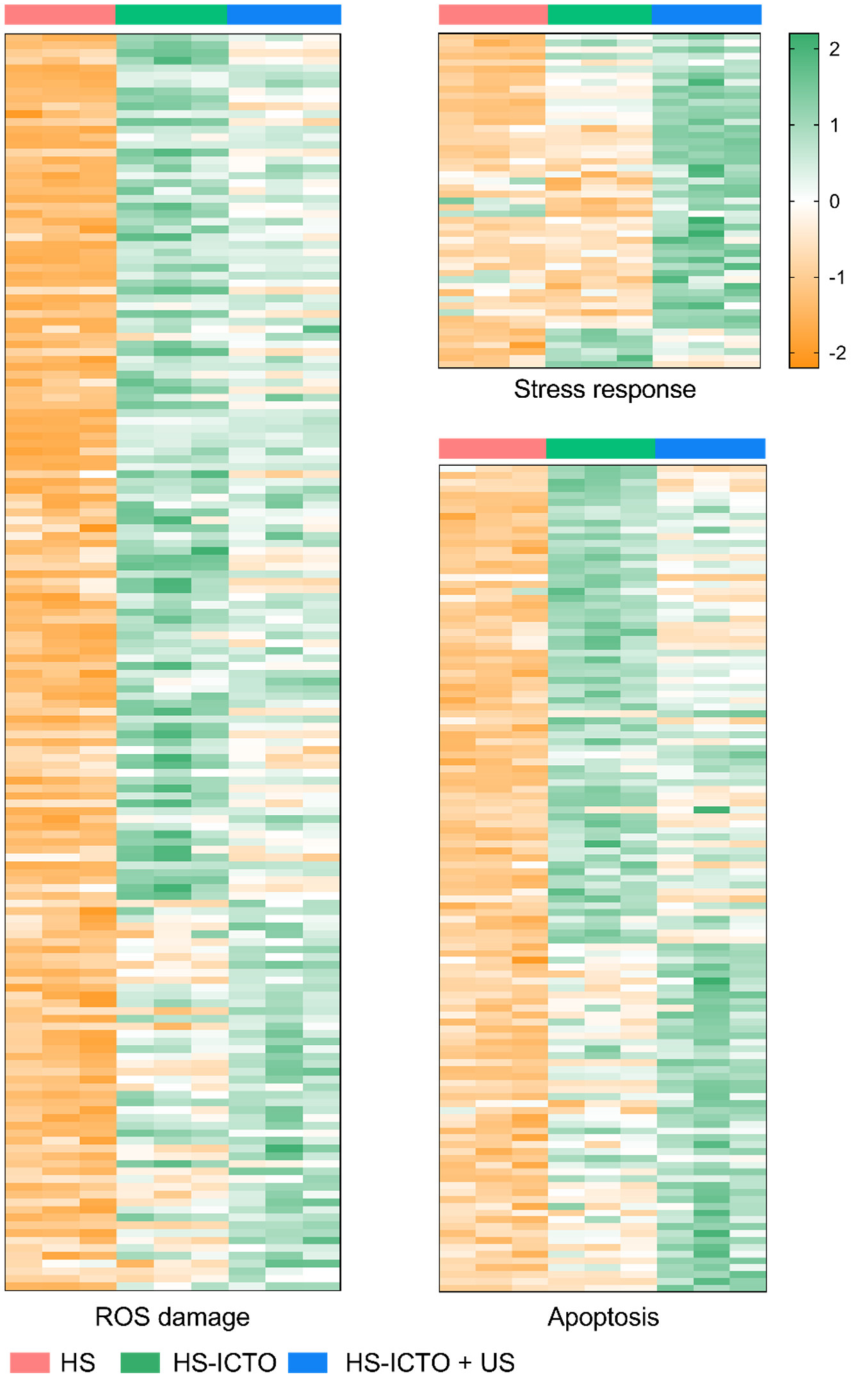

Supplementary Fig. 51. Heat maps illustrating the differential expression data involved in ROS damage, stress response, and apoptosis. The data were representative of three biologically independent samples from each group.

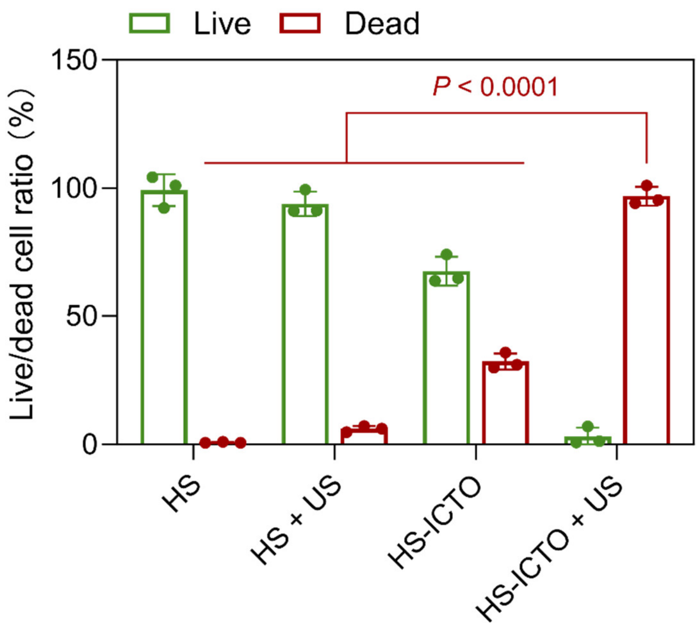

Supplementary Fig. 52. Quantitative analysis of the live and dead cell numbers in CLSM observation of 143b cells seeded on different scaffolds with or without US irradiation (n = 3 biologically independent replicates). Data are presented as mean ± SD; statistical significance was calculated using one-way ANOVA followed by Tukey's post-hoc test for multiple comparisons; all tests were two-sided. Source data are provided as a Source Data file.

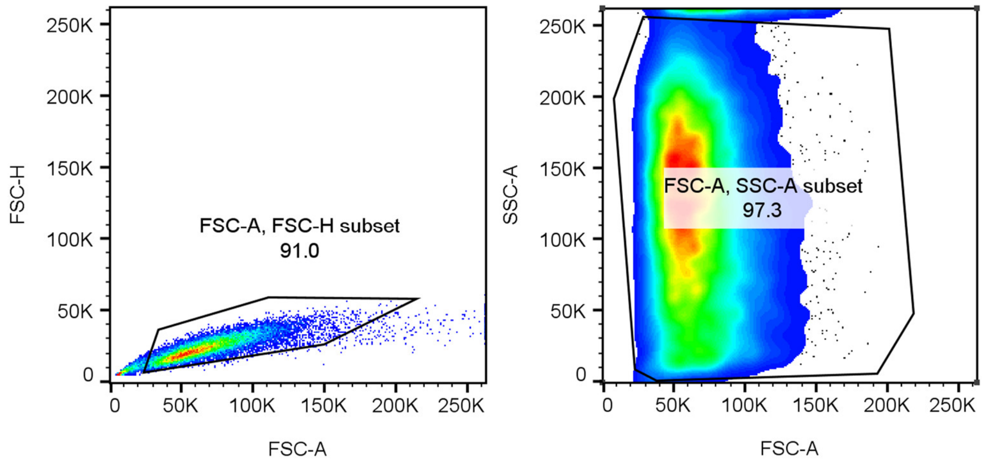

Supplementary Fig. 53. The gating strategy for Fig. 4h.

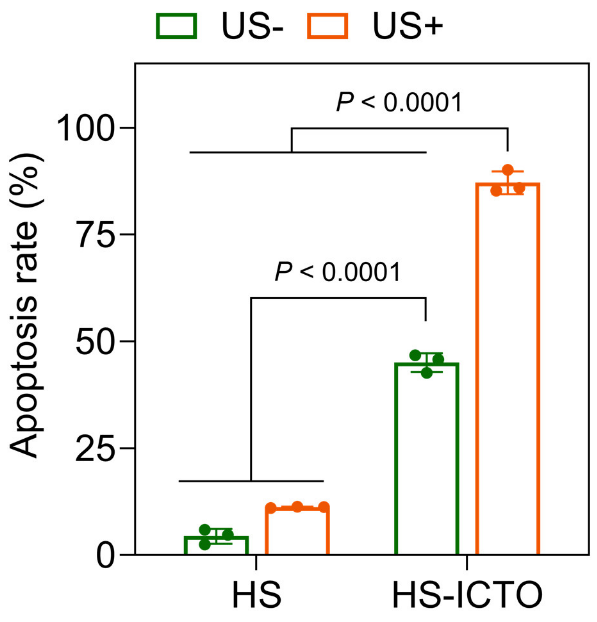

Supplementary Fig. 54. Quantitative analysis of the apoptotic cells in the flow cytometric analysis (n = 3 biologically independent replicates). Data are presented as mean ± SD; statistical significance was calculated using one-way ANOVA followed by Tukey's post-hoc test for multiple comparisons; all tests were two-sided. Source data are provided as a Source Data file.

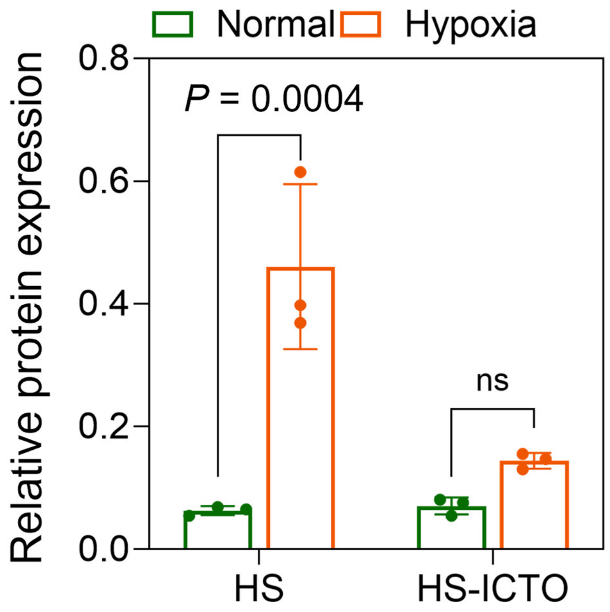

Supplementary Fig. 55. Relative protein expression of HIF-1α of 143b cells from different groups (n = 3 biologically independent replicates). Data are presented as mean ± SD, and ns represents no significant difference; statistical significance was calculated using one-way ANOVA followed by

Tukey's post-hoc test for multiple comparisons; all tests were two-sided. Source data are provided as a Source Data file.

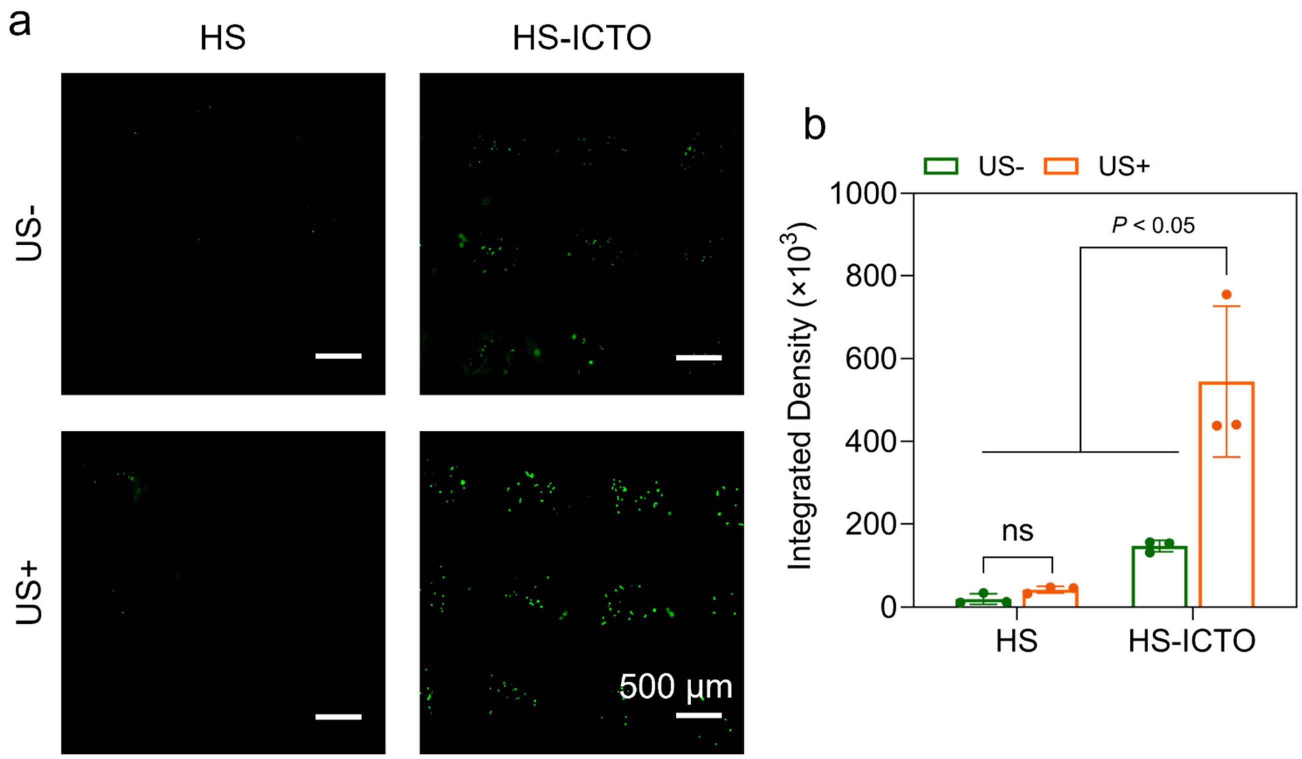

Supplementary Fig. 56. a DCFH-DA staining of the intracellular ROS accumulation in the 143b cells on different scaffolds. The images were representative of three independently repeated experiments from each group. **b** Quantitative analysis of the integrated density of the green fluorescence (n = 3 biologically independent replicates). Data are presented as mean ± SD, and ns represents no significant difference; statistical significance was calculated using one-way ANOVA followed by Tukey's post-hoc test for multiple comparisons; all tests were two-sided. Source data are provided as a Source Data file.

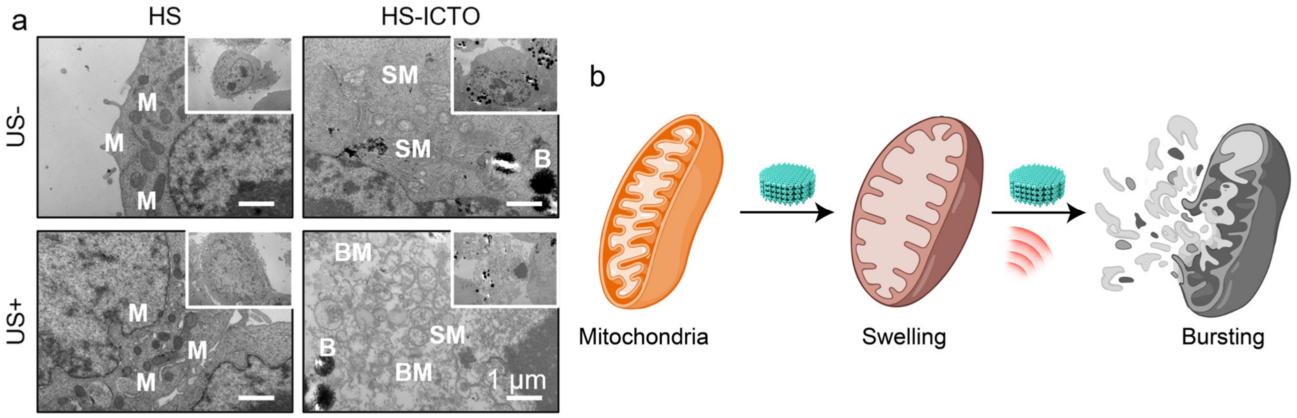

Supplementary Fig. 57. a TEM observation of the 143b cells collected from HS or HS-ICTO. (M: healthy mitochondria. SM: swelling mitochondria. BM: bursting mitochondria). The images were representative of three independently repeated experiments from each group. **b** Schematic illustration of the CDT and SDT mitochondria damage process.

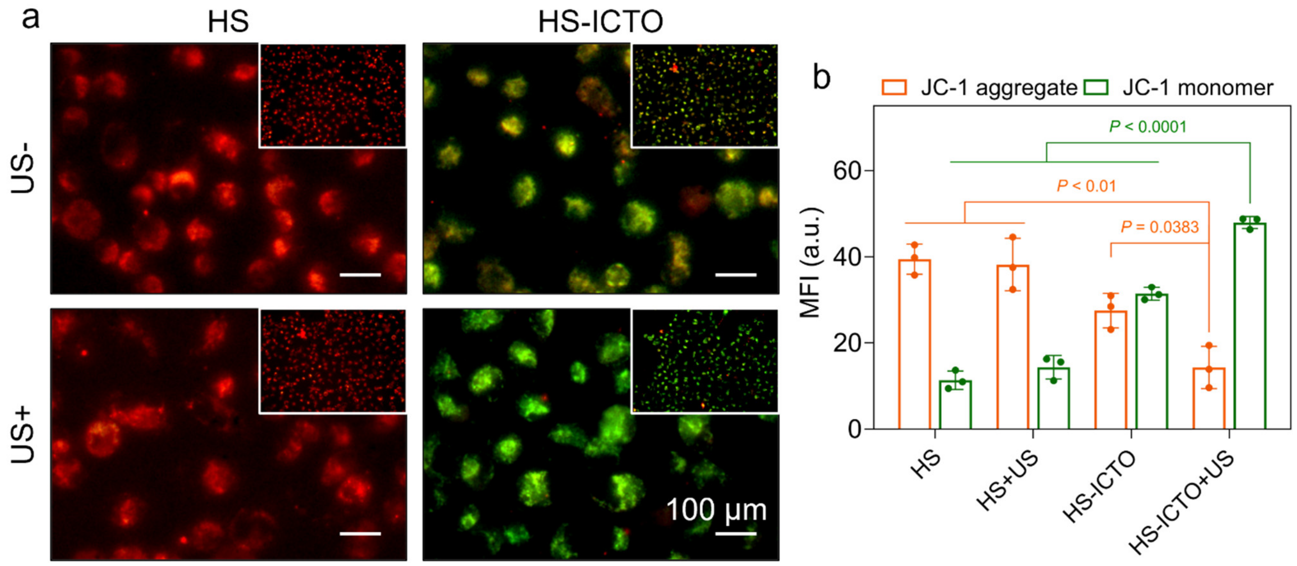

Supplementary Fig. 58. a JC-1 staining of the 143b cells after different stimulations for 24 h. (Red: JC-1 aggregate. Green: JC-1 monomer). The images were representative of three independently repeated experiments from each group. **b** Quantitative analysis of the MFI of JC-1 aggregate or JC-1 monomer (n = 3 biologically independent replicates). Data are presented as mean ± SD, and ns represents no significant difference; statistical significance was calculated using one-way ANOVA followed by Tukey's post-hoc test for multiple comparisons; all tests were two-sided. Source data are provided as a Source Data file.

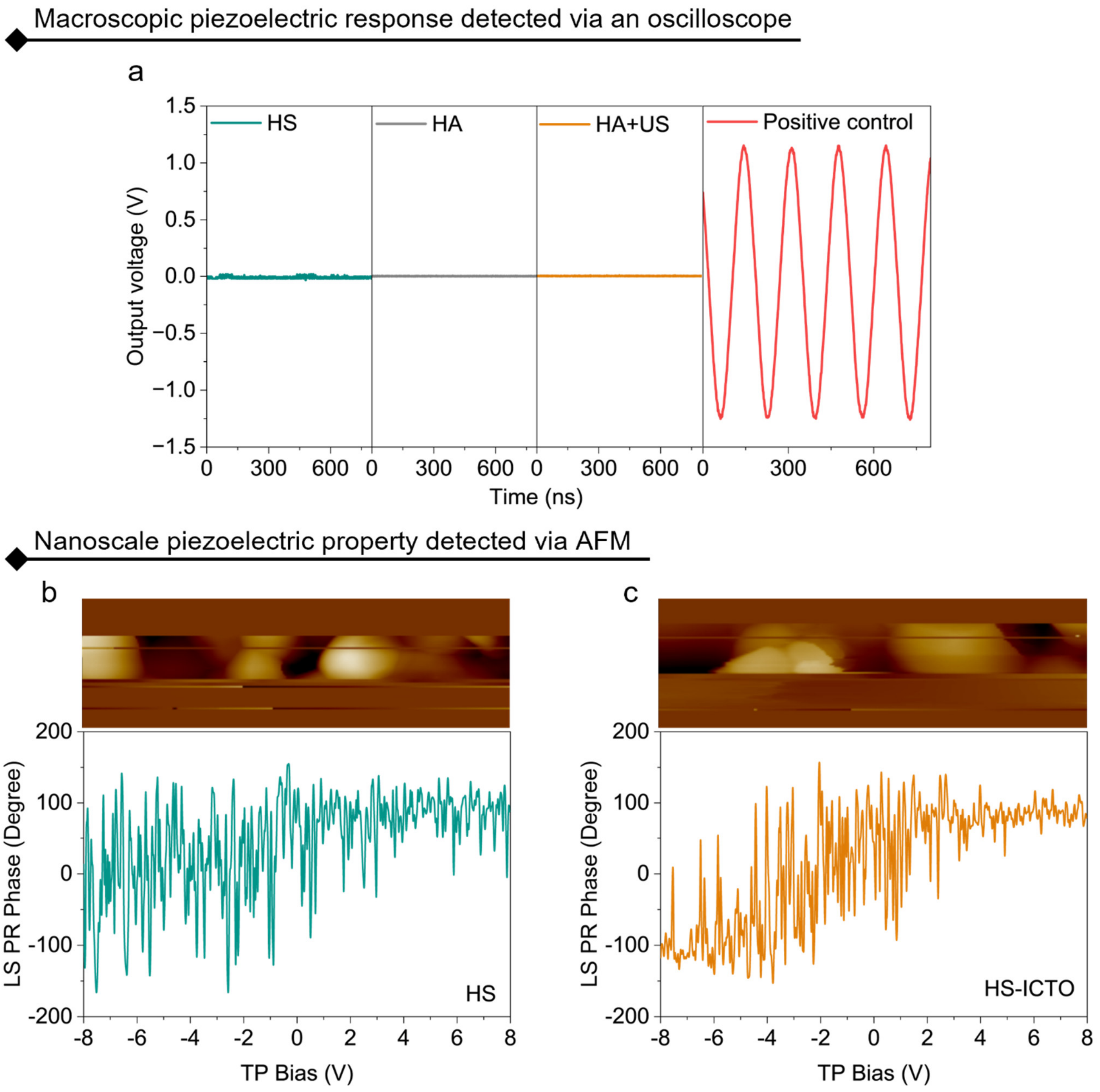

Supplementary Fig. 59. a Oscilloscope detection of electrical signals generated under vibration. Representative atomic force detection images and longitudinal signal piezoresponse phase (LS PR Phase) waves of HS (**b**) and HS-ICTO (**c**). Source data are provided as a Source Data file.

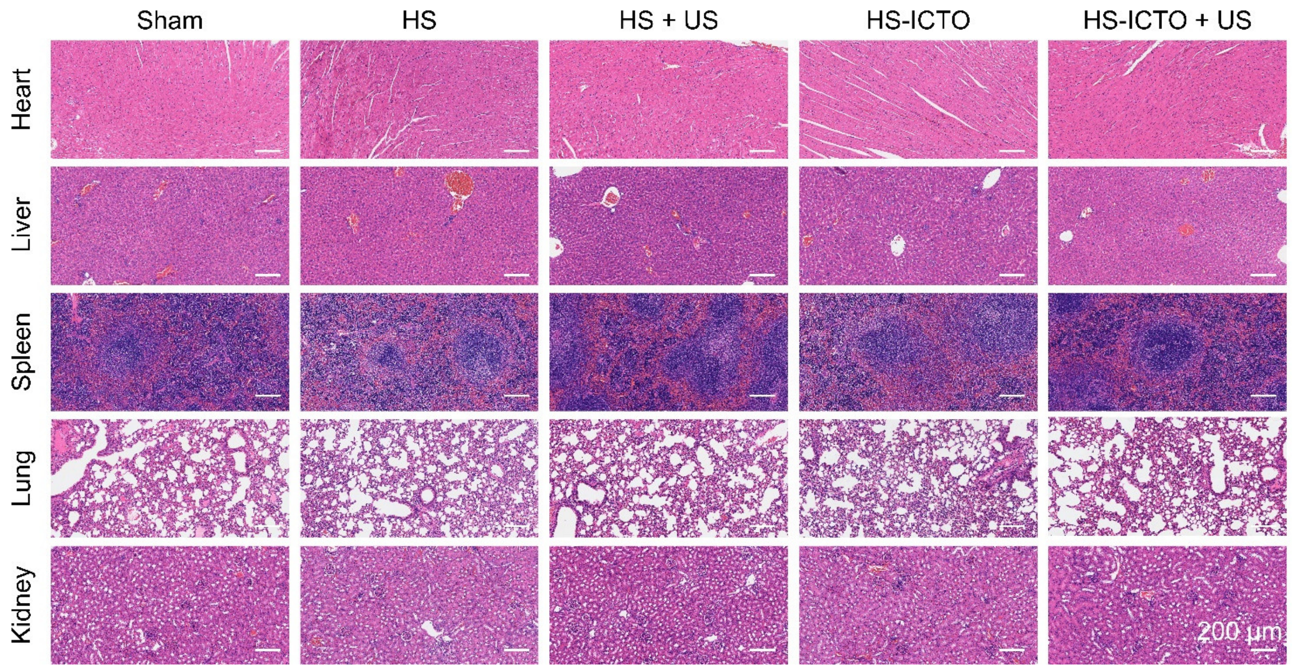

Supplementary Fig. 60. The H&E staining of the vital organs of the tumor-bearing mice. The images were representative of three independently repeated experiments from each group.

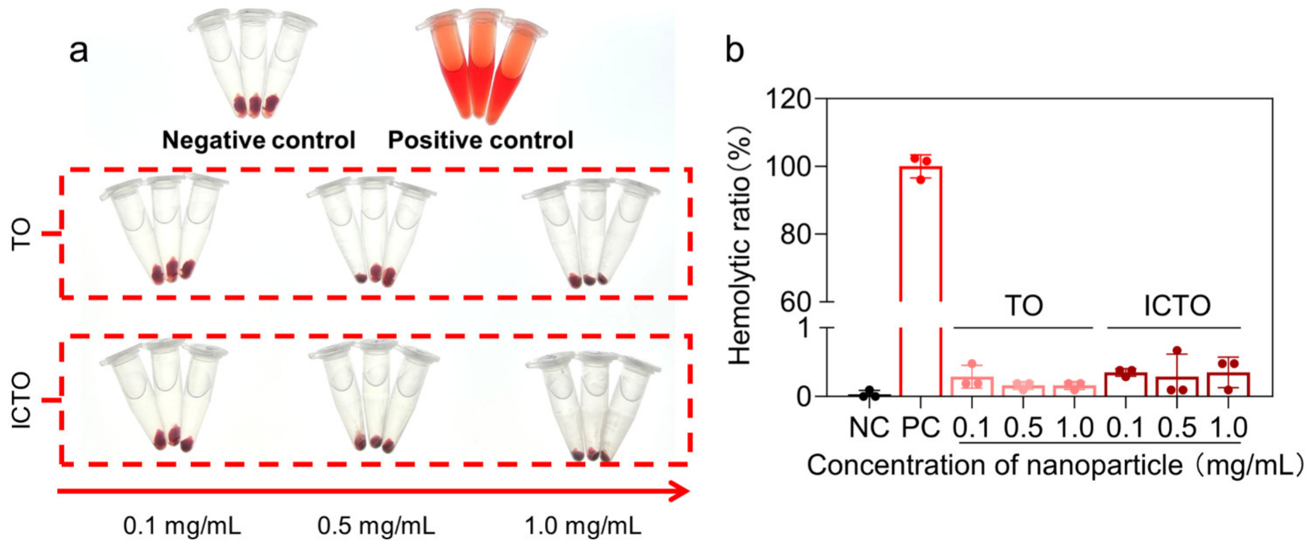

Supplementary Fig. 61. a Hemolysis test of the biocatalysts. The images were representative of three independently repeated experiments from each group. **b** Quantitative analysis of the hemolytic ratios, which were far below 5%, thus indicating good blood compatibility (n = 3 biologically independent replicates, data are presented as mean ± SD). Source data are provided as a Source Data file.

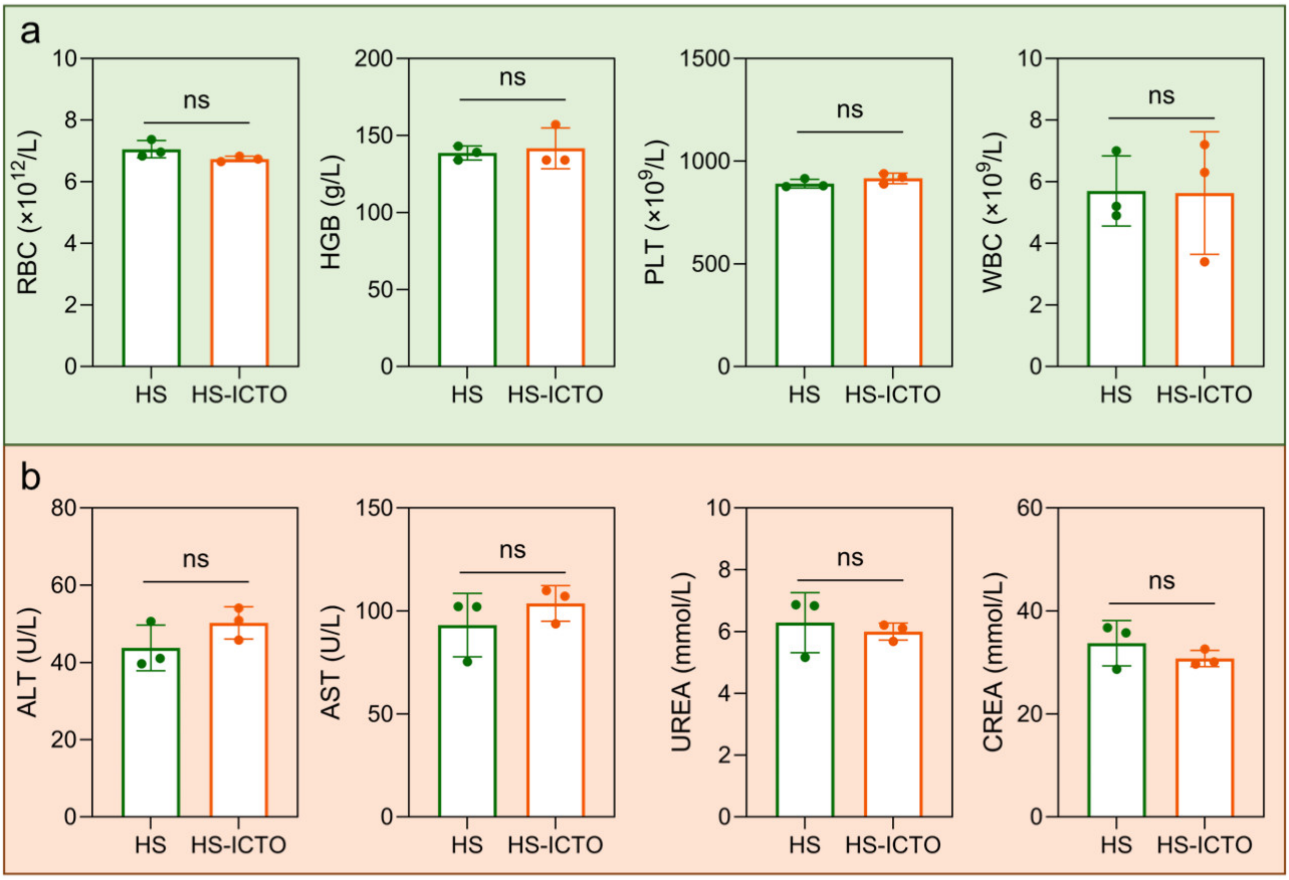

Supplementary Fig. 62. a Blood routine test results including red blood cell count (RBC), hemoglobin (HGB), platelets (PLT), and white blood cell count (WBC). **b** Biochemistry test results including alanine aminotransferase (ALT), aspartate aminotransferase (AST), urea, and creatinine (CREA) (n = 3 biologically independent replicates). Data are presented as mean ± SD, and ns represents no significant difference; statistical significance was calculated using two-tailed Student's *t*-test. Source data are provided as a Source Data file.

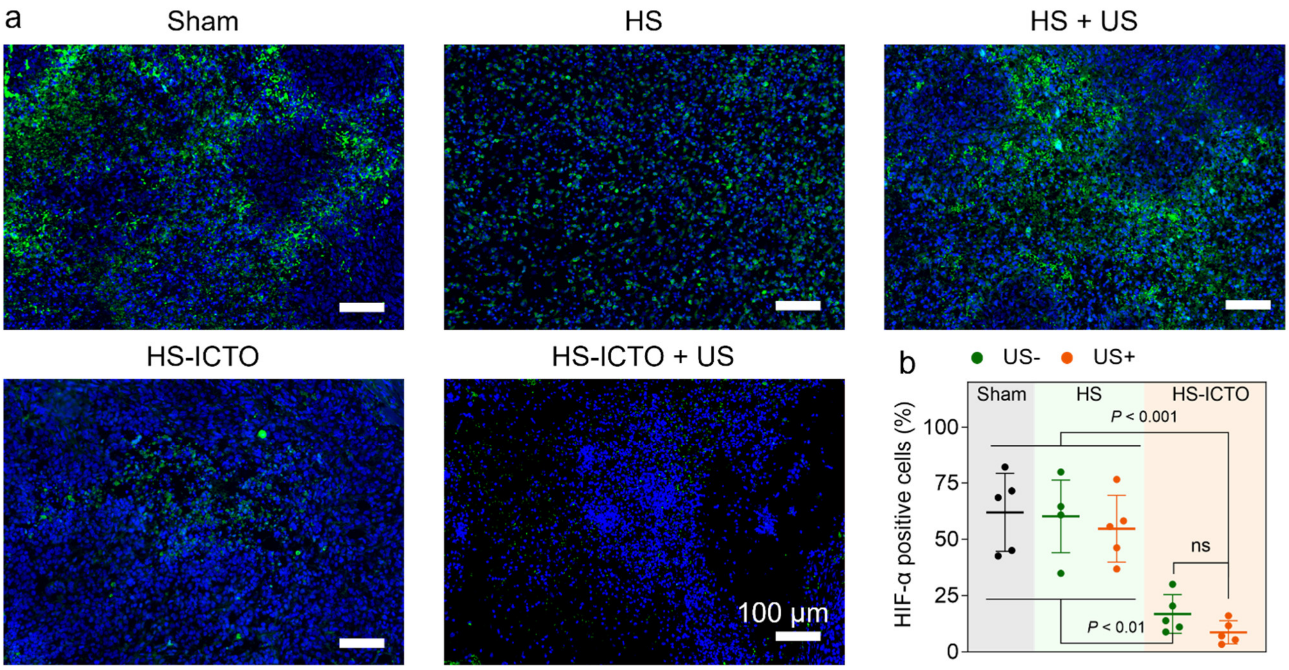

Supplementary Fig. 63. a Representative HIF-1α fluorescence staining images of the tumors from different groups. The images were representative of five independently repeated experiments from each group. **b** Quantitative analysis of the HIF-1α-positive cell ratios (n = 5 biologically independent replicates). Data are presented as mean ± SD, and ns represents no significant difference; statistical significance was calculated using one-way ANOVA followed by Tukey's post-hoc test for multiple comparisons; all tests were two-sided. Source data are provided as a Source Data file.

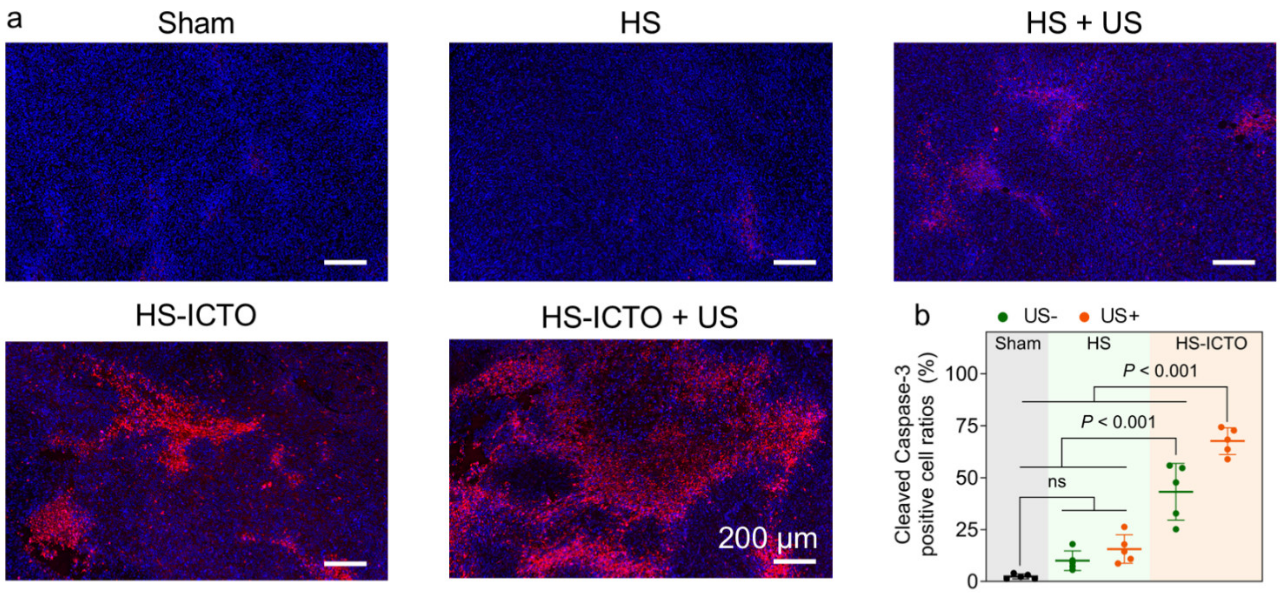

Supplementary Fig. 64. a Representative cleaved caspase-3 fluorescence staining images of the tumors from different groups. The images were representative of five independently repeated experiments from each group. **b** Quantitative analysis of the cleaved caspase-3-positive cell ratios (n = 5 biologically independent replicates). Data are presented as mean ± SD, and ns represents no significant difference; statistical significance was calculated using one-way ANOVA followed by Tukey's post-hoc test for multiple comparisons; all tests were two-sided. Source data are provided as a Source Data file.

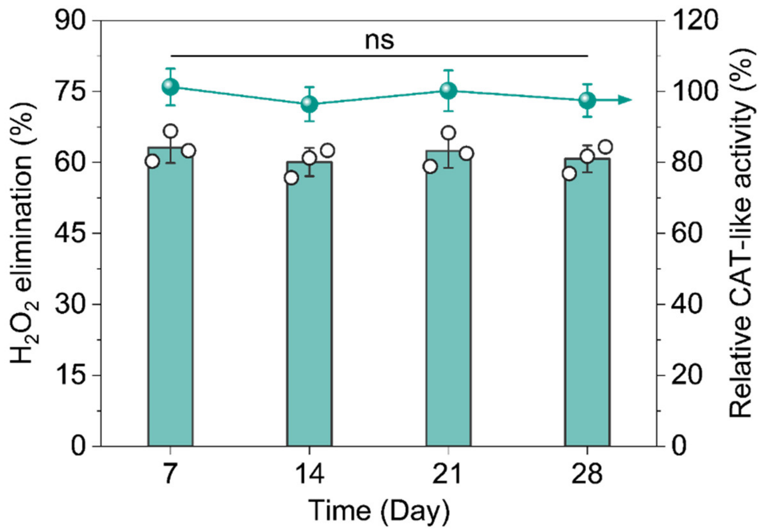

Supplementary Fig. 65. CAT-like activity measurement of HS-ICTO after immersion for different times. Data are presented as mean ± SD, and ns represents no significant difference; statistical significance was calculated using one-way ANOVA followed by Tukey's post-hoc test for multiple comparisons; all tests were two-sided. Source data are provided as a Source Data file.

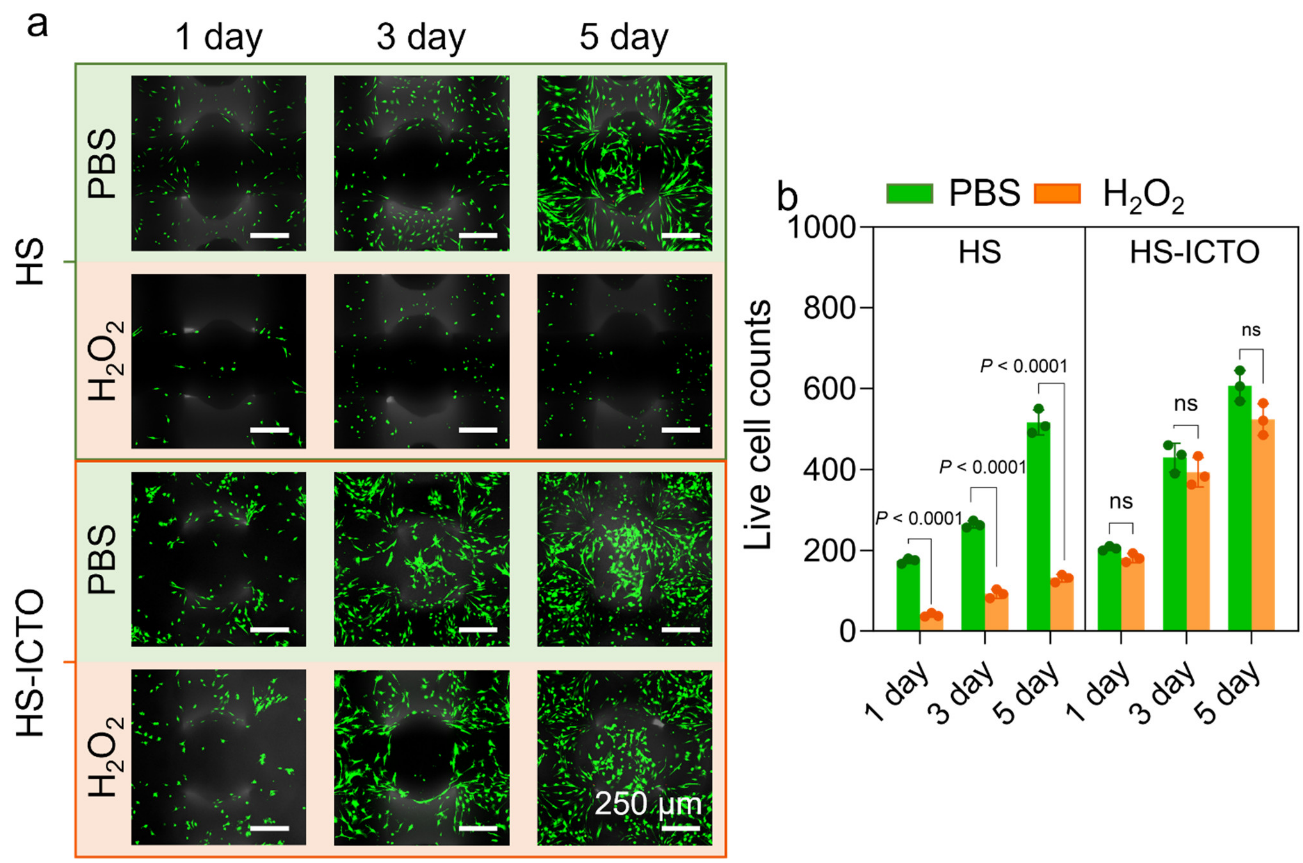

Supplementary Fig. 66. a The CLSM images of calcein-AM staining of BMSCs seeded on scaffolds on days 1, 3, and 5. The images were representative of three independently repeated experiments from each group. **b** Quantitative analysis of the live cell count in different groups (n = 3 biologically independent replicates). Data are presented as mean ± SD, and ns represents no significant difference; statistical significance was calculated using one-way ANOVA followed by Tukey's post-hoc test for multiple comparisons; all tests were two-sided. Source data are provided as a Source Data file.

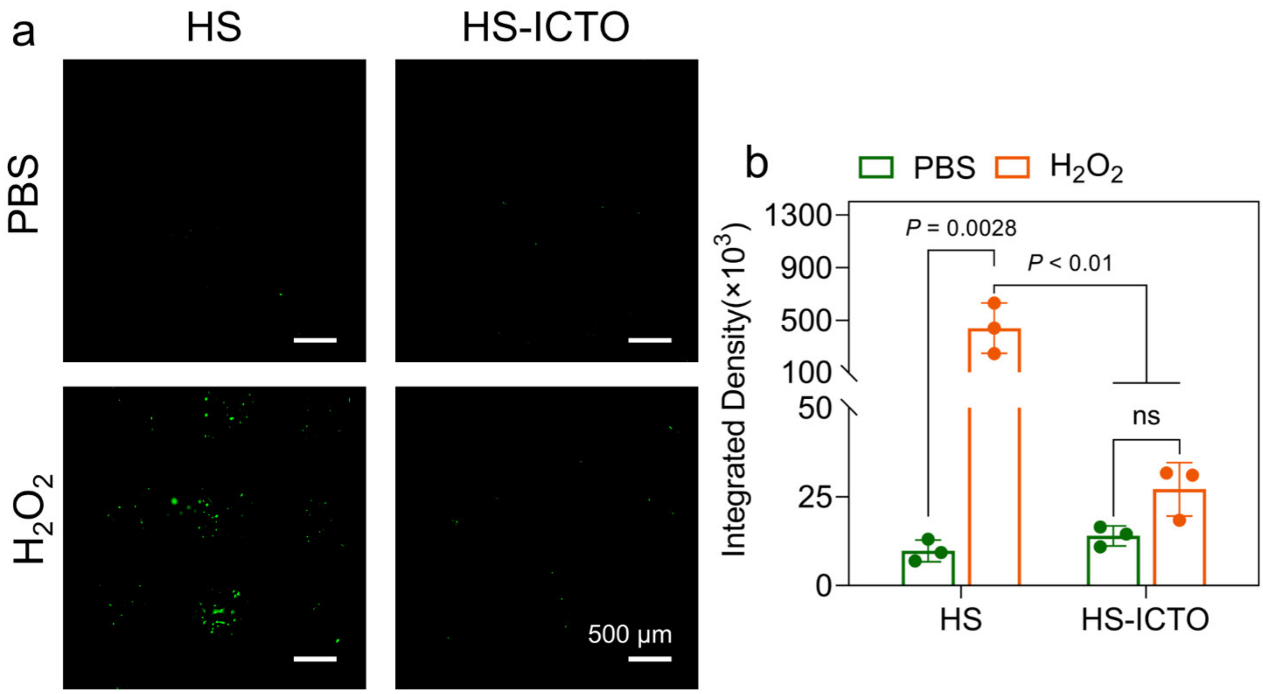

Supplementary Fig. 67. a DCFH-DA staining of the intracellular ROS of the BMSCs on different scaffolds. The images were representative of three independently repeated experiments from each group. **b** Quantitative analysis of the integrated density of the green fluorescence (n = 3 biologically independent replicates). Data are presented as mean ± SD, and ns represents no significant difference; statistical significance was calculated using one-way ANOVA followed by Tukey's post-hoc test for multiple comparisons; all tests were two-sided. Source data are provided as a Source Data file.

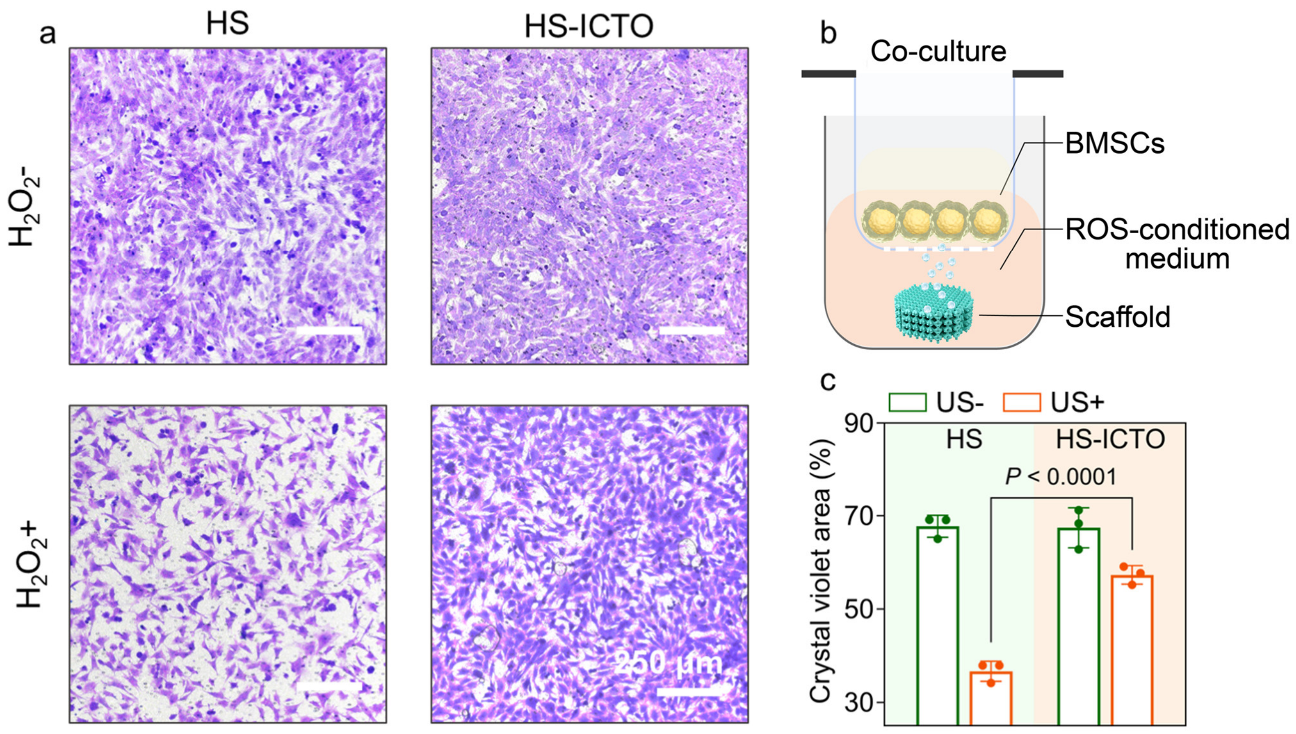

Supplementary Fig. 68. a Representative crystal violet-labeled migrated BMSCs images. **b** Illustration of the transwell migration experiment of BMSCs. The BMSCs were seeded in the upper chamber, and the ROS-conditioned complete medium was added in the lower chamber. HS or HS-ICTO was placed in the lower chamber. **c** Quantitative analysis of the crystal violet area (%) (n = 3 biologically independent replicates). Data are presented as mean ± SD; statistical significance was calculated using one-way ANOVA followed by Tukey's post-hoc test for multiple comparisons; all tests were two-sided. Source data are provided as a Source Data file.

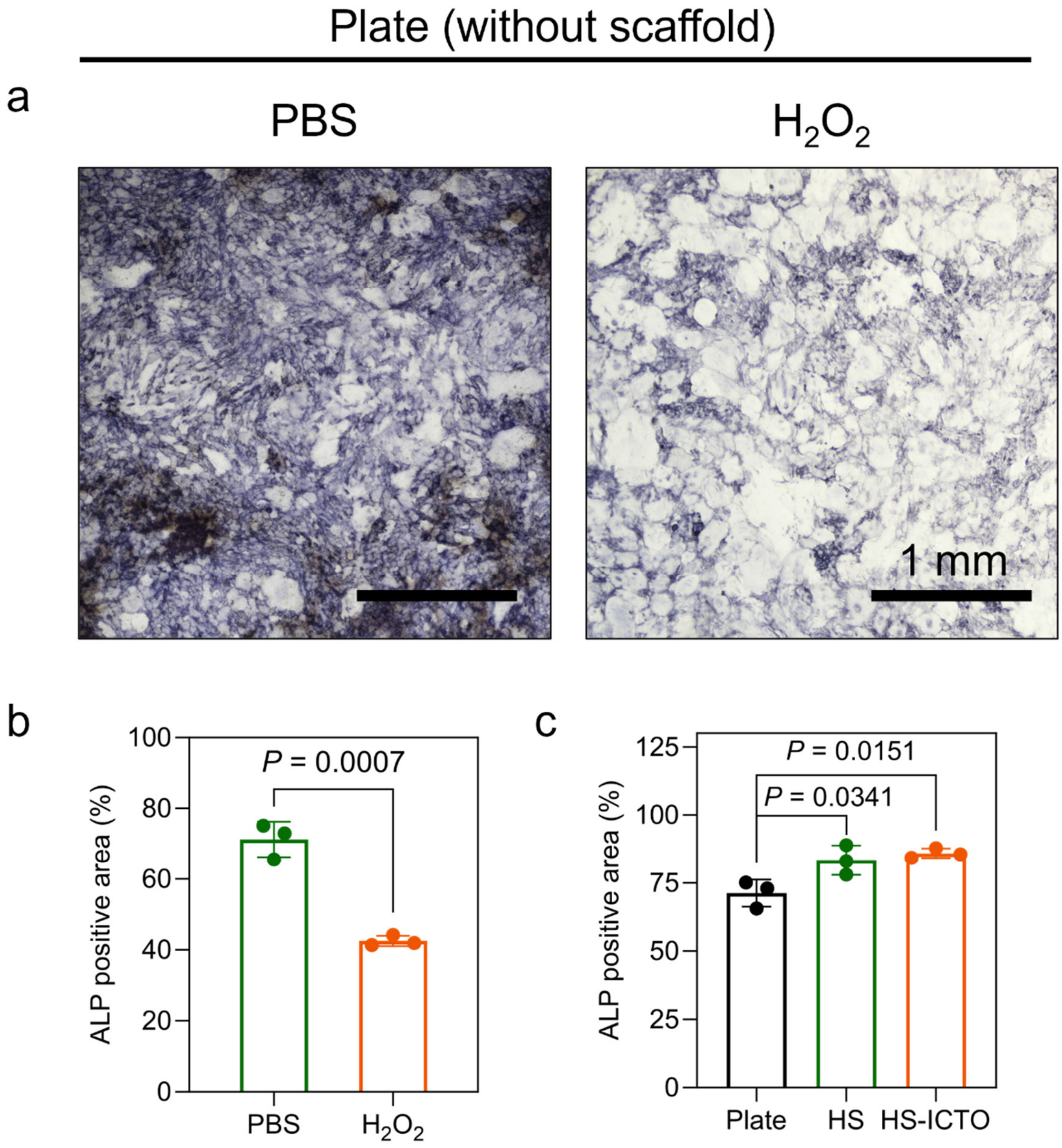

Supplementary Fig. 69. a ALP staining of the BMSCs stimulated by osteogenic medium for 14 days in the plate without scaffold. The images were representative of three independently repeated experiments from each group. **b** Quantitative analysis of the ALP positive areas. **c** Comparison of the ALP positive area in plate, HS, and HS-ICTO groups (n = 3 biologically independent replicates). Data are presented as mean ± SD, and ns represents no significant difference; in experiment (**b**), statistical significance was calculated using two-tailed Student's *t*-test and in experiment (**c**), statistical significance was calculated using one-way ANOVA followed by Tukey's post-hoc test for multiple comparisons; all tests were two-sided. Source data are provided as a Source Data file.

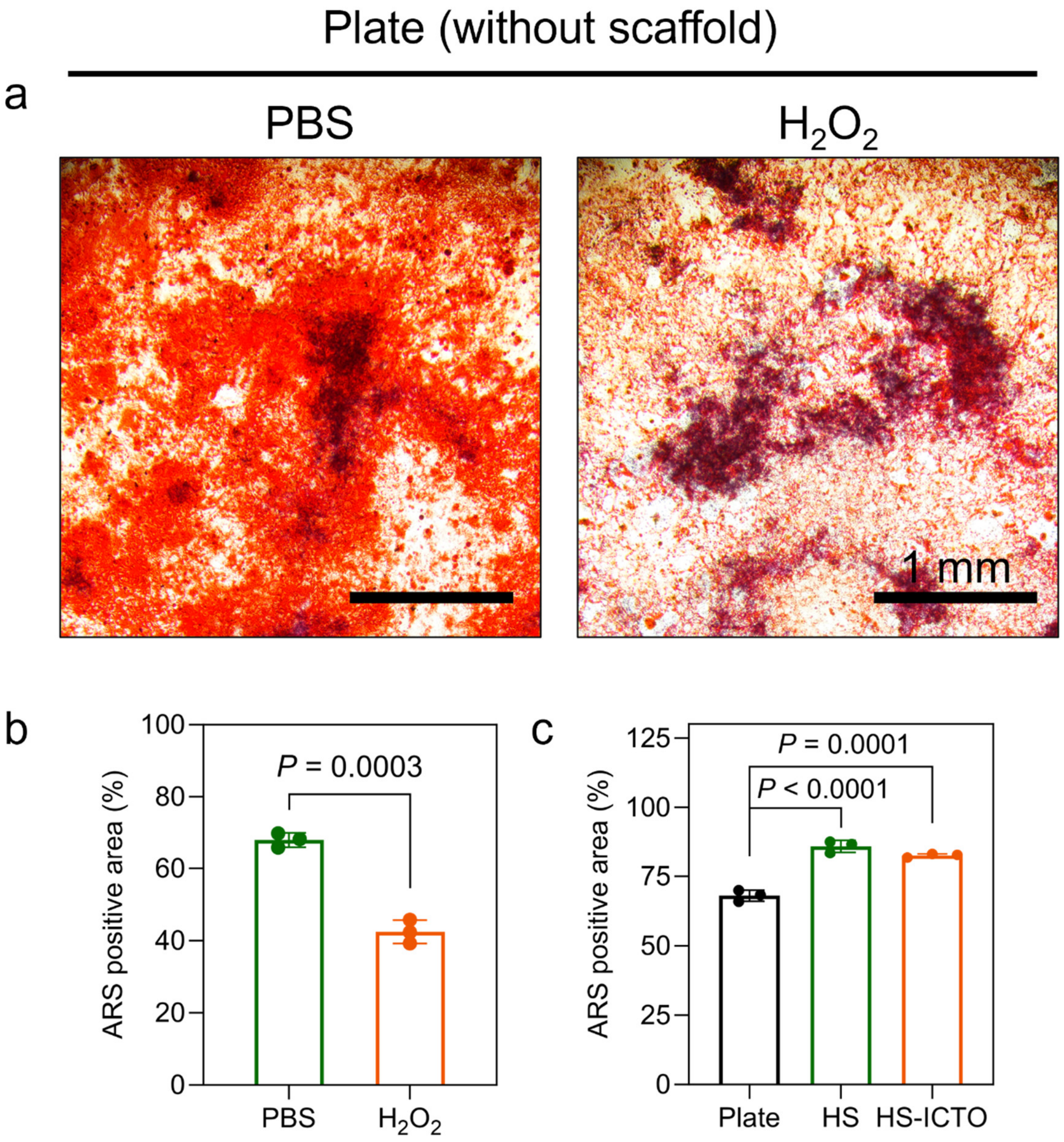

Supplementary Fig. 70. a ARS staining of the BMSCs stimulated by osteogenic medium for 21 days in the plate without scaffold. The images were representative of three independently repeated experiments from each group. **b** Quantitative analysis of the ARS positive areas (n = 3 biologically independent replicates). **c** Comparison of the ARS positive area in plate, HS, and HS-ICTO groups (n = 3 biologically independent replicates). Data are presented as mean ± SD, and ns represents no significant difference; in experiment (**b**), statistical significance was calculated using two-tailed Student's *t*-test and in experiment (**c**), statistical significance was calculated using one-way ANOVA followed by Tukey's post-hoc test for multiple comparisons; all tests were two-sided. Source data are provided as a Source Data file.

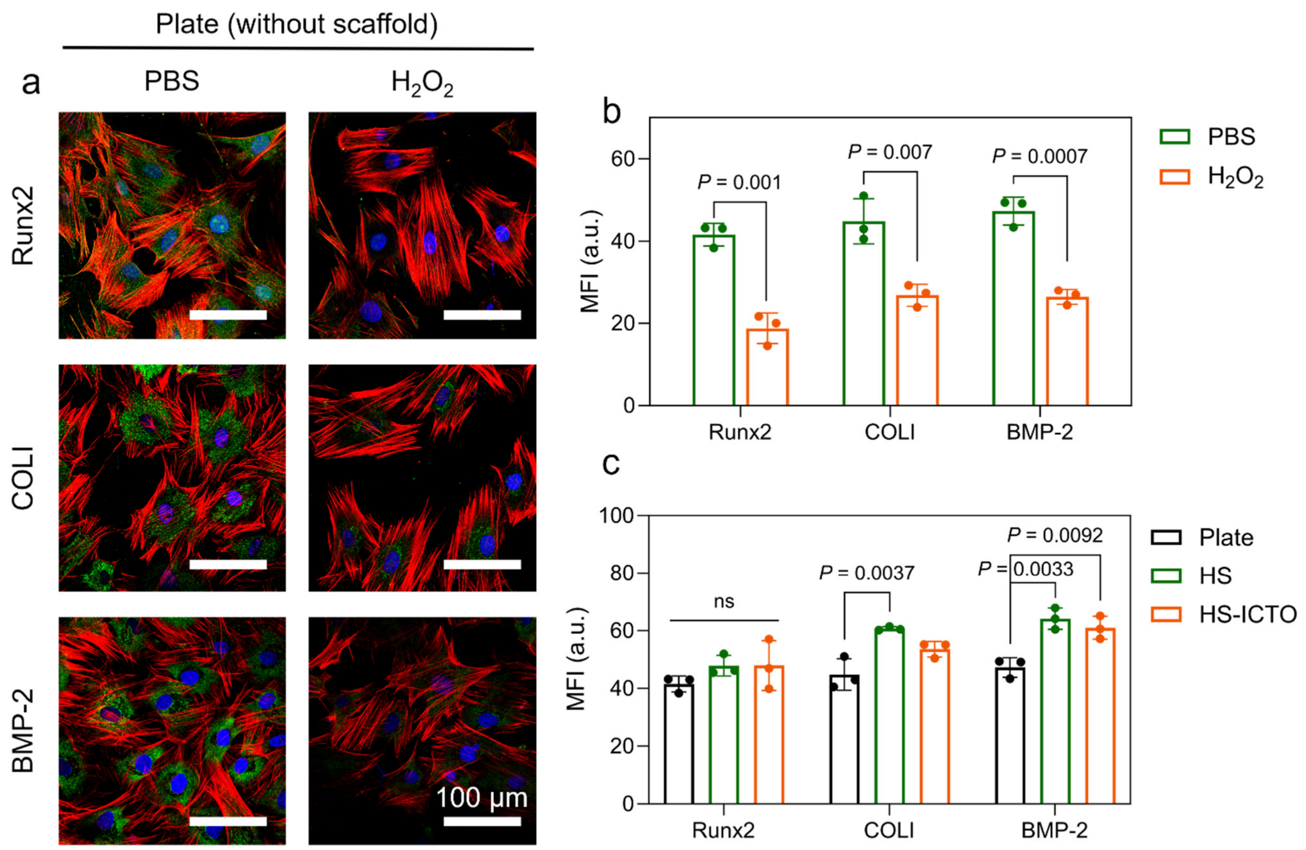

Supplementary Fig. 71. a Representative CLSM images of the Runx2, COLI, and BMP-2 immunofluorescence staining of BMSCs stimulated by osteogenic medium without scaffolds for 14 days. The images were representative of three independently repeated experiments from each group. **b** Quantitative analysis of the mean fluorescence intensity (MFI) in PBS and H_2O_2 groups (n = 3 biologically independent replicates). **c** Comparison of the MFI in plate, HS, and HS-ICTO groups (n = 3 biologically independent replicates). Data are presented as mean ± SD, and ns represents no significant difference; in experiment (**b**), statistical significance was calculated using two-tailed Student's *t*-test and in experiment (**c**), statistical significance was calculated using one-way ANOVA followed by Tukey's post-hoc test for multiple comparisons; all tests were two-sided. Source data are provided as a Source Data file.

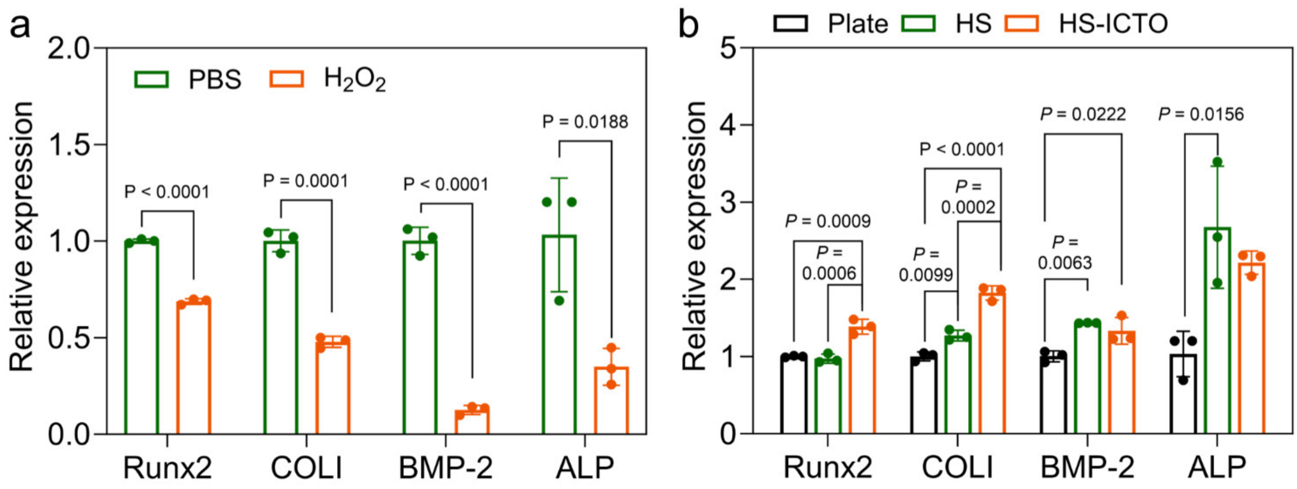

Supplementary Fig. 72. a The Runx2, COLI, BMP-2, and ALP mRNA expression of BMSCs after different stimulation without scaffolds for 14 days measured by RT-qPCR (n = 3 biologically independent replicates). **b** Comparison of the Runx2, COLI, BMP-2, and ALP mRNA expression of BMSCs in plate, HS, and HS-ICTO groups (n = 3 biologically independent replicates). Data are presented as mean ± SD, and ns represents no significant difference; in experiment (**a**), statistical significance was calculated using two-tailed Student's *t*-test, and in experiment (**b**), statistical significance was calculated using one-way ANOVA followed by Tukey's post-hoc test for multiple comparisons; all tests were two-sided. Source data are provided as a Source Data file.

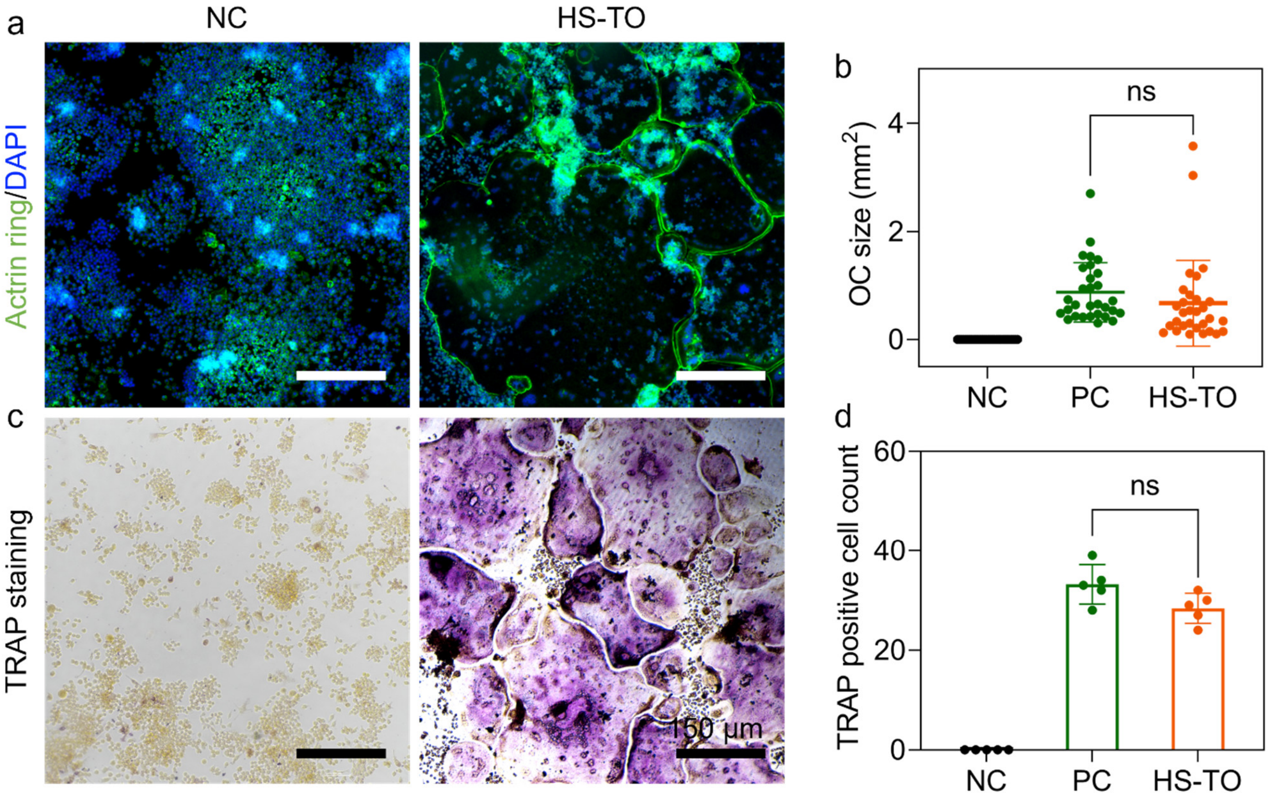

Supplementary Fig. 73. **a** Representative images of phalloidin-FITC stained RAW264.7 cells after negative control (NC, without RANKL stimulation) or HS-TO extracts treatment. **b** Quantitative analysis of the size of osteoclasts in NC, positive control (PC, with RANKL stimulation), and HS-TO extracts treatment (n = 30, random cells from each group were included). **c** Representative images of TRAP stained RAW264.7 cells after NC or HS-TO extracts treatment. **d** Quantitative analysis of the TRAP-positive cell counts in NC, PC, and HS-TO extracts treatment (n = 5 biologically independent replicates). The images in (**a**, **c**) were representative of three independently repeated experiments from each group. Data are presented as mean ± SD, and ns represents no significant difference; statistical significance was calculated using one-way ANOVA followed by Tukey's post-hoc test for multiple comparisons; all tests were two-sided. Source data are provided as a Source Data file.

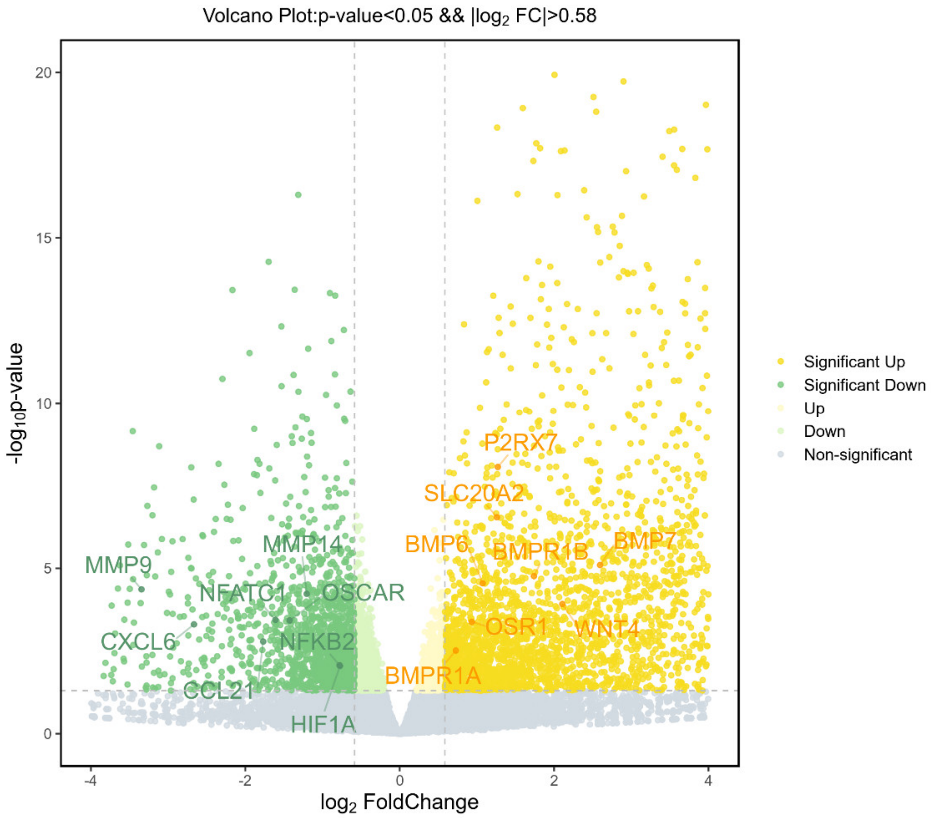

Supplementary Fig. 74. Volcano plot that demonstrates differential gene expression in HS-ICTO vs HS comparison from cranial defect rats at week 4 postoperatively. The data were representative of three biologically independent samples from each group. *P*-values was obtained from two-sided DESeq2 test without multiple comparison.

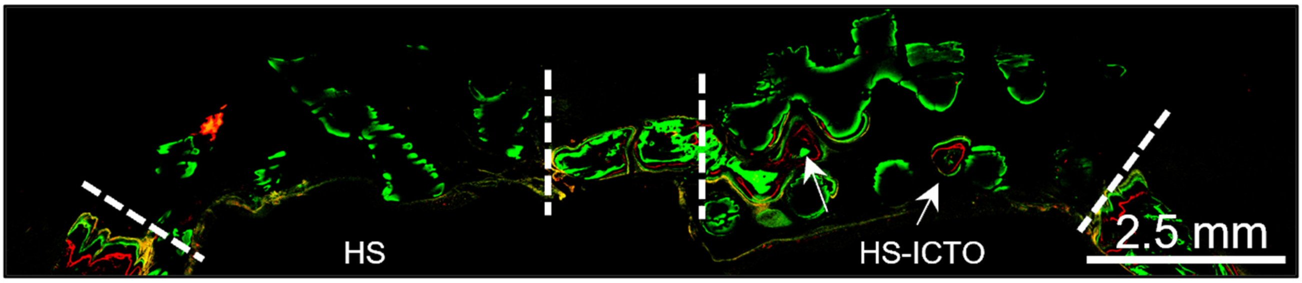

Supplementary Fig. 75. CLSM image of newborn osseous tissue with Alizarin red and Calcein-AM staining. The white dotted lines indicate the boundaries of the scaffolds. The white arrows indicate obvious ingrowth of the osseous tissue in HS-ICTO. The image was representative of three independently repeated experiments from each group.

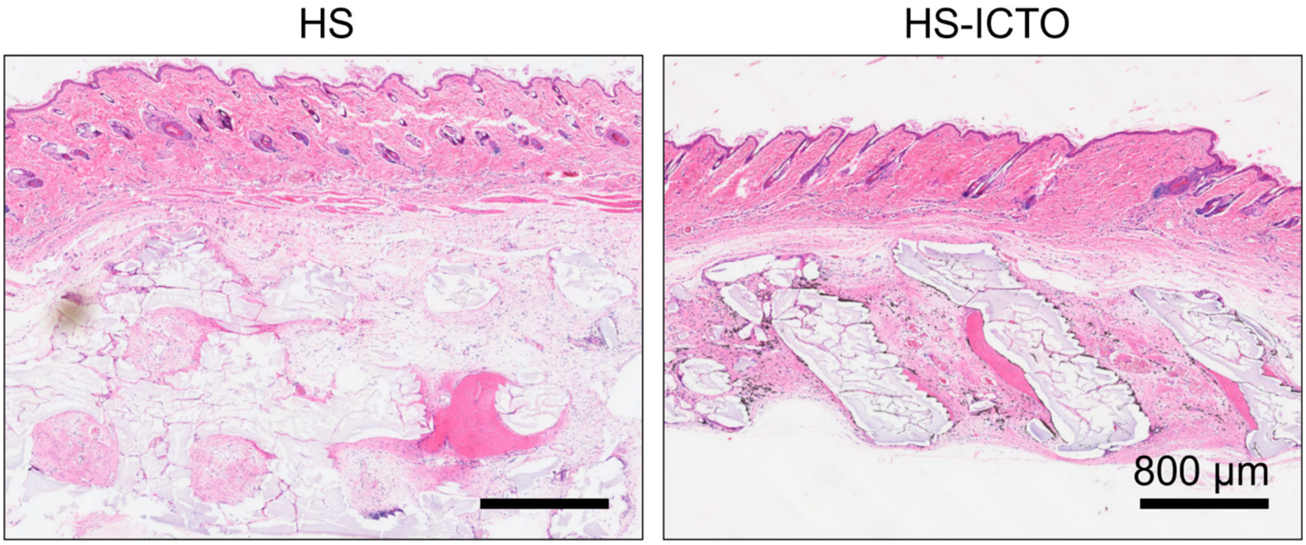

Supplementary Fig. 76. Representative H&E staining of HS and HS-ICTO implanted subcutaneously at week 8. The images were representative of three independently repeated experiments from each group.

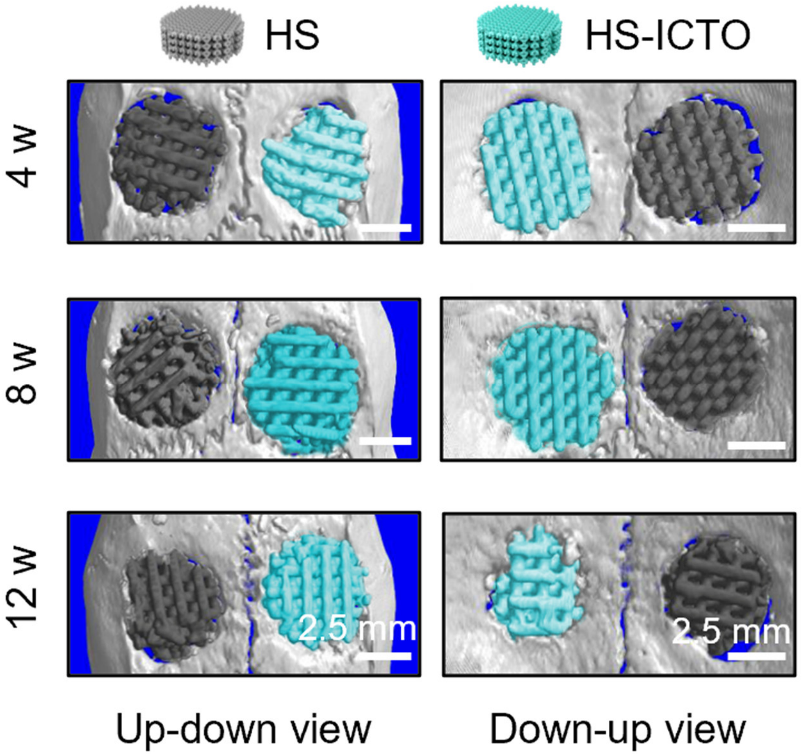

Supplementary Fig. 77. Representative 3D reconstruction of micro-CT images. The images were representative of three independently repeated experiments from each group.

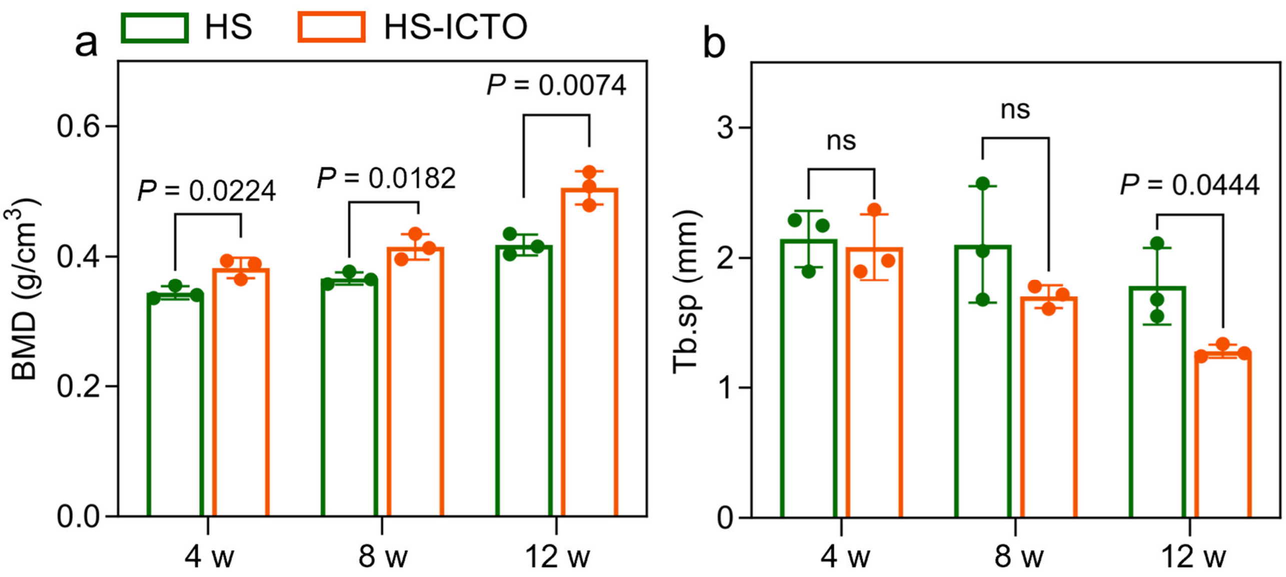

Supplementary Fig. 78. a Quantitative analysis of bone mineral density (BMD) and **b** trabecular separation (Tb.sp) induced by different scaffolds (n = 3 biologically independent replicates). Data are presented as mean ± SD, and ns represents no significant difference; statistical significance was calculated using two-tailed Student's *t*-test. Source data are provided as a Source Data file.

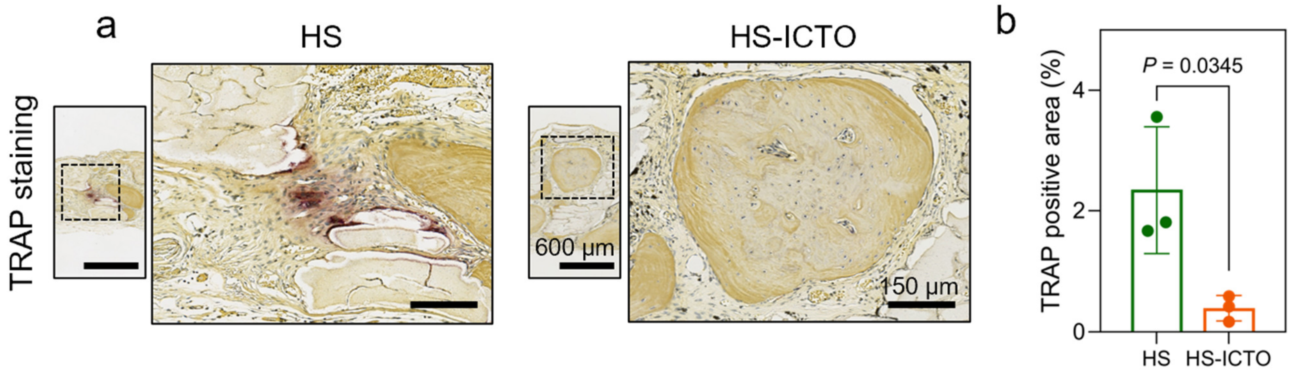

Supplementary Fig. 79. a Representative images of TRAP staining of different scaffolds harvested at week 12 and **b** the corresponding quantitative analysis of TRAP-positive areas (n = 3 biologically independent replicates). The images in (**a**) were representative of three independently repeated experiments from each group. Data are presented as mean ± SD, and ns represents no significant difference; statistical significance was calculated using two-tailed Student's *t*-test. Source data are provided as a Source Data file.

Supplementary Tables

Table S1. Kinetic constants of ICTO with H_2O_2 and TMB as substrates, respectively.

Samples	Substrate	K_m / mM	V_{max} /μM s^{-1}
ICTO	H_2O_2	0.655	0.351
ICTO	TMB	0.138	1.690

Table S2. Comparison of the kinetic constants of ICTO and other conventional ROS-catalytic materials with H_2O_2 as substrates for ROS production.

Catalysts	K_m (mM)	V_{max} (μM s^{-1})	TON (10^{-3} s^{-1})	Ref.
**ICTO**	**0.655**	**0.351**	**21.601**	**This work**
Co_3O_4	41.75	0.260	2.087	*Nat. Commun.* **10**, 704 (2019)[1].
CeO_2	4.41	0.180	3.096	*Nat. Commun.* **10**, 704 (2019)[1].
Fe_3O_4	41.66	0.160	1.237	*Nat. Commun.* **10**, 704 (2019)[1].
CuO	31.18	0.280	2.226	*Nat. Commun.* **10**, 704 (2019)[1].
Co-N-C	16.26	0.165	0.106	*ACS Catal.* **10**, 6422-6429 (2020)[2].
Cu_{NPs}/N-C	17.98	0.086	3.320	*Anal. Chem.* **92**, 3373-3379 (2020)[3].
Pt cube	1.00	0.002	0.257	*Adv. Funct. Mater.* **28**, 1801484 (2018)[4].
PtFe	217.60	0.082	2.199	*Angew. Chem. Int. Ed.* **131**, 12754-12761 (2019) [5].

Table S3. Comparison of the TON values of our synthesized catalysts with that of other reported metal oxides or metallic nanoparticles-based biocatalysts for O_2 generation.

Catalysts	K_m (mM)	V_{max} (uM s^{-1})	TON (s^{-1})	Ref.
**ICTO**	**273.37**	**48.23**	**4.1052**	**This work**
$Cu_{5.4}O$	0.07	3.92	0.2606	*Nat. Commun.* **11**, 2788 (2020)[6].
Co_3O_4 NPs	34.30	11.20	0.0450	*J. Mol. Catal. A: Chem.* **378**, 30-37 (2013)[7].
Co_3O_4 nanoplates	24.70	2.38	0.0095	*ACS Appl. Mater. Interfaces* **6**, 22216-22223 (2014)[8].
Co_3O_4 nanorods	4.82	1.88	0.0075	
Pd octahedrons	135.10	5.90	0.0251	*ACS Nano* **10**, 10436-10445 (2016)[9].
Mn_3O_4 cubes (MC)	0.44	5.30	0.0809	*Chem. Eur. J.* **24**, 8393-8403 (2018)[10].
Mn_3O_4 hexagonal plates (HPs)	0.37	7.37	0.1126	
IrO_x NPs	187.95	5.64	0.0108	*Angew. Chem. Int. Ed.* **59**, 9491-9497 (2020)[11].
$Au_{24}Cu_1$	292.00	5.83	1.9450	*Nat. Commun.* **12**, 114 (2021)[12].

Table S4. Primer sequence.

Species	Gene	Primer	Sequence (5’→3’)
Rat	*β-actin*	Forward	AGATCAAGATCATTGCTCCTCCT
		Reverse	ACGCAGCTCAGTAACAGTCC
	BMP-2	Forward	CCCCTATATGCTCGACCTGT
		Reverse	CGGGACGTTTTCCCACTCA
	Runx-2	Forward	ACAAATCCTCCCCAAGTGGC
		Reverse	GGATGAGGAATGCGCCCTAA
	ALP	Forward	TCCTTAGGGCCACCGCT
		Reverse	GGTGTACCCCGAGATCCGTT
	COL1A1	Forward	ACGCATGAGCCGAAGCTAAC
		Reverse	TCAGGTTTCCACGTCTCACC
Mouse	*β-actin*	Forward	TGCTGTCCCTGTATGCCTCTG
		Reverse	TGATGTCACGCACGATTTCC
	NFATc1	Forward	CCTTCAGAGAGACCTTGGC
		Reverse	CACAGGAGCTGGGGTTC
	c-Fos	Forward	GGGAATGGTGAAGACCGTGT
		Reverse	CCGTTCCCTTCGGATTCTCC
	MMP-9	Forward	CGACTTTTGTGGTCTTCCCC
		Reverse	CTTCTCTCCCATCATCTGGGC
	ACP5	Forward	TTACTACCGTTTGCGCTTC
		Reverse	CATTTTGGGCTGCTGACT

Supplementary Methods

Materials. Tetrabutyl orthotitanate (TBT), isopropanol (IPA), ethanol, N, N-dimethylformamide (DMF), 3,3',5,5'-tetramethylbenzidine (TMB), hydroethidine (HE), 9,10-diphenanthraquinone (DPA), 5,5'-Dithiobis-(2-nitrobenzoic acid) (DTNB), glutathione (GSH) 5,5-dimethyl-1-pyrroline N-oxide (DMPO), and 2,2,6,6-tetramethylpiperidine (TEMP) were purchased from Aladdin reagents (Shanghai, China), iridium (III) chloride hydrate ($IrCl_3 \cdot xH_2O$), and Titanium sulfate ($Ti(SO_4)_2$) was obtained from Energy Chemical (Shanghai, China). All other chemicals were used as received without further

purification, and deionized pure water (18.2 MΩ·cm) used in the experiments was produced from a Milli-Q Academic system (Millipore Corp., Billerica, MA, USA).

Characterizations. Field emission scanning electron microscopy (FE-SEM) was performed using the ThermoFisherScientific Apreo HiVoc instrument. The transmission electron microscopy (TEM) and aberration-corrected high-angle annular darkfield scanning TEM (AC HAADF-STEM) imaging, along with energy dispersive spectroscopy (EDS) mapping, were performed using a Talos F200x TEM microscope (FEI Ltd., USA) operated at 200 kV, and the data was analyzed using GMS-free analysis. The crystal structures of the catalysts were analyzed using X-ray diffraction (XRD) on a DX-2700BH (HaoYuan Instrument, China) using Cu Kα radiation over a 2θ range of 5−80°. X-ray photoelectron spectroscopy (XPS) spectra were measured using the *K*-Alpha™ + X-ray Photoelectron Spectrometer System (Thermo Scientific), equipped with a Hemispheric 180° dual-focus analyzer and a 128-channel detector. The X-ray absorption (XAS) spectra of the Ru *K*-edge were collected in fluorescence mode at the BL14W1 beamline of the Shanghai Synchrotron Radiation Facility, China, which was operated at 3.5 GeV with a maximum injection current of 230 mA. The synchrotron beam was monochromatized using a double-crystal monochromator with a Si (111) crystal to reduce the harmonic components of the monochromatic beam. Electron paramagnetic resonance (EPR) measurements were performed *via* the Bruker EPR EMX Plus (Bruker Beijing Science and Technology Ltd, USA) at a frequency of 9.8 GHz (microwave power: 1 mW). LAMBDA 1050+ Ultraviolet-Visible-Near-Infrared (UV-Vis-NIR) spectrophotometer (PerkinElmer, USA) was conducted for solution transmittance detection. Inductively coupled plasma mass spectrometry (ICP-MS) was performed using an Agilent 7850 ICP-MS system (Agilent Technologies, China). The mechanical properties of the scaffolds were evaluated using a universal mechanical testing system (68TM-30, INSTRON, USA). The piezoelectric properties were evaluated using an oscilloscope (RIGOL DS1102 Z-E, RIGOL Technologies, China) and atomic force microscopy (AFM, MFP-3D-BIO, Asylum Research, USA). Fluorescence images and bright-field images were collected via a confocal laser scanning microscope (N-SIM S, Nikon, Olympus, Japan) or inverted microscope systems (ECLIPSE Ti2, Nikon, Japan). CCK-8 results were collected using the Multifunctional Microplate Reader (SYNERGY H1, Bio-Tek, USA). Flow cytometry data were collected via a flow cytometer (FACSAriaIII, BD Biosciences, USA). RNA concentration and

purity were measured using NanoDrop 2000 (Thermo Fisher Scientific, Wilmington, DE, USA). RT-qPCR was performed using the real-time PCR system (QuantStudio® 3, Thermo Fisher Scientific, USA). Micro-CT images were collected using a micro-CT scanner (Quantum GX, PerkinElmer, USA). The libraries were sequenced on an Illumina NovaSeq 6000 platform. The pathology images were visualized using Vectra Polaris (PerkinElmer, USA). Data analysis was performed with various software, including GraphPad Prism Version 9.5, Origin 2024, VASP 5.4.1, MDI Jade 6, Avantage 5.9922, Athena software 0.9.26, Artemis software 0.9.26, Digital Micrograph 3.7.4, FlowJo Version 10.8.1, ImageJ Version 1.52v, and Qupath 0.4.0. Bioinformatics analyses were performed on the free online platform OECloud (https://cloud.oebiotech.com/). All original schematic diagrams were created using the open-source software Blender 3.4 and Inkscape 1.4.2, both distributed under the GNU General Public License (GPL).

Mechanical test. The mechanical properties of the scaffolds were evaluated using a universal mechanical testing system (68TM-30, INSTRON, USA). Cylindrical scaffold specimens (6 mm in diameter, 9 mm in height) were compressed at a constant displacement rate of 1 mm/min until visible fracture or mechanical failure (defined as a significant load drop) occurred.

Piezoelectric performance characterization. The piezoelectric properties were evaluated using an oscilloscope (RIGOL DS1102 Z-E, RIGOL Technologies, China) and atomic force microscopy (MFP-3D-BIO, Asylum Research, USA). For oscilloscope measurements, conductive silver paste was uniformly applied to the surfaces of dried HS and compressed HA powder specimens, followed by tin foil overlay. The assemblies were cured at 60°C for 30 minutes to solidify the conductive silver paste. Copper wires were attached to both sides of the tin foil as output leads and encapsulated with polyvinyl chloride electrical insulation tape. The resultant piezoelectric sensors were connected to the oscilloscope to obtain output voltage waveforms under US stimulation (US parameters: 1 W/cm^2, 1.0 MHz, 30% duty cycle). For atomic force microscopy analysis, specimens were fabricated into standardized circular discs with 5 mm diameter and 1mm thickness. Alternating electric fields were applied to three randomly selected non-edge regions per sample group to detect surface

nanodeformation, thereby validating their nanoscale piezoelectric response. Each position was measured in triplicate to minimize local heterogeneity effects.

ICTO nanoparticle release experiments. Release kinetics study of ICTO nanoparticles was conducted using UV-Vis-NIR spectroscopy to evaluate the transmittance of solutions at various immersion time points. The prepared HS-ICTO scaffolds were immersed in 4 mL PBS buffer, and the immersion solutions were collected at predetermined time intervals (7, 14, 21, 28-day). The transmittance of the solutions was measured using UV-Vis-NIR spectroscopy. The release quantity of ICTO nanoparticles was calculated using pure PBS as a reference.

Inductively coupled plasma mass spectrometry (ICP-MS) detection. The quantitative assessment of metal ion release kinetics was conducted using ICP-MS. Degradation studies were performed by immersing HS-ICTO scaffolds in PBS solutions at physiological and mildly acidic conditions (pH 7.4 and 6.5, respectively). The temporal evolution of metal ion release from HS-ICTO scaffold was systematically monitored at 7, 14, 21, and 28-day intervals. Supernatant analysis was performed using an Agilent 7850 ICP-MS system to characterize ionic species composition and parameters. The mass spectrometric analysis was optimized under the following operational parameters: RF power (1600 W), RF matching (1.8 V), peristaltic pump velocity (0.1 rps), nebulizer gas flow rate (1.05 L/min), auxiliary gas flow rate (0.9 L/min), plasma gas flow rate (15 L/min), sampling depth (10 mm), and nebulization chamber temperature (2°C). Mass spectral acquisition was conducted with a sampling period of 0.31 s and an integration time of 0.1 s. Elemental concentrations were determined directly in mg/L using the calibrated instrument. Data acquisition and thermal measurements were performed using Origin 2022 software within a temperature range of 0.5°C, with subsequent data processing conducted using Origin 2024.

Peroxidase (POD)-like activity measured by TMB. The POD-like activity of the catalysts was determined using colorimetric assays. In a 2 mL sodium acetate-acetic acid (NaOAc/HOAc) buffer (100 mM, pH 4.5), 15 μL of the catalysts (4 mg/mL), 25 μL of TMB (10 mg/mL), and 25 μL of H_2O_2 (0.1 M) were added. The catalytic oxidation of TMB was studied by measuring the absorption changes

of the oxidized form of TMB at λ_{max} = 652 nm (ε = 39,000 M^{-1} cm^{-1}). Unless otherwise noted, the POD-like activities were performed in an air-saturated buffer. The catalytic activity assays under varying pH conditions were conducted by substituting buffer solutions of different pH values. The POD-like activity of the HS-ICTO scaffold was then operated as above, replacing only the catalyst with the HS-ICTO scaffold.

Oxidase (OXD)-like activity measured by TMB. The OXD-like activity of the catalysts was determined using colorimetric assays. In a 2 mL sodium acetate-acetic acid (NaOAc/HOAc) buffer (100 mM, pH 4.5), 25 μL of the catalysts (at a concentration of 4 mg/mL), and 25 μL of TMB (10 mg/mL) were added. The catalytic oxidation of TMB was studied by measuring the absorption changes of the oxidized form of TMB at λ_{max} = 652 nm (ε = 39,000 M^{-1} cm^{-1}). Unless otherwise noted, the OXD-like activities were performed in an air-saturated buffer. The catalytic activity assays under varying pH conditions were conducted by substituting buffer solutions of different pH values.

Enzyme dynamic parameters. The Michaelis-Menten constant was determined from the Michaelis-Menten saturation curve. The initial reaction rates (*V*) were calculated from the absorbance variation using the Beer-Lambert Law (Equation 1), where the absorbance changes of the oxidized form of TMB (ε = 39,000 M^{-1} cm^{-1}) were used to determine the concentration of ox-TMB (c) and the length of the solution in the light path (l cm). The reaction rates were plotted against their corresponding H_2O_2 or TMB concentrations and fitted with the Michaelis-Menten curves (Equation 2). Additionally, a Lineweaver-Burk plot (Equation 3) was used to determine the maximum reaction velocity (V_{max}) and Michaelis constant (K_m) from the linear double-reciprocal plot. The turnover number (TON) was also calculated using Equation 4.

$$A=\varepsilon lc \quad (1)$$

$$V = (V_{max}\times[S]) / (K_m + [S]) \quad (2)$$

$$1/V = K_m / (V_{max}\times[S]) + (1/V_{max}) \quad (3)$$

$$TON = V_{max}/[E_0] \quad (4)$$

[S] is the concentration of H_2O_2 or TMB, and [E_0] is the molar concentration of metal in biocatalysts.

GSH Depleting with ICTO. The depletion of GSH by a catalyst was detected using the characteristic colorimetric reaction of DTNB and GSH. This reaction results in the formation of a yellow-colored product with a characteristic absorption peak at 412 nm, while DTNB has negligible absorption above 400 nm. Specifically, a mixture of 10, 20, 50, and 100 μL of ICTO (4 mg/mL) was combined with 30 μL of 10 mM GSH in PBS solution at room temperature. After stirring for 60 minutes, 240 μL of 3 mg/mL DTNB was added to detect the sulfydryl (-SH) group in GSH. The catalytic degradation of GSH was monitored by measuring the change in absorbance at 412 nm. The GSH depleting efficiency of the HS-ICTO scaffold was then operated as above, replacing only the catalyst with the HS-ICTO scaffold.

Detection of superoxide anions (•O_2^-) by HE. The HE probe was utilized to measure the levels of •O_2^- in a system, which could react with •O_2^- to produce fluorescent Ethidium at the wavelength of 470 nm and an emission wavelength of 610 nm. The experimental procedure involved mixing 1.5 mL of a catalyst solution (concentration of 100 μg/mL) with 1.5 μL of 0.1 M H_2O_2 at 37 °C for 40 minutes. Subsequently, 1.5 mL of a HE-ethanol solution (concentration of 1 mg/mL) was added to the system and thoroughly vortexed. The solution was allowed to sit undisturbed for 40 minutes before being subjected to fluorescence measurements using a Synergy Mx device. The catalytic activity of the HS-ICTO scaffold was then operated as above, replacing only the catalyst with the HS-ICTO scaffold.

Detection of singlet oxygen ($^1O_2$) by DPA. In the detection of $^1O_2$, a mixture was prepared by adding 25 μL of a 4 mg/mL catalyst solution, 25 μL of a 0.1 M H_2O_2 solution, and 100 μL of a DPA-DMSO solution into a 2 mL DMSO solution. The mixture was then analyzed using a UV-vis spectrophotometer. The catalytic activity of the HS-ICTO scaffold was then operated as above, replacing only the catalyst with the HS-ICTO scaffold.

EPR measurement. The generation of •O_2^- was evaluated by EPR spectrometer using DMPO spin-trapping adduct in DMSO solvent. 10 μL of biocatalysts (10 mg/mL, DMSO) and 10 μL of H_2O_2 (10 M) were added into a 0.5 mL DMSO, and then 10 μL DMPO was added. The generation of $^1O_2$ was

evaluated by EPR spectrometer using TEMP spin-trapping adduct in NaOAc-HOAc buffer (100 mM, pH 4.5). 10 μL of biocatalysts (10 mg/mL, NaOAc-HOAc buffer) and 10 μL of H_2O_2 (10 M) were added into a 0.5 mL buffer, and then 10 μL TEMP was added. Detection of US-enhanced $\bullet O_2^-$ and $^1O_2$ production was achieved by applying US irradiation (Nu-Tek UT1041, 2.0 W/cm^2, 1 MHz, 30% duty cycle, 1 min).

Sono-activation performance of biocatalyst detected by DPA. A mixture was prepared by adding 25 μL of a 4 mg/mL catalyst solution and 100 μL of a DPA-DMSO solution into a 2 mL DMSO solution. The mixture was then analyzed using a UV-vis spectrophotometer. The decomposition rate of DPA by catalyst was measured after different durations of US irradiation (Nu-Tek UT1041, 2.0 W/cm^2, 1 MHz, 30% duty cycle). The relative absorbance changes of DPA at 378 nm were used to quantify the decomposition rate.

US-enhanced POD-like activity. In a 2 mL sodium acetate-acetic acid (NaOAc/HOAc) buffer (100 mM, pH 4.5), 15 μL of the catalysts (at a concentration of 4 mg/mL), 25 μL of TMB (10 mg/mL), and 10 μL of H_2O_2 (0.1 M) were added, and US was applied (Nu-Tek UT1041, 2.0 W/cm^2, 1 MHz, 30% duty cycle). The catalytic oxidation of TMB enhanced by the US was studied by measuring the absorption changes of the oxidized form of TMB at λ_{max} = 652 nm.

US-enhanced OXD-like activity. In a 2 mL sodium acetate-acetic acid (NaOAc/HOAc) buffer (100 mM, pH 4.5), 15 μL of the catalysts (at a concentration of 4 mg/mL), and 25 μL of TMB (10 mg/mL) were added, and US was applied (Nu-Tek UT1041, 2.0 W/cm^2, 1 MHz, 30% duty cycle). The catalytic oxidation of TMB enhanced by the US was studied by measuring the absorption changes of the oxidized form of TMB at λ_{max} = 652 nm.

The catalase (CAT)-like activity measurement. The CAT-like activity was evaluated by measuring H_2O_2 scavenging and O_2 generation. To assess the H_2O_2 conversation capacity, 20 μL of 1 M H_2O_2, 1.97 mL of PBS (pH 7.4 or 6.5), and 10 μL of ICTO (10 mg/mL) were mixed, and after 30 minutes, 100 μL of the solution was combined with 100 μL of a $Ti(SO_4)_2$ solution (13.9 mM). The absorption

intensity at 405 nm was then measured using a microplate reader. To determine the O_2 generation capability, 200 μL of 10 M H_2O_2 was added to 20 mL of PBS (pH 7.4 or pH 6.5) containing 20 μL of ICTO (10 mg/mL), and the O_2 concentration was detected using a Dissolved Oxygen Meter over a period of 5 minutes with 5-second intervals.

The steady-state kinetic assays of catalase-like activities were evaluated by changing the concentration of H_2O_2. The assays were conducted in 20 mL centrifuge tubes containing 20 mL of PBS (pH 7.4) and 20 μL of ICTO (10 mg/mL). In a typical assay, different concentrations of H_2O_2 (50-1200 mM) were mixed with 20 mL of PBS containing ICTO to monitor the solubility change of O_2 for 5 minutes using a Dissolved Oxygen Meter. For each H_2O_2 concentration, the "Absorbance versus Time" curve was obtained, which was used to calculate the initial reaction velocity (V). The reaction rates were then plotted against their corresponding H_2O_2 concentration (S) and fitted with Michaelis-Menten curves. The slope and intercept of the linear double reciprocal plot were used to determine the maximum reaction velocity (V_{max}) and Michaelis-Menten constant (K_m). Additionally, the catalytic efficiency in terms of turnover number (TON) was calculated, where [E_0] represents the concentration of the catalytic active center of ICTO. The TON/K_m value reflects the catalytic efficiency of ICTO.

Theoretical Calculation. All theoretical calculations were performed using the DFT method, as implemented in the Vienna ab initio simulation package (VASP)[13-15]. The core electrons were described using the spin-polarized projector augmented wave (PAW) method[16], and the electron exchange and correlation energy were treated within the generalized gradient approximation in the Perdew-Burke-Ernzerhof functional (GGA-PBE)[17]. The valence states of all atoms were expanded in a plane-wave basis set with a cutoff energy of 450 eV. The convergence criteria for the electronic self-consistent iteration and force were set to 10^{-5} eV and 0.02 eV/Å with a Gamma centered 1 × 2 × 1 K-points. Denser 3 × 4 × 1 K-points were used for the density of states (DOS) computations. Slab model was constructed with a vacuum layer of 18 Å in the z direction to avoid the interaction between neighboring images. The charge density differences were evaluated using the formula (5).

$$\Delta\rho = \rho_{A+B} - \rho_{A} - \rho_{B} \qquad (5)$$

where ρ_X is the electron density of X. Atomic charges were computed using the atom-in-molecule (AIM) scheme proposed by Bader. The isosurface value used for differential charge density is 0.01 e·Bohr^{-3}.

To explore the catalytic effect, the change of Gibbs free energy (ΔG) was calculated, which is defined as:

$$\Delta G = \Delta E + \Delta ZPE + \Delta H_{0\rightarrow 298K} - T\Delta S \quad (6)$$

where ΔE is the energy change obtained from DFT calculations; ΔZPE, ΔH, and ΔS denote the difference in zero-point energy, enthalpy, and entropy due to the reaction, respectively. The enthalpy and entropy of the ideal gas molecules were taken from the standard thermodynamic tables, and some of the calculation results were analyzed using the VASPKIT package[18].

Cytotoxicity assays and the intracellular ROS generation and GSH depletion ability of ICTO. The 143b cells (cultured with RPMI-1640 medium from Gibco containing 10% fetal bovine serum and 100 U/mL penicillin-streptomycin) and BMSCs (cultured with α-MEM medium from Gibco containing 10% fetal bovine serum and 100 U/mL penicillin-streptomycin) were inoculated in 96 wells at a density of 1 × 10^4/well and then incubated with a serial concentration gradient of ICTO and TO for 24 h. Hydrochloric acid was added to modify the pH of the conditioning medium. After that, the viabilities of cells were evaluated by Cell Counting Kit-8 (CCK-8, C0038, Beyotime). The OD_{450} was recorded using a microplate reader. For intracellular ROS detection, 143b cells were inoculated in 96 wells at a density of 8 × 10^3/well and then incubated with 100 μg/mL ICTO or TO for 12 h. After that, 2′,7′-dichlorodihydrofluorescein diacetate (DCFH-DA, D6883, Sigma-Aldrich, 20 μM) ROS probe was added and US irradiation (Nu-Tek UT1041, 1 W/cm^2, 1 MHz, 30% duty cycle, 1 min) was applied to the corresponding group. After washing with PBS, the intracellular fluorescence was monitored using an inverted fluorescence microscope (ECLIPSE Ti2, Nikon). For intracellular GSH detection, the ThiolTracker Violet GSH probe (T10095, Invitrogen™) was used to label the intracellular GSH after similar treatments, and the nuclei were stained by Hoechst 33342 (C0031, Solarbio). The MFI of DCFH-DA and GSH staining were calculated using Image J software.

RNA sequencing (RNA-seq) and differentially expressed genes analysis. In assessing SDT and CDT tumoricidal efficacy, three distinct experimental groups were established: the HS, HS-ICTO, and the HS-ICTO + US groups. The 143b cell was inoculated onto sterilized HS-ICTO or HS, as mentioned in the manuscript. The HS-ICTO + US group was subjected to US irradiation. Total RNA extraction was performed using the TRIzol reagent (Invitrogen, California, USA) in accordance with the manufacturer's protocol. Subsequently, RNA-seq libraries were constructed with the VAHTS Universal V6 RNA-seq Library Prep Kit and sequenced on the Illumina Novaseq 6000 platform. The transcriptome sequencing and subsequent data analysis were carried out by OE Biotech Co., Ltd. (Shanghai, China). Differential expression analysis was conducted employing the DESeq2, with significantly differentially expressed genes (DEGs) identified using a threshold of p-value < 0.05 and fold change > 3/2 or < 2/3. Principal Component Analysis (PCA) was executed using R (version 3.2.0) to evaluate the biological variance among replicates. Volcano plots and heat maps were generated using GraphPad Prism (version 9.5) or the OECloud tools at https://cloud.oebiotech.com. For functional annotation, the Database for Annotation, Visualization, and Integrated Discovery (DAVID, https://david.ncifcrf.gov/) was utilized to perform Gene Ontology (GO) term enrichment analysis, thereby elucidating the functional distinctions among the DEGs related to biological processes.

Flow cytometry for cell apoptosis analysis. 143b cells were seeded onto HS-ICTO or HS in 48-well plates with a similar treatment mentioned in the manuscript. The cells on the scaffolds were trypsinized and collected along with the cells in the supernatant. The Annexin V-FITC/PI Apoptosis Detection Kit (FXP018, 4A Biotech) was applied to stain the cells. After staining buffer washing, the sample was analyzed by flow cytometry (FACSAriaIII).

TEM observation of the cellular structure. After seeding and corresponding treatments, the cells on the scaffolds were trypsinized and collected along with the cells in the supernatant. After 300 × g centrifugation for 3 min, the cell pellets were resuspended with 1 : 5 (3 % glutaraldehyde: PBS) fixing solution and incubated in 4 °C for 5 min. After another 300 × g centrifugation for 3 min, the cell pellets were immersed with 3 % glutaraldehyde before TEM observation. Similar procedures were performed for the TEM observation of BMSCs.

Intracellular ROS detection of cells on HS-ICTO. 143b cells were seeded onto HS-ICTO or HS and allowed to adhere for 12 h. DCFH-DA were added following US irradiation. After 30 min, PBS washing was performed, and the intracellular ROS was observed using an inverted fluorescence microscope (ECLIPSE Ti2, Nikon). As for the ROS scavenging detection, BMSCs were seeded onto HS-ICTO or HS in 48-well plates at a density of 1×10^5 cells per scaffold and allowed to adhere for 12 h. Then, the ROS-conditioning medium (complete medium containing 100 μM H_2O_2) was added. An identical volume of PBS was added to the cultural medium as the control group. Similar DCFH-DA labeling (without US irradiation) and observation procedures were performed. The integrated intensity was calculated using ImageJ software.

Mitochondria membrane potential evaluation. Briefly, 143b cells were seeded in 96 well plates at a density of 8×10^3 cells per well for attaching overnight. Then, medium with HS-ICTO or HS extracts was added (1 mL complete medium per scaffold and incubated under 37 °C and 60 rpm for 48 h. Then the scaffold was discarded, and the medium was adopted). After incubation for 12 h, US irradiations were applied to the corresponding well. The mediums were removed following PBS washing. Then, the JC-1 mitochondria membrane potential probe (C2006, Beyotime) was added and incubated for 20 min at 37 °C. After PBS washing, the mitochondria membrane potential was visualized using an inverted fluorescence microscope. The MFIs were calculated using ImageJ software.

Evaluation of hypoxia relief by HS-ICTO. In brief, 143b cells were seeded onto HS-ICTO or HS. Then, for hypoxia stimulation, the corresponding plates were placed into a hypoxia incubator containing 1% O_2, 5% CO_2, and 94% N_2. After 24 h, the cells were trypsinized and collected. The protein expression of HIF-1α (1:1000, ab216842, Abcam) and GAPDH (1:1000, T0004, Affinity) was evaluated by western blot assay. The relative protein expression was calculated using ImageJ software.

Hemolysis evaluation. Whole blood was first diluted to a 2% concentration with normal saline and aliquoted into 1.5 mL EP tubes. Free TO or ICTO nanoparticles were then added. Normal saline was used for the negative control, while ultrapure water served as the positive control in place of

nanoparticles. All samples were incubated at 37 °C for 2 hours, followed by centrifugation at 1000 × g for 5 minutes. The supernatant's absorbance at 540 nm was measured using a microplate reader. The hemolysis rate was determined using the formula: hemolysis rate = [(Ax – An)/(Ap − An)] × 100%, where Ax, An, and Ap denote the absorbance values of the treated samples, negative control, and positive control, respectively.

Blood routine test and biochemical test. The HS and HS-ICTO were subcutaneously implanted in the dorsal region of 8-week-old male SD rats. Peripheral blood was collected after 4 weeks for comprehensive hematological and biochemical assessments of liver and kidney functions. The blood routine results were collected using the Hematology Analyzer (BC-2800Vet, Mindray, China), and the biochemical results were collected using the Biochemical Analyzer (BS-460, Mindray, China).

Histological staining for tumor and organs. The tumor and organs were fixed with 10% neutral formalin for 48 hours immediately after dissection. They were then dehydrated, transparent, and dipped in wax in an automatic dehydrator (EXCELSIOR AS, Thermos) and then embedded in paraffin (HISTOSTAR, Thermos). The continuous sliced histological sections were prepared using HM 340E (Thermos) with a thickness of 4 μm. A standard procedure for staining the soft tissue with H&E was performed to demonstrate the general histological structure of the tissues. The heart, liver, spleen, lung, and kidney were also observed by H&E staining to evaluate the biocompatibility. The Ki67, HIF-1α, and cleaved caspase-3 expression were demonstrated by immunofluorescence staining. Briefly, after the antigen retrieval, the slices were blocked and then incubated with 100 μL anti-Ki67 (1 : 200, 12202, CST) or anti-HIF-1α (1 : 50, NB100-105, Novus) or anti-cleaved caspase-3 (1 : 200, 9664, CST) working solution. After PBS washing twice, the secondary antibody was added and incubated in the dark for 45 min, 37 °C. Finally, after PBS washing, DAPI staining, and antifade solution seal, the slices were scanned by Vectra Polaris (PerkinElmer), and the images were analyzed by Qupath software.

***In vitro* evaluation of BMSCs viability protection against ROS attack of HS-ICTO.** In short, BMSCs were seeded onto HS-ICTO or HS. Then, the control or ROS-conditioning medium was added. Calcium-AM staining and CCK-8 assay were applied to demonstrate the cellular viability and live cell

counts on days 1, 3, and 5. The live cells were visualized using CLSM and calculated using Image J software.

Observation of BMSCs morphology on scaffolds. BMSCs were seeded onto HS-ICTO or HS following the same control or ROS-conditioning medium treatment. After 24 h, the cells were fixed with 4% paraformaldehyde for 15 minutes at room temperature. The cytoskeletal staining was carried out by Actin-Tracker Red-Rhodamine (1:200, C2207S, Beyotime) and DAPI staining followed by CLSM observation. The cell size was measured using Image J software.

Evaluating the *in vitro* osteogenic expression of BMSCs. Immunofluorescence staining and RT-qPCR were applied to demonstrate the osteogenic protein and mRNA expression. For immunofluorescence staining, in short, BMSCs were seeded onto HS-ICTO or HS in 48-well plates at a density of 1×10^5 cells per scaffold and allowed to adhere for 12 h. Then, the cells were cultured with ROS-conditioning or control medium for 24 h. Next, the mediums were replaced with an osteogenic medium and refreshed every third day. On day 14, the cells were trypsinized and re-inoculated into confocal dishes and allowed to adhere for 12 h. Then, the BMSCs were washed twice with sterile PBS and fixed with 4% paraformaldehyde. Then 0.5% Triton X-100 was used to permeabilize the membrane for 10 minutes at room temperature. After PBS wash twice, 5% BSA was applied for blocking. The anti-BMP-2 antibody (Abcam, ab214821, 1:200), anti-Runx2 antibody (Proteintech, 20700-1-AP, 1:200), and anti-collagen-I antibody (Proteintech, 14695-1-AP, 1:200) were added for overnight incubation under 4 °C. After PBS washing, the FITC–conjugated goat anti-rabbit IgG (Proteintech, SA00003-2, 1:1000) was added for 1 h incubation under 37 °C in the dark. After PBS washing, the Actin-Tracker Red-Rhodamine (1:200, C2207S, Beyotime) was added to stain the cytoskeleton. Then, DAPI was added to label the nucleus for 5 min. After gentle PBS washing twice, the immunofluorescence staining was observed and recorded using CLSM. The mean fluorescence intensity was calculated using ImageJ software.

For RT-qPCR, after the same treatment, the total mRNA was extracted (LS1040, Promega). After reverse transcription (11141ES60, YEASEN), the RT-qPCR was performed using Hieff UNICON® Universal Blue qPCR SYBR Green Master Mix (11184ES08, YEASEN) in QUANTSTUDIO3, Applied Biosystems. Primer sequences are shown in Table S4.

Migration assay. The transwell system (8 μm pore size)was utilized to assess the migration ability of BMSCs. Briefly, BMSCs were seeded in the upper chamber of the transwell at a density of 3×10^4 cells/well, and basal medium was added. In the lower chamber, a complete medium with or without 100 μM H_2O_2 was added, and either HS or HS-ICTO scaffolds were placed. After 12 h of incubation, the transwell chambers were removed. The cells were fixed with formaldehyde and stained with crystal violet. Non-migrated cells on the upper surface of the membrane were gently removed using a cotton swab. The chambers were then placed on glass slides, and images were captured using an inverted microscope. The crystal violet-positive stained area was quantified using ImageJ to evaluate the number of migrated cells.

References

1 Wang, X. et al. eg occupancy as an effective descriptor for the catalytic activity of perovskite oxide-based peroxidase mimics. *Nat. Commun.* **10**, 704 (2019).

2 Jiao, L. et al. Densely isolated FeN4 sites for peroxidase mimicking. *ACS Catal.* **10**, 6422-6429 (2020).

3 Wu, Y. et al. Cascade reaction system integrating single-atom nanozymes with abundant Cu sites for enhanced biosensing. *Anal. Chem.* **92**, 3373-3379 (2020).

4 Ge, C. et al. Synthesis of Pt hollow nanodendrites with enhanced peroxidase-like activity against bacterial infections: implication for wound healing. *Adv. Funct. Mater.* **28**, 1801484 (2018).

5 Li, S. et al. A nanozyme with photo-enhanced dual enzyme-like activities for deep pancreatic cancer therapy. *Angew. Chem. Int. Ed.* **131**, 12754-12761 (2019).

6 Liu, T. et al. Ultrasmall copper-based nanoparticles for reactive oxygen species scavenging and alleviation of inflammation related diseases. *Nat. Commun.* **11**, 2788 (2020).

7 Mu, J. et al. Co3O4 nanoparticles as an efficient catalase mimic: Properties, mechanism and its electrocatalytic sensing application for hydrogen peroxide. *J. Mol. Catal. A: Chem.* **378**, 30-37 (2013).

8 Wang, F. et al. High-performance ZnCo2O4@ CeO2 core@ shell microspheres for catalytic CO oxidation. *ACS Appl. Mater. Interfaces* **6**, 22216-22223 (2014).

9 Ge, C. et al. Facet energy versus enzyme-like activities: the unexpected protection of palladium nanocrystals against oxidative damage. *ACS Nano* **10**, 10436-10445 (2016).

10 Singh, N. et al. Manganese-Based Nanozymes: Multienzyme Redox Activity and Effect on the Nitric Oxide Produced by Endothelial Nitric Oxide Synthase. *Chem. Eur. J.* **24**, 8393-8403 (2018).

11 Zhen, W. et al. Specific "unlocking" of a nanozyme-based butterfly effect to break the evolutionary fitness of chaotic tumors. *Angew. Chem. Int. Ed.* **59**, 9491-9497 (2020).

12 Liu, H. et al. Catalytically potent and selective clusterzymes for modulation of neuroinflammation through single-atom substitutions. *Nat. Commun.* **12**, 114 (2021).

13 A, G. K. et al. Efficiency of ab-initio total energy calculations for metals and semiconductors using a plane-wave basis set - ScienceDirect. *Comput. Mater. Sci.* **6**, 15-50 (1996).

14 Kresse, G. G. et al. Efficient Iterative Schemes for Ab Initio Total-Energy Calculations Using a Plane-Wave Basis Set. *Phys. Rev. B* **54**, 11169-11186 (1996).
15 Kresse, G. et al. Ab-Initio Molecular-Dynamics Simulation of The Liquid-Metal Amorphous-Semiconductor Transition In Germanium. *Phys. Rev. B* **49**, 14251-14269 (1994).
16 Blochl, P. E. Projector augmented-wave method. *Phys. Rev. B* **50**, 17953-17979 (1994).
17 Perdew, J. P. et al. Atoms, molecules, solids, and surfaces: Applications of the generalized gradient approximation for exchange and correlation. *Phys. Rev. B* **46**, 6671-6687 (1992).
18 Wang, V. et al. VASPKIT: A user-friendly interface facilitating high-throughput computing and analysis using VASP code. *Comput. Phys. Commun.* **267**, 108033 (2021).
