# Supplementary material for: Sono-activable and biocatalytic 3D-printed scaffolds for intelligently sequential therapies in osteosarcoma eradication and defect regeneration
Source: Nat Commun. 2025 Jul 4;16:6150. doi: 10.1038/s41467-025-61377-x (PMC12229518; doi:10.1038/s41467-025-61377-x)
Supplement: Supplementary file 2 — Description of Additional Supplementary Files [file 41467_2025_61377_MOESM2_ESM.pdf]

Description of Additional Supplementary Files

Supplementary Data 1: Atomic coordinates of the optimized computational models of ICTO.
