# Supplementary material for: Sono-activable and biocatalytic 3D-printed scaffolds for intelligently sequential therapies in osteosarcoma eradication and defect regeneration
Source: Nat Commun. 2025 Jul 4;16:6150. doi: 10.1038/s41467-025-61377-x (PMC12229518; doi:10.1038/s41467-025-61377-x)
Supplement: Supplementary file 4 — Reporting Summary [file 41467_2025_61377_MOESM4_ESM.pdf]

nature portfolio

Corresponding author(s): Chong Cheng

Last updated by author(s): Jun 19, 2025

Reporting Summary

Nature Portfolio wishes to improve the reproducibility of the work that we publish. This form provides structure for consistency and transparency in reporting. For further information on Nature Portfolio policies, see our Editorial Policies and the Editorial Policy Checklist.

Statistics

For all statistical analyses, confirm that the following items are present in the figure legend, table legend, main text, or Methods section.

n/a	Confirmed	
☐	☒	The exact sample size (*n*) for each experimental group/condition, given as a discrete number and unit of measurement
☐	☒	A statement on whether measurements were taken from distinct samples or whether the same sample was measured repeatedly
☐	☒	The statistical test(s) used AND whether they are one- or two-sided<br>*Only common tests should be described solely by name; describe more complex techniques in the Methods section.*
☒	☐	A description of all covariates tested
☐	☒	A description of any assumptions or corrections, such as tests of normality and adjustment for multiple comparisons
☐	☒	A full description of the statistical parameters including central tendency (e.g. means) or other basic estimates (e.g. regression coefficient) AND variation (e.g. standard deviation) or associated estimates of uncertainty (e.g. confidence intervals)
☐	☒	For null hypothesis testing, the test statistic (e.g. *F*, *t*, *r*) with confidence intervals, effect sizes, degrees of freedom and *P* value noted<br>*Give P values as exact values whenever suitable.*
☒	☐	For Bayesian analysis, information on the choice of priors and Markov chain Monte Carlo settings
☒	☐	For hierarchical and complex designs, identification of the appropriate level for tests and full reporting of outcomes
☒	☐	Estimates of effect sizes (e.g. Cohen's *d*, Pearson's *r*), indicating how they were calculated

Our web collection on statistics for biologists contains articles on many of the points above.

Software and code

Policy information about availability of computer code

Data collection	The surface morphology and dimensions analysis of materials were conducted with a ThermoFisherScientific Apreo S HiVoc scanning electron microscopy and a FEI Talos F200X transmission electron microscopy. Aberration-corrected high-angle annular dark-field scanning transmission electron microscopy and energy dispersive spectroscopy mapping were performed via FEI Titan Cubed Themis G2 300 and Titan Themis 60-300 operated at 200 kV. For ROS-ER stress, transmission electron microscopy was conducted using a Talos F200S microscope. The structure analysis of materials was measured by the Raman spectroscopy (XploRA PLUS, HORIBA), X-ray diffraction (DX-2700BH, HaoYuan Instrument, China), X-ray photoelectron spectroscopy (Thermo Scientific), and X-ray absorption spectroscopy (Shanghai Synchrotron Radiation Facility, China). The absorbance was detected by a multifunctional enzyme labeling instrument (ReadMax1900) and a Lamba 1050+ Ultraviolet-Visible-Near-Infrared (UV-Vis-NIR) spectrophotometer (PerkinElmer Lamba, USA). Free radical generation was determined by electron paramagnetic resonance (Bruker EPR EMX Plus, USA). Oxygen content was measured by a dissolved oxygen meter (JPSJ-606L, REX, China). Metal ion release kinetics was conducted using inductively coupled plasma mass spectrometry(Agilent Technologies, China). The mechanical properties were evaluated using a universal mechanical testing system (68TM-30, INSTRON, USA). The piezoelectric properties were evaluated using an oscilloscope (RIGOL DS1102 Z-E, RIGOL Technologies, China) and atomic force microscopy (AFM, MFP-3D-BIO, Asylum Research, USA). Fluorescence images and bright-field images were collected via a confocal laser scanning microscope (N-SIM S, Nikon, Olympus, Japan) or inverted microscope systems (ECLIPSE Ti2, Nikon, Japan). CCK-8 results were collected using the Multifunctional Microplate Reader (SYNERGY H1, Bio-Tek, USA). Flow cytometry data were collected via a flow cytometer (FACSAriaIII, BD Biosciences, USA). RNA concentration and purity were measured using NanoDrop 2000 (Thermo Fisher Scientific, Wilmington, DE, USA). RT-qPCR was performed using the real-time PCR system (QuantStudio® 3, Thermo Fisher Scientific, USA). Micro-CT images were collected using a micro-CT scanner (Quantum GX, PerkinElmer, USA). The libraries were sequenced on an Illumina NovaSeq 6000 platform. The pathology images were visualized using Vectra Polaris (PerkinElmer, USA).
Data analysis	Data analysis was conducted using GraphPad Prism Version 9.5, Origin 2024, VASP 5.4.1, MDI Jade 6, Avantage 5.9922, Athena software

	0.9.26, Artemis software 0.9.26, and Digital Micrograph 3.7.4. FlowJo Version 10.8.1 was used to analyze flow cytometry data. ImageJ Version 1.52v and Qupath 0.4.0 were used to analyze immunofluorescent and immunochemical data. All original schematic diagrams were created using the open-source software Blender 3.4 and Inkscape 1.4.2, both distributed under the GNU General Public License (GPL).

For manuscripts utilizing custom algorithms or software that are central to the research but not yet described in published literature, software must be made available to editors and reviewers. We strongly encourage code deposition in a community repository (e.g. GitHub). See the Nature Portfolio guidelines for submitting code & software for further information.

Data

Policy information about availability of data

All manuscripts must include a data availability statement. This statement should provide the following information, where applicable:

- Accession codes, unique identifiers, or web links for publicly available datasets
- A description of any restrictions on data availability
- For clinical datasets or third party data, please ensure that the statement adheres to our policy

The main data supporting the results of this study are available within the paper and its Supplementary Information. Any other raw data or noncommercial material used in this study are available from the corresponding author. Raw RNA sequencing data generated in this study have been deposited in the NCBI SRA database under accession number GSE296989 [https://www.ncbi.nlm.nih.gov/geo/query/acc.cgi?acc=GSE296989]. Source data are provided in this paper.

Research involving human participants, their data, or biological material

Policy information about studies with human participants or human data. See also policy information about sex, gender (identity/presentation), and sexual orientation and race, ethnicity and racism.

Reporting on sex and gender	N/A
Reporting on race, ethnicity, or other socially relevant groupings	N/A
Population characteristics	N/A
Recruitment	N/A
Ethics oversight	N/A

Note that full information on the approval of the study protocol must also be provided in the manuscript.

Field-specific reporting

Please select the one below that is the best fit for your research. If you are not sure, read the appropriate sections before making your selection.

☒ Life sciences ☐ Behavioural & social sciences ☐ Ecological, evolutionary & environmental sciences

For a reference copy of the document with all sections, see nature.com/documents/nr-reporting-summary-flat.pdf

Life sciences study design

All studies must disclose on these points even when the disclosure is negative.

Sample size	All biologically based assays were performed with the usual and sufficient sample size setting according to earlier paper (Nat. Commun. 2021, 12(1), 6143). These sample sizes were sufficient for a statistical analysis. All experiments reported here have n number and repetitions reported.
Data exclusions	No data was excluded from the analysis.
Replication	Results shown in the manuscript are representative of at least three independent experiments. All our attempts at replication were successful with similar results.
Randomization	Our samples/organisms were allocated randomly.
Blinding	In all experiments, investigators were blinded to group allocation during data collection and processing.

Reporting for specific materials, systems and methods

We require information from authors about some types of materials, experimental systems and methods used in many studies. Here, indicate whether each material, system or method listed is relevant to your study. If you are not sure if a list item applies to your research, read the appropriate section before selecting a response.

Materials & experimental systems

n/a	Involved in the study	
	☒	Antibodies
	☒	Eukaryotic cell lines
☒		Palaeontology and archaeology
	☒	Animals and other organisms
☒		Clinical data
☒		Dual use research of concern
☒		Plants

Methods

n/a	Involved in the study	
☒		ChIP-seq
	☒	Flow cytometry
☒		MRI-based neuroimaging

Antibodies

Antibodies used

Ki-67 rabbit monoclonal antibody (#12202, CST, USA, 1:200 dilution), Cleaved Caspase-3 rabbit monoclonal antibody (#9664, CST, USA, 1:200 dilution), HIF-1 alpha mouse monoclonal antibody (NB100-105, Novus Biologicals, USA, 1:50 dilution), RUNX2 rabbit polyclonal antibody (20700-1-AP , Proteintech, USA, 1:200 dilution), Recombination Anti-RUNX2 antibody (ab236639, Abcam, USA, 1:300 dilution), Collagen Type I rabbit polyclonal antibody (14695-1-AP, Proteintech, USA, 1:200 dilution), Anti-Collagen I antibody (ab34710, Abcam, USA, 1:200 dilution), Recombination Anti-BMP2 antibody (ab214821, Abcam, USA, 1:100 dilution for bone tissue staining and 1:200 for in vitro BMSCs staining), HRP-conjugated α-tubulin mouse monoclonal antibody (HRP-66031, Proteintech, USA, 1: 1:200 dilution), Fluorescein (FITC)–conjugated goat anti-Rabbit IgG (H+L) (SA00003-2, Proteintech, USA, 1: 1:1000 dilution), GAPDH mouse monoclonal antibody (T0004, Affinity, China, 1: 1:200 dilution), HIF-1 alpha rabbit polyclonal antibody (ab216842, Abcam, USA, 1:200 dilution), HRP-labeled goat anti-rabbit IgG(H+L) (A0208, Beyotime Biotech Inc, China, 1:500 dilutiuon), HRP-labeled goat anti-mouse IgG(H+L) (A0216, Beyotime Biotech Inc, China, 1:500 dilution), Alexa Fluor™ 647 donkey anti-rabbit IgG (H+L) (A32795, Invitrogen, USA, 1:500 dilutiuon), Goat anti-Rabbit IgG (H+L) Highly Cross-Adsorbed Secondary Antibody, Alexa Fluor™ 488 (A11034, Invitrogen, USA, 1:500 dilutiuon), Goat anti-Mouse IgG (H+L) Highly Cross-Adsorbed Secondary Antibody, Alexa Fluor™ 647 (A21236, Invitrogen, USA, 1:500 dilutiuon).

Validation

All antibodies were verified by the supplier. All validation statements of primary antibodies can be found on the respective antibody website:
Ki-67 rabbit monoclonal antibody: https://www.cellsignal.com/products/primary-antibodies/ki-67-d3b5-rabbit-mab-ihc-formulated/12202
Cleaved Caspase-3 rabbit monoclonal antibody: https://www.cellsignal.com/products/primary-antibodies/cleaved-caspase-3-asp175-5a1e-rabbit-mab/9664
HIF-1 alpha mouse monoclonal antibody: https://www.novusbio.com/products/hif-1-alpha-antibody-h1alpha67_nb100-105
RUNX2 rabbit polyclonal antibody: https://www.ptgcn.com/products/RUNX2-Antibody-20700-1-AP.htm
Recombination Anti-RUNX2 antibody: https://www.abcam.cn/products/primary-antibodies/runx2-antibody-epr22858-106-chip-grade-ab236639.html
Collagen Type I rabbit polyclonal antibody: https://www.ptgcn.com/products/COL1A2-Antibody-14695-1-AP.htm
Anti-Collagen I antibody: https://www.abcam.cn/products/primary-antibodies/collagen-i--collagen-iii-antibody-ab34710.html
Recombination Anti-BMP2 antibody: https://www.abcam.cn/products/primary-antibodies/bmp2-antibody-epr20807-ab214821.html
HRP-conjugated α-tubulin mouse monoclonal antibody: https://www.ptgcn.com/products/Tubulin-Alpha-Antibody-HRP-66031.htm
fluorescein (FITC)–conjugated goat anti-Rabbit IgG (H+L): https://www.ptgcn.com/products/Fluorescein-FITC-conjugated-Affinipure-Goat-Anti-Rabbit-IgG-H-L-secondary-antibody.htm
GAPDH mouse monoclonal antibody: https://www.affbiotech.cn/goods-6270-T0004-GAPDH_Antibody.html
HIF-1 alpha rabbit polyclonal antibody: https://www.abcam.cn/products/primary-antibodies/hif-1-alpha-antibody-ab216842.html
HRP-labeled goat anti-rabbit IgG(H+L): https://www.beyotime.com/product/A0208.htm
HRP-labeled goat anti-mouse IgG(H+L): https://www.beyotime.com/product/A0216.htm
Alexa Fluor™ 647 donkey anti-rabbit IgG (H+L): https://www.thermofisher.com/antibody/product/Donkey-anti-Rabbit-IgG-H-L-Highly-Cross-Adsorbed-Secondary-Antibody-Polyclonal/A-31573
Goat anti-Rabbit IgG (H+L) Highly Cross-Adsorbed Secondary Antibody, Alexa Fluor™ 488: https://www.thermofisher.com/antibody/product/Goat-anti-Rabbit-IgG-H-L-Highly-Cross-Adsorbed-Secondary-Antibody-Polyclonal/A-11034
Goat anti-Mouse IgG (H+L) Highly Cross-Adsorbed Secondary Antibody, Alexa Fluor™ 647: https://www.thermofisher.com/antibody/product/Goat-anti-Mouse-IgG-H-L-Highly-Cross-Adsorbed-Secondary-Antibody-Polyclonal/A-21236

Eukaryotic cell lines

Policy information about cell lines and Sex and Gender in Research

Cell line source(s)

The 143b human osteosarcoma cell line (Catalog No. CL-0007) and K7M2 murine osteosarcoma cell line (Catalog No. CL-0371) were obtained from Wuhan Pricella Biotechnology Co., Ltd (China). The RAW264.7 cell line (Catalog No. AMC1002) was purchased from Hangzhou Yangming Biotechnology Co., Ltd (China).

Authentication

The 143b, K7M2 and RAW264.7 cell line was validated by the supplier by short tandem repeat (STR) analysis.

Mycoplasma contamination

No contamination observed.

Commonly misidentified lines
(See ICLAC register)

No misidentified lines was used.

Animals and other research organisms

Policy information about studies involving animals; ARRIVE guidelines recommended for reporting animal research, and Sex and Gender in Research

Laboratory animals	4-week-old female BALB/c nude mice for the construction of subcutaneous osteosarcoma and 8-week-old male Sprague-Dawley rats for the construction of cranial bone defect. They were maintained under standardized laboratory conditions with regulated photoperiods (12:12 hour light-dark cycle, light phase: 8:00 a.m. to 8:00 p.m.). The subjects received unrestricted access to standard rodent chow (formulation 1010038, Jiangsu Xietong Pharmaceutical Co., Ltd.) and potable water. Environmental parameters were maintained within specified ranges (ambient temperature: 20-26°C; relative humidity: 40-70%).
Wild animals	The study did not involve wild animals.
Reporting on sex	Female mice and male rats were selected in this study. Sex was not considered in the study design.
Field-collected samples	The study did not involve samples collected from the field.
Ethics oversight	All animal experiments and associated procedures (including euthanasia) were conducted in accordance with the animal ethics guidelines of the Animal Ethics Committee at West China Hospital, Sichuan University, Chengdu, China, under the designated Animal Ethics Committee approval number 20220302062.

Note that full information on the approval of the study protocol must also be provided in the manuscript.

Plants

Seed stocks	N/A
Novel plant genotypes	N/A
Authentication	N/A

Flow Cytometry

Plots

Confirm that:

- [x] The axis labels state the marker and fluorochrome used (e.g. CD4-FITC).
- [x] The axis scales are clearly visible. Include numbers along axes only for bottom left plot of group (a 'group' is an analysis of identical markers).
- [x] All plots are contour plots with outliers or pseudocolor plots.
- [x] A numerical value for number of cells or percentage (with statistics) is provided.

Methodology

Sample preparation	143b cells were seeded onto HS-ICTO or HS in 48-well plates for 12 h, allowing cell attachment. Next, US irradiation was applied (1 W/cm2, 1.0 MHz, 30% duty cycle, 1 min). After another 12 h, the cells on the scaffolds were trypsinized and collected along with the cells in the supernatant. The Annexin V-FITC/PI Apoptosis Detection Kit (FXP018, 4A Biotech) was applied to stain the cells.
Instrument	FACSAriaIII, BD Biosciences, US
Software	FlowJo Version 10.8.1
Cell population abundance	At least 10,000 relevant events were acquired for all flow-cytometry analyses.
Gating strategy	All flow cytometry experiments employed this sample gating strategy. During the operation, Forward Scatter (FSC) and Side Scatter (SSC) dot plots were established, and the voltage was adjusted to ensure that all events fell within the visible range of the dot plots. Then, the events with appropriate FSC and SSC were gated based on HS and HS-ICTO group and collected. Followed by apoptosis-specific gating using Annexin-V/PI staining.

- [x] Tick this box to confirm that a figure exemplifying the gating strategy is provided in the Supplementary Information.
